# Do Amino-Oxetanes Resemble Amides? A Matched Molecular Pairs Property and Structural Comparison

**DOI:** 10.1021/acs.jmedchem.5c02614

**Published:** 2026-02-04

**Authors:** Hikaru Ishikura, Callum S. Begg, Juan J. Rojas, Luka Blagojevic, Gavin J. Smith, Joyce Luk, Rosemary A. Croft, Charles Romain, Chulho Choi, James A. Bull

**Affiliations:** † Department of Chemistry, Molecular Sciences Research Hub, 4615 Imperial College London, White City Campus, Wood Lane, London W12 0BZ, U.K.; ‡ Medicine Design, Pfizer Inc, Groton, Connecticut 06340, United States

## Abstract

Oxetanes display properties comparable to ketone carbonyl groups and are increasingly explored as bioisosteres. However, does the comparison hold for the most common carbonyl derivatives: do amino-oxetanes resemble amides? Here, we present a matched molecular pair study of 12 3-aryl-3-amino-oxetane and benzamide matched molecular pairs to assess their viability as isosteres. Across the surveyed physicochemical properties (pH stability, solubility, lipophilicity, clearance, permeability), amino-oxetanes exhibited broadly comparable profiles to their amide counterparts. Amino-oxetanes maintain both the H-bond acceptor and H-bond donor capabilities of analogous amides. These findings support the potential of amino-oxetanes as amide replacements. However, crystal structure analysis highlights the conformational differences and alternative exit vectors available through introduction of the oxetane ring. The preferred *gauche* conformation makes the torsion angle and exit vectors of amino-oxetanes more similar to sulfonamides, and therefore better like-for-like topological replacements. Overall, amino-oxetanes present an attractive design option to modulate physicochemical properties and chemical topology.

## Graphical abstract

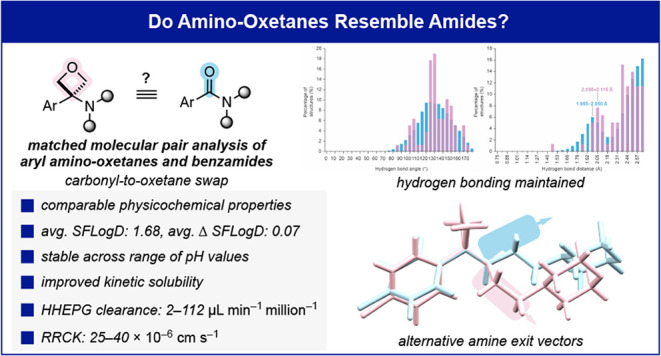

## Introduction

Oxetanes have been the subject of several studies on their potential in medicinal chemistry in recent years. This growing interest has contributed to the advancement of ten oxetane-containing compounds to clinical trials, marking the emergence of the first synthetic oxetane derivatives in the clinic and the first FDA approved fully synthetic oxetane containing drug (rilzabrutinib, approved September 2025).^1–3^ The low molecular weight, high polarity and three-dimensionality of oxetane motifs have offered distinct benefits through modulation of binding and physicochemical properties, as well as the potential to exploit novel chemical and intellectual property space. A prominent example is Ziresovir, where the inclusion of the oxetane was deemed the “highlight of the discovery” as it was able to attenuate amine basicity and thus volume of distribution, while maintaining biological activity through conformational control (Figure 1A).^4^ Carreira in collaboration with Hoffmann-La Roche pioneered the area through the use of oxetanes as a nonlipophilic alternative to *gem*-dimethyl groups to block metabolism of methylene units.^5^ Similarly, oxetanes can be considered as surrogates for carbonyl groups, maintaining comparable H-bonding ability, dipole moment, lone pair orientation, and polar surface area while eliminating an electrophilic center with potential issues of metabolic and chemical instability.^6–8^ Ongoing advances in synthetic methodology have further facilitated these applications in drug design and development.^9,10^ The exploration of oxetanes complements broader medicinal chemistry efforts to optimize key functional groups in drug design through bioisosteric replacements.^11^

**Figure 1:**
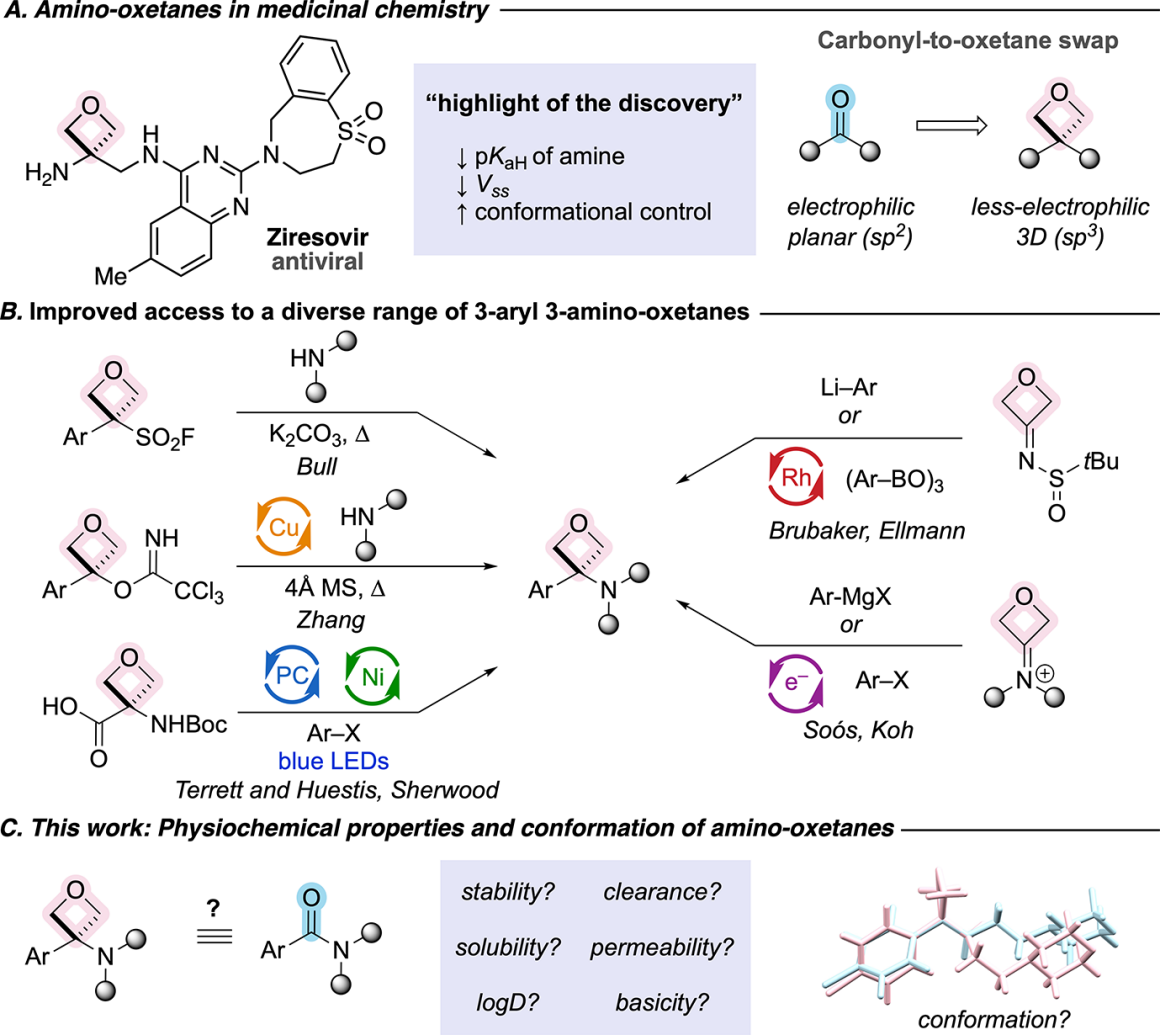
A. Examples of amino-oxetanes in medicinal chemistry. B. Synthetic strategies to access amino-oxetane derivatives. C. This work: comparison of the physicochemical properties and conformation of amino-oxetanes and amides.

In line with these efforts, there remains ongoing demand for alternative chemical motifs that expand accessible chemical space and provide beneficial physicochemical properties. Amides represent the most prevalent functional group in drug molecules. As a subset, benzamides are a commonly employed pharmacophore present in around 120 FDA-approved drug molecules.^12^ In this context, amino-oxetanes have been proposed as possible amide bioisosteres.^5,13^ Carreira and Shipman independently reported studies on the incorporation of oxetanes in peptidomimetics, where the carbonyl-to-oxetane replacement maintains bioactivity while resulting in improved stability against enzymatic metabolism.^14,15^ Notably, the conformational effect of the oxetane ring described in these peptide derivatives also facilitated macrocyclization.^15,16^

It is only recently that 3-aryl-3-amino-oxetanes have been synthetically available (Figure 1B). Early methodologies, including addition into oxetane sulfilimines and dual photoredox/nickel catalyzed arylation,^17,18^ afforded primary amino-oxetanes, which could be further functionalized.^19^ We reported the defluorosulfonylative coupling (deFS) reaction of oxetane sulfonyl fluorides with a diverse range of functionalized amine nucleophiles to afford 3-aryl-3-amino-oxetanes under mild condition in an amide-like disconnection.^20,21^ Similarly, Zhang employed Lewis acid activation of oxetanyl trichloroacetimidates to access these targets.^22^ Soós and Koh have developed complementary approaches exploiting oxetanone-derived iminium ions, reacting with Grignard reagents or undergoing electrochemical single-electron reduction to furnish secondary and primary 3-aryl amino-oxetanes, respectively.^23,24^

With improved access to these derivatives, we sought to evaluate whether amino-oxetanes function as effective amide mimics. Do the physicochemical properties or conformational features of amino-oxetanes align closely enough to amides that can support their use as isosteres? Alternatively, is a comparison with other amide isosteres more appropriate, or indeed should amino-oxetanes be better considered as a functional group with their own unique properties?

Here we make comparison with benzamides through the preparation of a series of arylamino-oxetane and benzamide matched molecular pairs (MMP) and analysis of their physiochemical properties including lipophilicity, chemical stability, solubility, clearance, and cell permeability (Figure 1C). We demonstrate that reasonable comparisons can be made with benzamides in terms of several important properties, however, 3-aryl amino-oxetanes adopt a distinct conformation, which differs significantly from that of a benzamide, and is much closer to that of sulfonamide derivatives.

## Results & Discussion

### Compound Design and Synthesis

We designed a series of amino-oxetane (**1a**–**12a**) and benzamide (**1b**–**12b**) matched molecular pairs with calculated properties to sit within a range of lead-like and drug-like chemical space.^25^ Two series were established: 1) a *p*-methoxyphenyl (PMP) series with varying amine components, and 2) a morpholine series with varying aromatic groups (Figure 2). This allowed for the interrogation of the effect of the carbonyl-to-oxetane replacement across a set of compounds spanning a range of MW, cSFlog*D*, and electronic features.^26^

**Figure 2:**
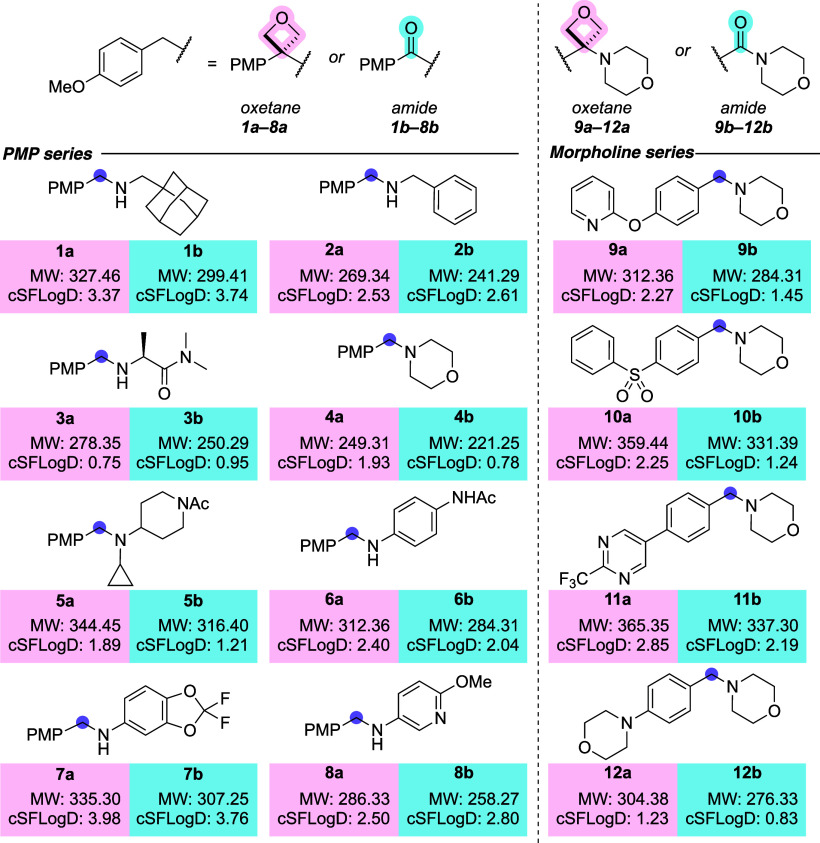
Amino-oxetane (**1a**–**12a**) and benzamide (**1b**–**12b**) matched molecular pairs. Calculated cSFlog*D* (pH = 7.4) values predicted using ACD/Laboratories, version 12.1. See SI for full calculated molecular properties.^26^

Amino-oxetanes **1a**–**8a** were synthesized in one-step from the PMP oxetane sulfonyl fluoride **13** and the corresponding amine through a deFS reaction (Scheme 1A).^20^ Amino-oxetanes **9a**–**12a** were synthesized from a common phenolic amino-oxetane intermediate **14a**, which in turn was derived from the deFS reaction of oxetane sulfonyl fluoride **15** with morpholine (Scheme 1B). As in our previous reports, in situ deprotection of the TIPS-group was observed to reveal the phenolic handle.^20^ From amino-oxetane **14a**, Ullmann condensation afforded amino-oxetane **9a**. Triflation of the phenol allowed for Suzuki–Miyaura and Buchwald–Hartwig cross-coupling to afford amino-oxetanes **11a** and **12a**, as well as Pd-catalyzed sulfonylation to give amino-oxetane **10a**. All synthetic transformations on amino-oxetane **14a** were well tolerated, in-line with their previously established chemical stability.^20^ Benzamides **1b**–**8b** were synthesized by amidation of *p*-methoxybenzoyl chloride **17** with the corresponding amine (Scheme 1A). Benzamides **9b**–**12b** were synthesized in a similar manner to amino-oxetanes **9a**–**12a** from commercially available amide **14b** through phenol derivatization (Scheme 1B).

**Scheme 1:**
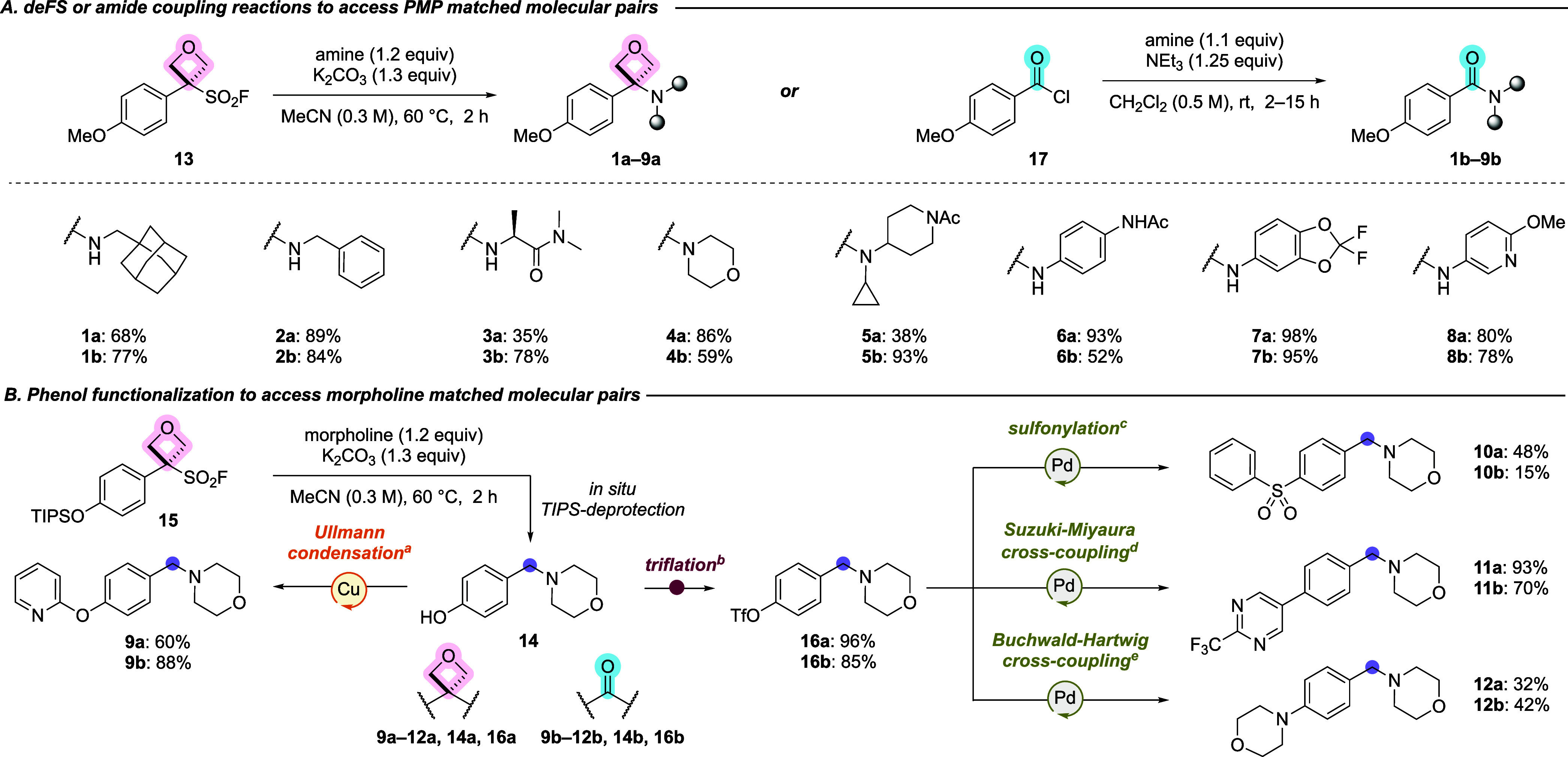
A. Synthesis of PMP-Aminooxetane and Amide Matched Molecular Pairs (**1**–**8**). B. Synthesis of Morpholine-Oxetane and Amide Matched Molecular Pairs (**9**–**12**). Compounds **1a**–**12a**, **13**, **14a**, and **15**, and **16a** Were Previously Reported (see reference 20)­^a^ ^b^ ^c^ ^d^

a CuI (10 mol %), picolinic acid (20 mol %), K_3_PO_4_ (2.2 equiv), 2-iodopyridine (1.3 equiv), DMSO (0.45 M), 110 °C, 24 h.
b Tf_2_O (1.1 equiv), pyridine (2.0 equiv), CH_2_Cl_2_ (0.5 M), rt, 3 h.
c Pd­(OAc)_2_ (10 mol %), Xantphos (10 mol %), Cs_2_CO_3_ (1.5 equiv), sodium benzenesulfinate salt (1.0 equiv), 1,4-dioxane (0.18 M), 110 °C, 16 h.
d For amino-oxetane: Pd­(PPh_3_)_4_ (3 mol %), Cs_2_CO_3_ (2.0 equiv), pyrimidine pinacol ester (1.5 equiv), 1,4-dioxane:water (7:3, 0.2 M), 110 °C, 20 h; for benzamide: Pd­(OAc)_2_ (10 mol %), SPhos (10 mol %), K_3_PO_4_ (2.0 equiv), pyrimidine pinacol ester (1.5 equiv), 1,4-dioxane:water (4:1, 0.1 M), 65 °C, 24 h.
e Pd­(OAc)_2_ (5 mol %), JohnPhos (10 mol %), K_3_PO_4_ (1.5 equiv), morpholine (1.2 equiv), THF (0.5 M), 65 °C, 24 h.

### Properties of a Set of Matched Molecular Pairs

First, we evaluated the lipophilicity (SFlog*D*, pH = 7.4) of the amino-oxetane and benzamide pairs. The amino-oxetanes exhibited a log*D* range of 0.33–3.60, with an average value of 1.68 across 10 compounds. In comparison, the benzamide counterparts showed a range of 0.44–3.83 and an average of 1.61. Although previous works on 3,3-disubstituted oxetanes have reported an increase in log*D* by +0.1 to +0.7 units,^5,6^ we found on average only a slight increase in log*D* of +0.07 units for the carbonyl-to-oxetane replacement (Figure 3).

**Figure 3:**
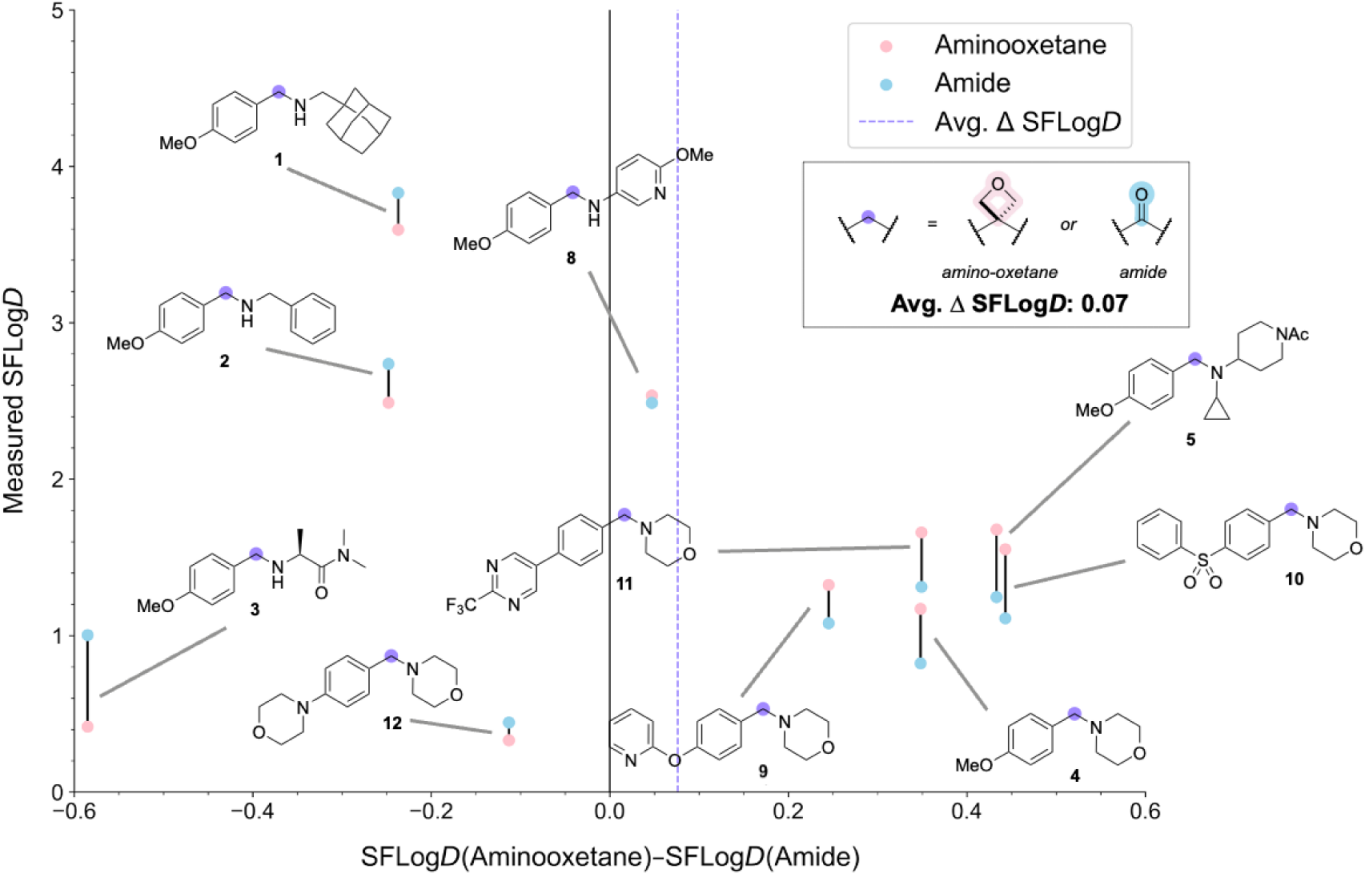
Change in SFLog*D* of the carbonyl-to-oxetane replacement plotted against the measured SFLog*D* for selected amino-oxetane (pink, **1a**–**5a** and **8a**–**12a**) and benzamide matched molecular pairs (blue, **1b**–**5b** and **8b**–**12b**). Zero on the *x*-axis represents no change in SFLog*D* from the replacement. Positive values on the *x*-axis represent an increase in SFLog*D* in the aminooxetane while negative values represent a decrease in SFLog*D* in the aminooxetane. The purple dotted line represents the average change in SFLog*D* from the carbonyl-to-oxetane replacement.

The effect on lipophilicity was heavily dependent on the nature of the peripheral substituents, for example, pairs **10a** and **10b** showed an increase in SFlog*D* of +0.44, whereas pairs **3a** and **3b** exhibited a decrease of −0.59 units.

Overall, this indicates that replacing benzamides with amino-oxetanes does not result in an unfavorable systematic increase in lipophilicity. The observed trends in SFlog*D* were generally well captured by the cSFlog*D*. For the PMP series, cSFlog*D* values were typically within 0.2 units of the experimental data. In contrast, for the morpholine series, while the predicted trends remained directionally consistent, deviations of around 1 unit were observed for the amino-oxetanes. Nevertheless, the ability of cSFlog*D* to reliably capture the general trends supports its utility as a quick and effective tool for predicting lipophilicity changes resulting from carbonyl-to-oxetane substitutions, despite the limited training data available for amino-oxetane structures.

We then subjected the pairs to a pH stability assay. Most of the surveyed pairs demonstrated good stability for 24 h under acidic, neutral, and basic conditions (Figure 4). Amino-oxetane **1a** exhibited reduced stability under neutral and basic conditions, however, this is likely due to solubility limitations in the assay rather than intrinsic chemical instability. Additionally, some degradation was also observed in the matched molecular pair **7**, which we speculate may be attributed to hydrolytic cleavage of the benzodioxole ring despite the stabilizing difluoride motif.^27^ These findings indicate that amino-oxetanes possess comparable chemical stability across a range of pH conditions to their benzamide analogues.

**Figure 4:**
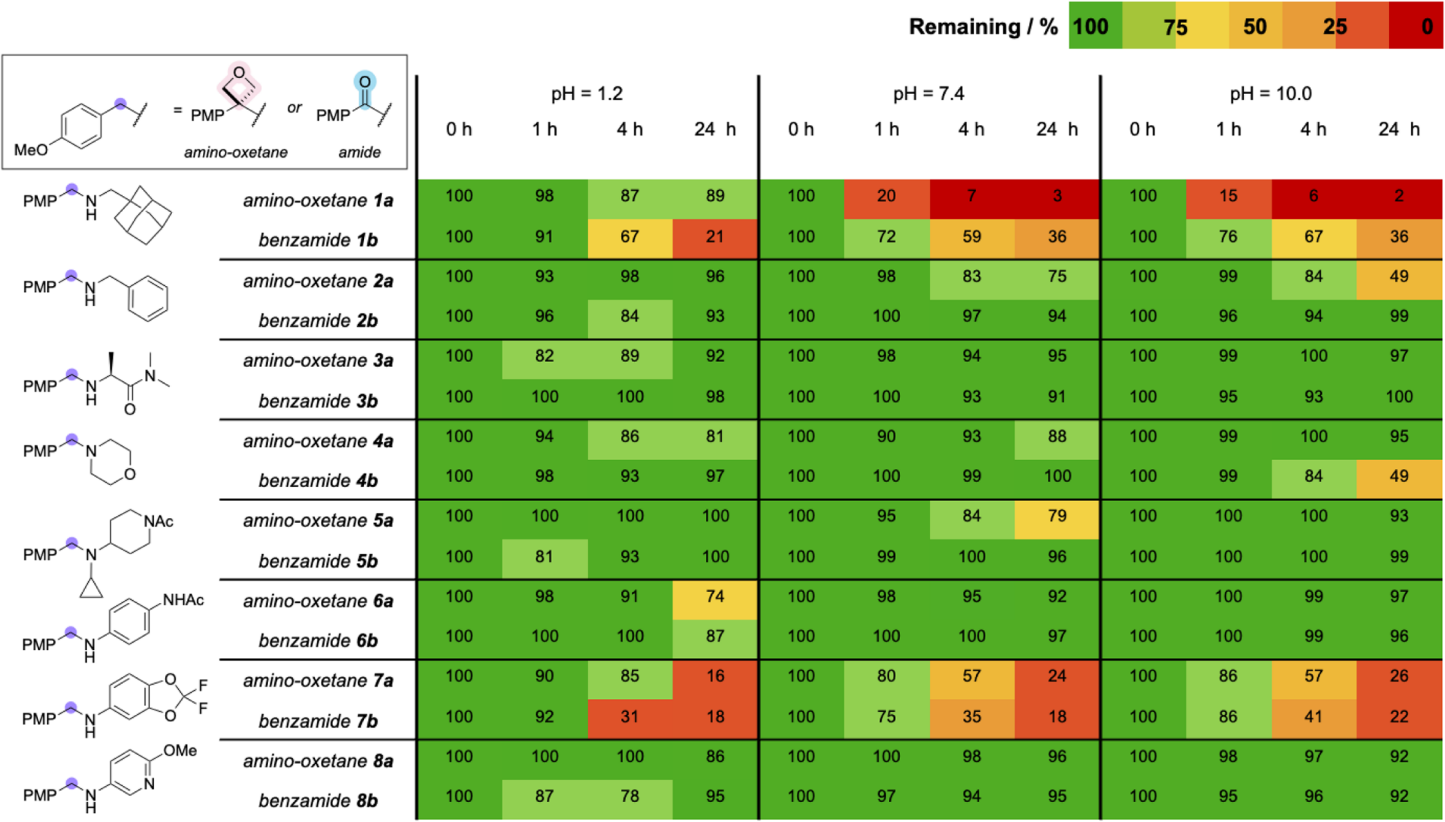
Stability of selected pairs (indicated by % of compound remaining) at different time points at different pH (1.2, 7.4, 10.9).

Kinetic solubility of selected amino-oxetanes and benzamides was subsequently assessed across acidic, neutral, and basic conditions (Figure 5). Although the aqueous solubility varied depending on the peripheral substituents, amino-oxetanes were generally more soluble than their benzamide counterparts, with the exception of amino-oxetane **5a**. Specifically, aniline-containing amino-oxetanes **6a** and **8a**, and benzylamine-containing amino-oxetane **2a** exhibited higher solubility across all tested pH values, presumably due to disruption of the conjugated π-system. Amino-oxetanes **3a** and **4a** displayed solubility comparable to their analogous benzamides **3b** and **4b**. Despite the overall low solubility of pair **1** under neutral and basic conditions, amino-oxetane **1a** showed markedly improved solubility at pH 3, likely due to protonation of the basic amine. Conversely, matched molecular pair **7** remained poorly soluble under all conditions tested. Taken together, these results suggest that amino-oxetanes offer a solubility advantage over benzamides in many cases, particularly under acidic conditions.^28^

**Figure 5:**
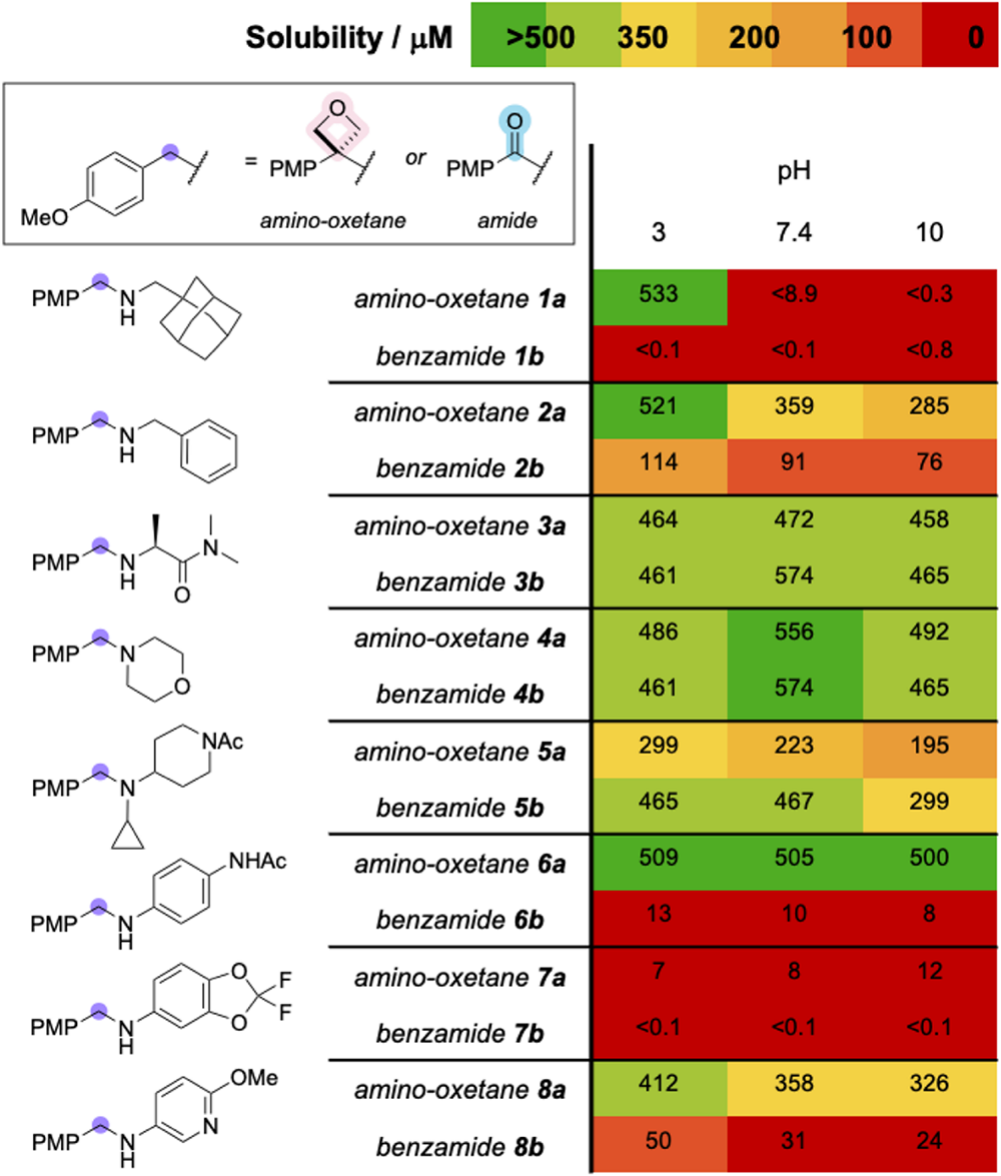
Kinetic solubility of selected pairs.

The influence of basicity is an ongoing rationale for inclusion of the oxetanes in drug candidates, providing the potential to attenuate off-target effects including hERG channel inhibition. In early studies, Carreira demonstrated that the inclusion of an oxetane in the α-, β-, or γ-position decreased neighboring amine basicity.^5c^ As comparison against the benzamide analogue is not possible, we instead looked at the head-to-head comparison between amino-oxetane **4a** and benzylamine **18** (Figure 6). Amino-oxetane **4a** was considerably less basic (p*K*_aH_ = 4.61, calculated = 5.44) compared to benzylamine **18** (p*K*_aH_ = 7.25, calculated = 6.68), clearly demonstrating the potential of oxetanes to attenuate amine basicity. Notably, the calculated p*K*_aH_ for both evaluated amino-oxetanes were similar to experimentally obtained values (within 1 p*K*_a_ unit) despite the lack of similar substructures within the database (Table S2 for further data).

**Figure 6:**
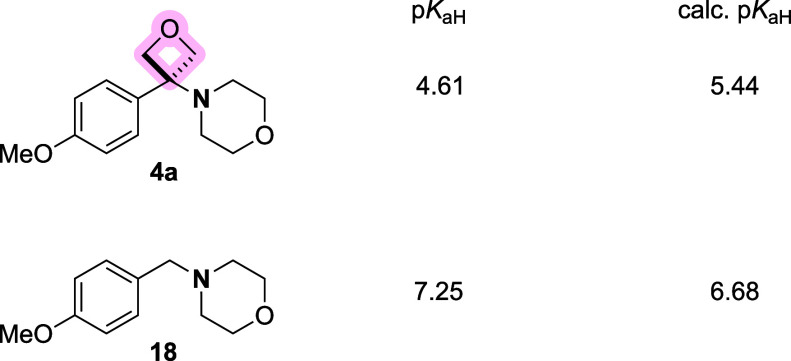
Comparison of p*K*_aH_ data.

Clearance by human hepatocytes (HHEPG) for selected amino-oxetane and benzamide pairs were generally within an acceptable range (<150 μL min^–1^ million^–1^, Figure 7). Little difference in HHEPG clearance was observed for most pairs with more pronounced changes between pairs in accordance with previous studies.^6^ Only amino-oxetane **7a** demonstrated a significantly higher clearance than its benzamide counterpart, although remaining within an acceptable range.

**Figure 7:**
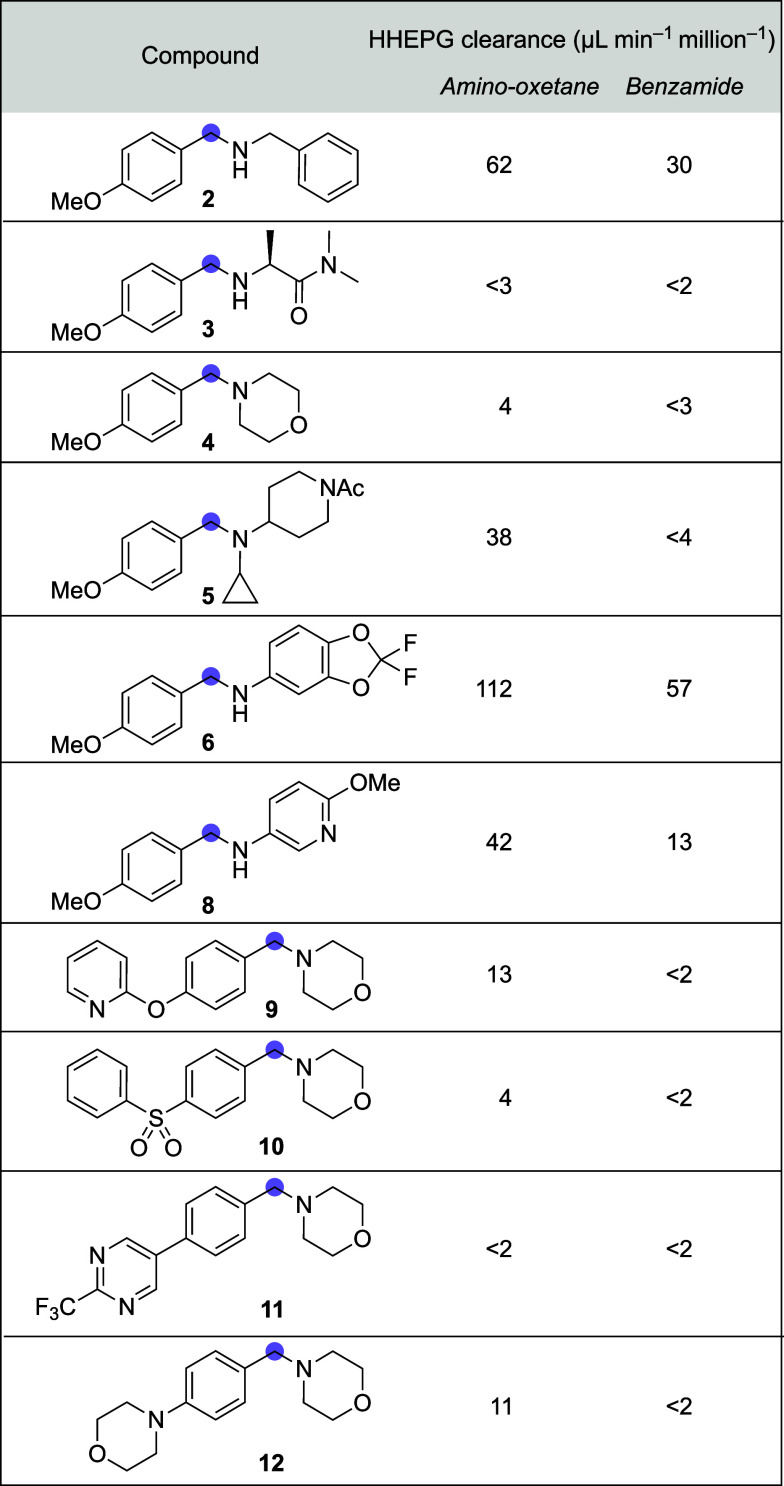
Clearance by human hepatocytes of selected pairs.

All pairs also demonstrated excellent permeability in a Ralph Russ Canine Kidney (RRCK) cell-based permeability assay. No significant difference was observed between the amino-oxetane and benzamide series, with values ranging from 25 to 50 × 10^–6^ cm s^–1^ (see Table S3).

Through analysis of the physicochemical properties, we have demonstrated that the amino-oxetane motif is not a liability and retains similar lipophilicity, chemical and metabolic stability, and permeability in comparison to benzamides. The carbonyl-to-oxetane swap simultaneously confers an increase in aqueous solubility. The introduction of an oxetane also reduces the p*K*_aH_ of neighboring amines allowing for its use to attenuate amine basicity.

### Conformational Comparisons

To investigate the three-dimensional conformations of arylamino-oxetanes, we began with a survey of X-ray motifs commonly regarded as amide isosteres in the Cambridge Structural Database (CSD), focused on amino-oxetanes, sulfonamides, and α-trifluoroethyl amines. Notable differences in torsion angles were revealed: while amides predominantly adopt predictable *cis* or *trans* conformations (ca. 0° or 180°), amino-oxetanes favor a *gauche* conformation, providing an alternative exit vector for the amine substituent (Figure 8A). Interestingly, the torsion angle distribution of amino-oxetanes more closely resembles sulfonamides than amides, whereas α-trifluoroethyl amines exhibit a more stochastic distribution.

**Figure 8:**
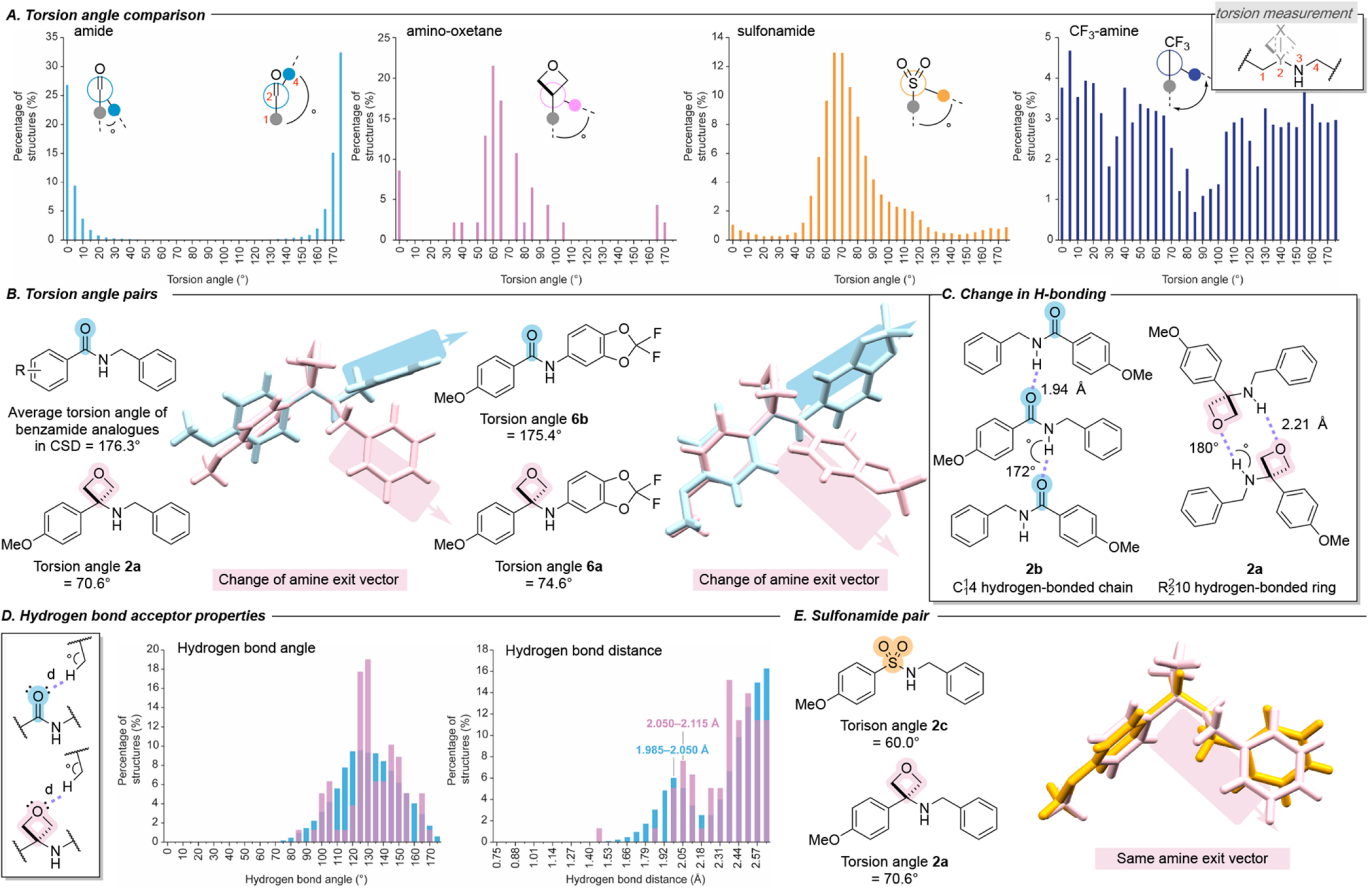
SCXRD analysis of amino-oxetane, benzamide, and sulfonamide structures. A. Torsion angles of commonly proposed amide bioisosteres in the CSD: amides, aminooxetanes, sulfonamides, and trifluoroethylamines. B. Torsion angles of pairs **2** and **6** determined by SCXRD. C. Change in hydrogen bonding conformation of amide and amino-oxetanes based on data available in the CSD. D. Hydrogen bond acceptor properties of amide and amino-oxetanes based on data available in the CSD. E. Torsion angles of sulfonamide and oxetane pair **2** determined by SCXRD.

To enable direct comparisons of the 3-aryl amino-oxetane functionality against benzamides, we recrystallized pairs **2** and **6** for analysis by single-crystal X-ray diffraction (SCXRD). Structural overlays (amide: blue; oxetane: pink; Figure 8B) highlight the conformational shift induced by introduction of the oxetane. Further analysis of *N*-benzyl benzamide derivatives bearing varying substituents on the benzamide ring confirmed that electronic effects exert minimal influence on torsion angles, especially in comparison to the influence of the carbonyl-to-oxetane swap (). While oxetanes cannot be conformationally considered direct isosteres of amides, they introduce orthogonal conformational control, including altered exit vectors, enabling the development of alternative electron density surfaces in drug design.

These conformational changes also affect hydrogen bonding morphology within the crystal structures (Figure 8C). For example, the amide **2b** forms a $C_{1}^{1}4$ hydrogen-bonded chain (NH···O: 1.94(2) Å), while the oxetane **2a** forms an $R_{2}^{2}10$ hydrogen-bonded ring (NH···O: 2.21(1) Å). Presumably the change from chain to ring is a consequence of the modified torsion angle between the pair, with the *gauche* conformation of the oxetane promoting the ring arrangement. These observations confirm that the aminooxetane can act both as an H-bond donor and acceptor, as would be expected for an amide.

Encouraged by these findings, we analyzed hydrogen bond acceptor angles and distances in the CSD. Similar angle and distance distributions were observed for hydrogen bonding to the oxetane oxygen versus the amide carbonyl oxygen (Figure 8D). There were only limited examples of amino-oxetane NH hydrogen bond donors in the CSD; hence the same analysis could not be performed.

To further compare torsion angles of oxetane and sulfonamide derivatives, we synthesized and recrystallized an analogous sulfonamide and amino-oxetane pair (**2a** and **2c**) and performed SCXRD analysis. The results showed that the torsion angle of the amino-oxetane (70.6°) more closely resembles that of the sulfonamide (60.0°), contrasting with the amide (176.4°, Figure 8E). By comparison to sulfonamides, amino oxetanes offer lower molecular weight and reduction of the NH acidity commonly associated with secondary sulfonamides. Indeed, secondary sulfonamides can be significantly ionized at physiological pH.^29^ In addition, amino oxetanes exhibit lower TPSA (TPSA = 30.49 and 55.40 Å^2^ for **2a** and **2c**). Taken together, these features suggest aminooxetanes may provide improvements to CNS penetration of acidic secondary sulfonamide derivatives, while maintaining key properties as described by CNS MPO (see Table S1 for further details).^30^ Moreover, sulfonamides display a slightly longer hydrogen bond and greater hydrogen bond angle, indicating a less pronounced H-bonding acceptor ability, which is distinct from the hydrogen bond properties seen in amides and aminooxetanes (see SI for details). These insights highlight the conformational properties of amino-oxetanes, which align more closely with sulfonamides while preserving the essential hydrogen-bonding interactions seen in amides. This supports their utility in drug design through orthogonal conformational control, access to novel structural opportunities, and as potential mimics for both amides and sulfonamides.

We next examined the barrier to rotation around the C–N bond computationally (Figure 9, B3LYP/6-311G+dp-GD3BJ; see SI for details). Amino-oxetanes demonstrated a low rotational barrier (<10 kcal mol^–1^, Figure 9A and see SI for other example aminooxetanes), which is indicative of free rotation in water.^31^

**Figure 9:**
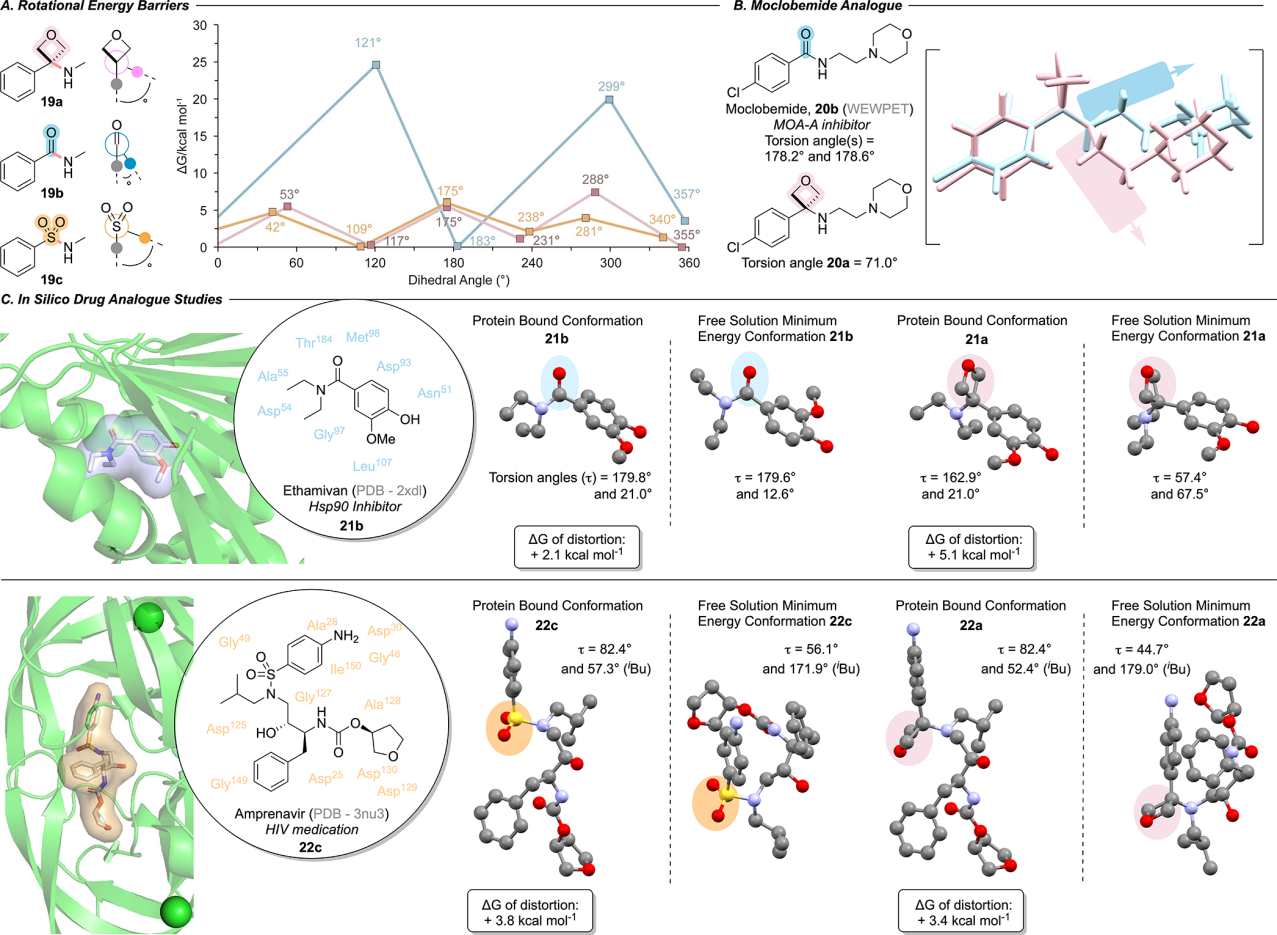
Computational studies on the molecular structure and rotational bond energies of amino-oxetanes, amides, and sulfonamides. A. Stable minima and transition states of rotation around the C–N bond in amino-oxetane **19a**, benzamide **19b**, and sulfonamide **19d**. Calculations performed at B3LYP/6-311G+dp-GD3BJ, SMD = water level of theory. Δ*G* quoted in kcal mol^–1^ compared to the lowest energy minima. B. Comparison of the torsion angles between moclobemide **20b** and oxetane analogue **20a** by comparison of single crystal X-ray structures (for synthesis of **20a** see reference 20b). C. Computational comparison of conformational energy penalty of ethamivan, amprenavir, and oxetane analogues **21a** and **22a** to adopt protein bound conformations. Torsion angles (τ) refer to the state of rotation around the C–N bond with respect to the plane through C_q(aromatic)_–C_q_–N.

Further NBO analysis of the nitrogen hybridization of the lowest energy conformations of aminooxetane **19a** indicated a pyramidal-like sp^2^–sp^3^ hybridization (sp^2.2^; vs sp^1.7^ for the benzamide **19b**). In contrast, benzamides displayed predictably high barriers to rotation (>20 kcal mol^–1^). Three minima were observed for both amino-oxetane **19a** and sulfonamide **19c**, indicative of the three staggered conformers, while benzamide **19b** preferred a periplanar conformation.

To directly compare the conformation of drug analogues, we synthesized and obtained a crystal structure of oxetanomoclobemide (**20a)**. Comparison with the crystal structure of moclobemide (**20b**) obtained from the CSD (WEWPET),^32^ again demonstrated the difference in preferred torsion angles around the central C–N bond in the solid state (Figure 9B).

The lower rotational barrier of aminooxetanes provides the potential to more flexibly accommodate binding sites in proteins of interest. The distortion energies from minimum energy conformation for ligands to adopt their bioactive conformations has previously been calculated to be ≤3 kcal mol^–1^ for around 70% of studied ligand-protein complexes.^33^ To provide a quantitative comparison, we investigated the in silico conformational energy penalty (Δ*G* of distortion) associated with adopting the bioactive (protein-bound) conformation of benzamide drug ethamivan (**21b**), and sulfonamide amprenavir (**22c**) and the oxetane analogues (**21a**, **22a**), with a fixed dihedral angle (Figure 9C and see SI for details). For the benzamide drug ethamivan **21b** (PDB: 2XDL),^34^ as expected minimal distortion in the amide was observed (2.1 kcal mol^–1^). For the corresponding aminooxetane **21a** to adopt this “planar” conformation as in the amide would cost 5.1 kcal mol^–1^. Such an increase in binding energy would suggest up to a 100-fold decrease in affinity.^33^

In contrast, for the sulfonamide drug amprenavir **22c** (PDB: 3NU3),^35^ the distortion energy in the binding conformation vs minimal energy conformation for the native sulfonamide was 3.8 kcal mol^–1^. Amino-oxetane analogue **22a** showed a very similar value of 3.4 kcal mol^–1^ indicating greater conformational compatibility and potential binding affinity compared to the carbonyl-to-oxetane swap.

## Conclusions

In summary, we have analyzed the physicochemical properties and conformation of amino-oxetanes, with direct comparisons to analogous benzamides. We have demonstrated that the amino-oxetanes are chemically stable under a range of pH values. No change in lipophilic profile, metabolic stability, or permeability is observed when compared to benzamide derivatives. In many cases, significant increases in solubility were observed by replacement of the carbonyl with an oxetane. Additionally, amine basicity was significantly reduced by introduction of an oxetane adjacent to an alkyl amine. Finally, we have highlighted the utility of calculated ADME values in predicting property changes from the carbonyl-to-oxetane replacement, even in the context of a limited training set of aminooxetanes. With improved synthetic access to these motifs, we anticipate further refinements in predictive models to support drug discovery campaigns.

Analysis of X-ray crystal structures of 3-aryl-3-amino-oxetanes and benzamides in the CSD has demonstrated the conformational shift induced by the carbonyl-to-oxetane swap. Amino-oxetanes adopt a *gauche* conformation, providing a distinct amine exit vector in comparison to the planar benzamides, while maintaining H-bonding ability. The torsion angles and exit vectors of the amino-oxetanes are similar to those of sulfonamides, potentially providing a new isosteric replacement.^36^ DFT calculations have also demonstrated amino-oxetanes to be freely rotating in water due to their low barrier of rotation, while benzamides remain conformationally restricted. Computational studies indicated that the distortion energy associated with aminooxetane drug analogues adopting the protein bound conformation of a benzamide drug is higher than that of adopting the conformation of a sulfonamide drug.

We conclude that although amino-oxetanes may not be a direct like-for-like replacement of amides, they maintain similar properties while providing an alternative chemical topology and unlocking the potential to explore new binding sites. We propose amino-oxetanes as interesting motifs in their own right, as well as potential sulfonamide isosteres, and an attractive design option to modulate physicochemical properties without introduction of inherent liabilities.

## Experimental Section

### General Experimental Considerations

All reactions were run under an inert atmosphere (Ar) with flame-dried glassware, using standard techniques unless otherwise specified. Anhydrous solvents were obtained by filtration through drying columns (CH_2_Cl_2_ and THF) or were purchased from Thermo Scientific Chemicals and used as supplied (MeOH, MeCN, DMSO, DMF, and 1,4-dioxane). Where stated, solvents were degassed by sparging with Ar for 30 min. Water for aqueous solutions and reaction quenches was deionized by electrodeionization using an Arium Advance EDI water purification system. Reactions in sealed tubes were run using Biotage microwave vials (0.5–2 mL, 2–5 mL, 10–20 mL) and aluminum caps with molded butyl/PTFE septa (1,4-dioxane, THF, DMSO, DMF) or simple butyl septa (MeCN). Liquid commercial amines were distilled over KOH pellets before use. Anhydrous K_2_CO_3_ (≥98%, powder, 325 mesh) was purchased from Sigma-Aldrich and flamedried before use. K_3_PO_4_ (≥98%) was purchased from Sigma-Aldrich and ground into a fine powder before use. Cs_2_CO_3_ (99%) was purchased from Sigma-Aldrich and oven-dried before use. All other commercial reagents were used as supplied or purified by standard techniques where necessary.

Manual flash column chromatography was performed using 230–400 mesh silica, with the indicated solvent system according to standard techniques. Automated flash column chromatography was carried out using a Biotage Selekt system with Biotage Sfär HC cartridges (5–25 g) under the indicated conditions. Visualization of the developed chromatogram was performed by UV absorbance (254 nm) and staining with aqueous potassium permanganate solution, aqueous cerium molybdate solution, or *p-*anisaldehyde in ethanol. Infrared spectra (*n*_max_, FTIRATR) were recorded in reciprocal centimeters (cm^–1^) using an Agilent Cary 630 FTIR spectrometer. Nuclear magnetic resonance spectra were recorded on 400 or 500 MHz spectrometers. Chemical shifts for ^1^H NMR spectra were recorded in parts per million (ppm) from tetramethylsilane with the residual protonated solvent resonance as the internal standard (CDCl_3_: δ 7.27 ppm, (CD_3_)_2_SO: δ 2.50 ppm). Data was reported as follows: chemical shift (multiplicity [s = singlet, d = doublet, t = triplet, q = quartet, m = multiplet, and br = broad], coupling constant (in Hz), integration, and assignment). All multiplet signals were quoted over a chemical shift range. ^13^C NMR spectra were recorded with complete proton decoupling. Chemical shifts were reported in parts per million from tetramethylsilane with the solvent resonance as the internal standard (^13^CDCl_3_: δ 77.0 ppm, (CD_3_)_2_SO: δ 39.5 ppm). Assignments of ^1^H and ^13^C spectra were based upon the analysis of d and *J* values. ^19^F NMR spectra were recorded with complete proton decoupling. Chemical shifts are reported in parts per million. ^19^F NMR spectra are indirectly referenced to CFCl_3_ automatically by direct measurement of the absolute frequency of the deuterium lock signal by the spectrometer hardware. Melting points were obtained using a Stuart SMP10 digital melting point apparatus and are uncorrected. High-resolution mass spectra (HRMS) were obtained through the Imperial College London mass spectrometry service. HRMS analyses were performed using an electrospray ion source (ESI) or atmospheric pressure chemical ionization (APCI) using an atmospheric solids analysis probe (ASAP). ESI was performed using a Waters LCT Premier (ES-TOF) equipped with an ESI source operated in positive ion mode or a Thermo Scientific Q-Exactive/Dionex Ultimate 3000. APCI was performed using a Thermo Scientific Q-Exactive/Dionex Ultimate 3000 using an ASAP to insert samples into the APCI source operated in positive or negative mode. The sample was introduced at ambient temperature and the temperature increased until the sample vaporized.

*p*-Methoxyphenyl oxetane sulfonyl fluoride **13** (PMP OSF) and ρ-triisopropylsiloxyphenyl oxetane sulfonyl fluoride **15** (OTIPS OSF),^20^ *t*-butyl (*S*)-(1-(dimethylamino)-1-oxopropan-2-yl)­carbamate **23**,^37^ 5-(4,4,5,5-tetramethyl-1,3,2-dioxaborolan-2-yl)-2-(trifluoromethyl)­pyrimidine **24**,^38^ and oxetane triflate **25** were synthesized according to previously reported conditions.^20b^ The purity of the synthesized compounds was determined by quantitative ^1^H NMR spectroscopy using 1,3,5-trimethoxybenzene (≥99%) as an internal standard and was at least of 95% purity unless otherwise stated.

General Procedure A: Anhydrous K_2_CO_3_ (1.3 equiv) was added to a reaction tube and flame-dried. Amine (1.2 equiv) and oxetane sulfonyl fluoride (1.0 equiv) were added sequentially and the reaction tube was sealed. Anhydrous acetonitrile (0.3 M) was added, and the reaction mixture was stirred at 60 °C for 2 h. After cooling to rt, the reaction mixture was diluted with EtOAc and filtered through a plug of Celite, eluting with further EtOAc. The solvent was then removed in vacuo. Purification by column chromatography under the stated conditions afforded the amino-oxetane.

General Procedure B: Methoxybenzoyl chloride **17** (68 μL, 0.50 mmol, 1.0 equiv) was added to a solution of amine (0.55 mmol, 1.1 equiv) and triethylamine (87 μL, 0.63 mmol, 1.3 equiv) in CH_2_Cl_2_ (1 mL, 0.5 M). After stirring at rt for 15 h, the reaction mixture was quenched with aq. HCl (1 M, 10 mL) and diluted with CH_2_Cl_2_ (10 mL). The layers were separated, and the aqueous layer was extracted with CH_2_Cl_2_ (3 × 10 mL). The combined organic layers were washed with brine (10 mL), dried over anhydrous Na_2_SO_4_, filtered, and concentrated in vacuo. Purification by column chromatography under the stated conditions afforded the benzamide.

#### *N*-(((3*R*,5*R*,7*R*)-Adamantan-1-yl)­Methyl)-3-(4-Methoxyphenyl)­Oxetan-3-Amine (**1a**)

Prepared according to General Procedure A using K_2_CO_3_, (36.0 mg, 0.26 mmol, 1.3 equiv), 1-adamantanemethylamine (43 μL, 0.24 mmol, 1.2 equiv) and PMP OSF **13** (49.2 mg, 0.2 mmol, 1.0 equiv). Purification by flash column chromatography (10% EtOAc/hexane) afforded amino-oxetane **1a** as a colorless oil (45.0 mg, 68%). R_*f*_ = 0.18 (10% EtOAc/hexane); ^1^H NMR (400 MHz, CDCl_3_) δ 7.29–7.25 (m, 2H, 2 × Ar–CH), 6.95–6.90 (m, 2H, 2 × Ar–CH), 4.94 (dd, *J* = 6.2, 0.6 Hz, 2H, CHHOCHH), 4.74 (dd, *J* = 6.2, 0.6 Hz, 2H, CHHOCHH), 3.82 (s, 3H, OCH_3_), 3.16–3.08 (m, 1H, CH), 2.09–2.02 (m, 2H, NCH­(CHH)_2_), 1.96 (br s, 1H, NH), 1.74–1.45 (m, 4H, NCH­(CH_2_)_2_CH_2_ + NCH­(CHH)_2_). The observed characterization data (R_*f*_, ^1^H NMR) was consistent with that previously reported.^20a^

#### *N*-(((3*R*,5*R*,7*R*)-Adamantan-1-yl)­Methyl)-4-Methoxybenzamide (**1b**)

Prepared according to General Procedure B using ((3*r*,5*r*,7*r*)-adamantan-1-yl)­methanamine (97 μL, 0.55 mmol, 1.1 equiv). Purification by flash column chromatography (1% MeOH/CH_2_Cl_2_) afforded benzamide **1b** as a white solid (115 mg, 77%). R_*f*_ = 0.22 (1% MeOH/CH_2_Cl_2_); mp = 195–196 °C; IR (film)/cm^–1^ 3354 (NH), 2892, 2836, 1632 (C=O), 1600, 1543, 1505, 1256, 1174, 1036, 838, 767; ^1^H NMR (400 MHz, CDCl_3_) δ 7.77–7.72 (m, 2H, 2 × Ar–CH), 6.96–6.91 (m, 2H, 2 × Ar–CH), 6.12 (s, 1H, NH), 3.85 (s, 3H, OCH_3_), 3.15 (d, *J* = 6.4 Hz, 2H, CH2N), 2.00 (s, 3H, 3 × CH), 1.76–1.69 (m, 3H, 3 × CHH), 1.68–1.69 (m, 3H, 3 × CHH), 1.56 (d, *J* = 2.4 Hz, 6H, 3 × CH_2_); ^13^C NMR (101 MHz, CDCl_3_) δ 167.2 (C=O), 162.0 (ArC_q_OMe), 128.6 (2 × Ar–CH), 127.3 (Ar–C_q_C=O), 113.7 (2 × Ar–CH), 55.4 (OCH_3_), 51.3 (CH_2_N), 40.3 (3 × CH_2_), 36.9 (3 × CH_2_), 34.0 (C_q_), 28.2 (3 × CH); HRMS (TOF-MS-ES^+^) *m*/*z* calcd for C_19_H_26_NO_2_^+^ [M + H]^+^: 300.1958; found 300.1962.

#### *N*-Benzyl-3-(4-Methoxyphenyl)­Oxetan-3-Amine (**2a**)

Prepared according to General Procedure A using K_2_CO_3_, (36.0 mg, 0.26 mmol, 1.3 equiv), benzylamine (43 μL, 26 μL, 1.2 equiv) and PMP OSF **13** (49.2 mg, 0.2 mmol, 1.0 equiv). Purification by flash column chromatography (40% EtOAc/hexane) afforded amino-oxetane **2a** as a white solid (48.0 mg, 89%). R_*f*_ = 0.29 (40% EtOAc/hexane); ^1^H NMR (400 MHz, CDCl_3_) δ 7.44–7.39 (m, 2H, 2 × Ar–CH), 7.36–7.30 (m, 4H, 4 × Ar–CH), 7.29–7.23 (m, 1H, Ar–CH), 6.99–6.94 (m, 2H, 2 × Ar–CH), 4.97 (d, *J* = 6.4 Hz, CHHOCHH), 4.77 (d, *J* = 6.4 Hz, CHHOCHH), 3.85 (s, 3H, OCH_3_), 3.56 (s, 2H, CH_2_Ph), 2.05 (br s, 1H, NH). The observed characterization data (R_*f*_, ^1^H NMR) was consistent with that previously reported.^20a^

#### *N*-Benzyl-4-Methoxybenzamide (**2b**)

Prepared according to General Procedure B using benzylamine (60 μL, 0.55 mmol, 1.1 equiv). Purification by flash column chromatography (5% Et_2_O/CH_2_Cl_2_) afforded benzamide **2b** as a white solid (102 mg, 84%). R_*f*_ = 0.32 (5% Et_2_O/CH_2_Cl_2_); mp = 134–136 °C, IR (film)/cm^–1^ 3254 (NH), 1628 (C=O), 1609, 1558, 1498, 1323, 1248, 1174, 987, 824, 723, 685; ^1^H NMR (400 MHz, CDCl3) δ: 7.80–7.74 (m, 2H, 2 × Ar–CH), 7.39–7.26 (m, 5H, 5 × Ar–CH), 6.94–6.89 (m, 2H, 2 × Ar–CH), 6.45 (s, 1H, NH), 4.63 (d, *J* = 5.7 Hz, 2H, CH_2_Ph), 3.85 (s, 3H, OCH_3_); ^13^C NMR (101 MHz, CDCl_3_) δ: 166.8 (C=O), 162.2 (Ar–C_q_OMe), 138.4 (Ar–C_q_CH_2_), 128.7 (2 × Ar–CH), 128.7 (2 × Ar–CH), 127.9 (2 × Ar–CH), 127.5 (Ar–CH), 126.6 (Ar–C_q_C=O), 113.7 (2 × Ar–CH), 55.4 (OCH_3_), 44.0 (CH_2_). The observed characterization data (^1^H NMR, ^13^C NMR) was consistent with that previously reported.^39^

#### *N*-Benzyl-4-Methoxybenzenesulfonamide (**2c**)

Et_3_N (0.16 mL, 2.2 mmol, 1.7 equiv) and benzylamine (0.15 mL, 1.4 mmol, 1.05 equiv) were added dropwise to a solution of 4-methoxybenzenesulfonyl chloride (276 mg, 1.3 mmol, 1.0 equiv) in anhydrous CH_2_Cl_2_ (10 mL, 0.1 M) at 0 °C. After stirring at rt for 16 h, the solvent was removed in vacuo. Purification by recrystallization (EtOH) afforded sulfonamide **2c** as clear colorless solid blocks (366 mg, 99% yield). R_*f*_ = 0.25 (20% EtOAc/pentane); mp = 105–106 °C; IR (film)/cm^–1^ 2880, 2600, 1724, 1597, 1497, 1323, 1258, 1154, 1096, 833, 563; ^1^H NMR (400 MHz, MeOD) δ 7.82–7.71 (m, 2H, 2 × Ar–CH), 7.29–7.16 (m, 5H, 5 × Ar–CH), 7.07–6.98 (m, 2H, 2 × Ar–CH), 4.01 (s, 2H, CH_2_Ph), 3.85 (s, 3H, OCH_3_); ^13^C NMR (101 MHz, MeOD) δ 164.3 (ArC_q_OMe), 138.7 (ArC_q_CH_2_), 133.5 (ArC_q_SO_2_), 130.1 (2 × Ar–CH), 129.4 (2 × Ar–CH), 128.9 (2 × Ar–CH), 128.4 (Ar–CH), 115.2 (2 × Ar–CH), 56.2 (OCH_3_), 47.9 (CH_2_Ph). The observed characterization data (R_*f*_, ^1^H, ^13^C) were consistent with that previously reported.^40^

#### (*S*)-2-((3-(4-Methoxyphenyl)­Oxetan-3-yl)­Amino)-*N*,*N*-Dimethylpropanamide (**3a**)

Trifluoroacetic acid (2.7 mL, 35 mmol, 10 equiv) was added dropwise to a solution of Boc-protected amine **23** (756 mg, 3.5 mmol, 1.0 equiv) in CH_2_Cl_2_ (18 mL, 0.2 M) at 0 °C. The reaction mixture was stirred at 0 °C for 10 min, warmed to 25 °C and stirred for a further 2.5 h. Concentration in vacuo afforded 1-(dimethylamino)-1-oxopropan-2-aminium trifluoroacetate (810 mg) of which a portion was used in the next step without further purification. Anhydrous K_2_CO_3_ (77.4 mg, 0.56 mmol, 2.8 equiv) was added to a reaction tube and flame-dried. (*S*)-1-(Dimethylamino)-1-oxopropan-2-aminium trifluoroacetate (58.1 mg, 0.25 mmol, 1.25 equiv) and PMP OSF **13** (49.2 mg, 0.20 mmol, 1.0 equiv) were added sequentially and the reaction tube was sealed. Anhydrous acetonitrile (0.66 mL, 0.3 M) was added, and the reaction mixture was stirred at 60 °C for 2 h. After cooling to rt, the reaction mixture was diluted with EtOAc (5 mL) and filtered through a plug of Celite, eluting with further EtOAc (3 × 10 mL). The solvent was then removed in vacuo. Purification by flash chromatography (50% acetone/hexane) afforded amino-oxetane **3a** as a colorless oil (19.7 mg, 35%). R_*f*_ = 0.20 (50% acetone/hexane); ^1^H NMR (400 MHz, CDCl_3_) δ 7.32–7.28 (m, 2H, 2 × Ar–CH), 6.94–6.90 (m, 2H, 2 × Ar–CH), 4.93 (d, *J* = 6.4 Hz, 1H, CHHOCH_2_), 4.84 (d, *J* = 6.6 Hz, 1H, CHHOCH_2_), 4.82 (d, *J* = 6.6 Hz, 1H, CH_2_OCHH), 4.72 (d, *J* = 6.4 Hz, 1H, CH_2_OCHH), 3.83 (s, 3H, OCH_3_), 3.42 (q, *J* = 6.9 Hz, 1H, CH), 2.88 (s, 3H, NCH_3_), 2.74 (s, 3H, NCH_3_), 1.75 (br s, 1H, NH), 1.13 (d, *J* = 6.9 Hz, 3H, CHCH_3_). The observed characterization data (R_*f*_, ^1^H NMR) was consistent with that previously reported.^20a^

#### *N*-(1-(Dimethylamino)-1-Oxopropan-2-yl)-4-Methoxybenzamide (**3b**)

Trifluoroacetic acid (2.7 mL, 35 mmol, 10 equiv) was added dropwise to a solution of Bocprotected amine **23** (756 mg, 3.5 mmol, 1.0 equiv) in CH_2_Cl_2_ (18 mL, 0.2 M) at 0 °C. The reaction mixture was stirred at 0 °C for 10 min, warmed to 25 °C and stirred for a further 2.5 h. Concentration in vacuo afforded 1-(dimethylamino)-1-oxopropan-2-aminium trifluoroacetate (810 mg) of which a portion was used in the next step without further purification. Methoxybenzoyl chloride (68 μL, 0.50 mmol, 1.0 equiv) was added to a solution of 1-(dimethylamino)-1-oxopropan-2-aminium trifluoroacetate (127 mg, 0.55 mmol, 1.1 equiv) and triethylamine (174 μL, 1.25 mmol, 2.5 equiv) in CH_2_Cl_2_ (1 mL, 0.5 M). After stirring at rt for 14 h, the reaction mixture was quenched with aq. HCl (1 M, 10 mL) and diluted with CH_2_Cl_2_ (10 mL). The layers were separated, and the aqueous layer was extracted with CH_2_Cl_2_ (3 × 10 mL). The combined organic layers were washed with brine (10 mL), dried over anhydrous Na_2_SO_4_, filtered, and concentrated in vacuo. Purification by flash chromatography (50% acetone/hexane) afforded benzamide **3b** as a colorless paste (98.0 mg, 78%). R_*f*_ = 0.25 (50% acetone/hexane); IR (film)/cm^–1^ 3317 (NH), 2937, 1625 (C=O), 1620 (C=O), 1494, 1304, 1248, 1177, 1107, 1028, 846, 767, 730; ^1^H NMR (400 MHz, CDCl_3_) δ 7.82–7.77 (m, 2H, 2 × Ar–CH), 7.24 (d, *J* = 6.9 Hz, 1H, NH), 6.95–6.90 (m, 2H, 2 × Ar–CH), 5.09 (p, *J* = 6.9 Hz, 1H, CH), 3.86 (s, 3H, OCH_3_), 3.13 (s, 3H, NCH_3_), 3.02 (s, 3H, NCH_3_), 1.44 (d, *J* = 6.8 Hz, 3H, CH_3_); ^13^C NMR (101 MHz, CDCl_3_) δ 172.5 (C=O), 165.7 (C=O), 162.0 (ArC_q_OMe), 128.7 (2 × Ar–CH), 126.1 (Ar–C_q_C=O), 113.4 (2 × Ar–CH), 55.2 (OCH_3_), 45.4 (CH), 36.8 (NCH_3_), 35.6 (NCH_3_), 18.5 (CH_3_); HRMS (TOF-MS-ES^+^) *m*/*z* calcd for C_13_H_19_N_2_O_3_^+^ [M + H]^+^: 251.1390; found 251.1394.

#### 4-(3-(4-Methoxyphenyl)­Oxetan-3-yl)­Morpholine (**4a**)

Prepared according to General Procedure A using K_2_CO_3_, (36.0 mg, 0.26 mmol, 1.3 equiv), morpholine (21 μL, 0.24 mmol, 1.2 equiv) and PMP OSF **13** (49.2 mg, 0.2 mmol, 1.0 equiv). Purification by flash column chromatography (80% EtOAc/hexane) afforded amino-oxetane **4a** as a white solid (43.0 mg, 86%). R_*f*_ = 0.21 (80% EtOAc/hexane); ^1^H NMR (400 MHz, CDCl_3_) δ 7.00–6.96 (m, 2H, 2 × Ar–CH), 6.94–6.88 (m, 2H, 2 × Ar–CH), 4.90 (d, *J* = 5.9 Hz, 2H, CHHOCHH), 4.87 (d, *J* = 5.9 Hz, 2H, CHHOCHH), 3.83 (s, 3H, OCH_3_), 3.74 (t, *J* = 4.6 Hz, 4H, CH_2_CH_2_OCH_2_CH_2_), 2.31 (t, *J* = 4.6 Hz, 4H, CH_2_NCH_2_). The observed characterization data (R_*f*_, ^1^H NMR) was consistent with that previously reported.^20a^

#### (4-Methoxyphenyl)­(Morpholino)­Methanone (**4b**)

Prepared according to General Procedure B using morpholine (48 μL, 0.55 mmol, 1.1 equiv). Purification by flash column chromatography (60% EtOAc/pentane) afforded benzamide **4b** as a colorless paste (65.0 mg, 59%). R_*f*_ = 0.20 (60% EtOAc/pentane); IR (film)/cm^–1^ 2959, 2918, 2851, 1625 (C=O), 1513, 1420, 1245, 1170, 1110, 1021, 839; ^1^H NMR (400 MHz, CDCl_3_) δ 7.43–7.37 (m, 2H, 2 × Ar–CH), 6.96–6.90 (m, 2H, 2 × Ar–CH), 3.84 (s, 3H, OCH_3_), 3.80–3.51 (br s, 8H, 4 × CH2); ^13^C NMR (101 MHz, CDCl_3_) δ 170.3 (C=O), 160.8 (Ar–C_q_OMe), 129.1 (2 × Ar–CH), 127.3 (Ar–C_q_C=O), 113.7 (2 × Ar–CH), 66.8 (CH_2_OCH_2_), 55.3 (CH_3_), 47.8 (br, CH_2_N), 43.7 (br, CH_2_N). The observed characterization data (^1^H NMR, ^13^C NMR) was consistent with that previously reported.^41^

#### 1-(4-(Cyclopropyl­(3-(4-Methoxyphenyl)­Oxetan-3-yl)­Amino)­Piperidin-1-yl)­Ethan-1-One (**5a**)

Prepared according to General Procedure A using K_2_CO_3_, (36.0 mg, 0.26 mmol, 1.3 equiv), 1-(4-(cyclopropylamino)­piperidin-1-yl)­ethan-1-one (43.7 mg, 0.24 mmol, 1.2 equiv) and PMP OSF **13** (49.2 mg, 0.2 mmol, 1.0 equiv). Purification by flash column chromatography (40% acetone/hexane) afforded amino-oxetane **5a** as a white solid (26.4 mg, 38%). R_*f*_ = 0.24 (40% acetone/hexane);^1^H NMR (400 MHz, CDCl_3_) δ 7.42–7.29 (m, 2H, 2 × Ar–CH), 7.08–6.83 (m, 2H, 2 × Ar–CH), 5.13–4.99 (m, 2H, CHHOCHH), 4.80 (d, *J* = 5.7 Hz, 1H, CHHOCH_2_), 4.78 (d, *J* = 5.7 Hz, 1H, CH_2_OCHH), 4.56 (ddt, *J* = 13.3, 4.4, 2.4 Hz, 1H, CHHNCH_2_), 3.83 (s, 3H, OCH_3_), 3.70 (ddt, *J* = 13.6, 4.2, 2.4 Hz, 1H, CH_2_NCHH), 2.86 (td, *J* = 13.2, 2.1 Hz, 1H, CHHNCH_2_), 2.52 (tt, *J* = 11.8, 3.6 Hz, 1H, CH), 2.33 (ddt, *J* = 13.3, 4.4, 2.4 Hz, 1H, CH_2_NCHH), 2.03 (s, 3H, C­(O)­CH_3_), 1.72–1.65 (m, 1H, CH), 1.61–1.48 (m, 2H, 2 × CHHCH), 1.34 (d, *J* = 12.6 Hz, 2H, 2 × CHHCH), 0.59–0.47 (m, 4H, 2 × CH_2_). The observed characterization data (R_*f*_, ^1^H NMR) was consistent with that previously reported.^20a^

#### *N*-(1-Acetylpiperidin-4-yl)-*N*-Cyclopropyl-4-Methoxybenzamide (**5b**)

Prepared according to General Procedure B using 1-(4-(cyclopropylamino)­piperidin-1-yl)­ethan-1-one (100 mg, 0.55 mmol, 1.1 equiv). Purification by flash column chromatography (5% MeOH/CH_2_Cl_2_) afforded benzamide **5b** as a white solid (147 mg, 93%). R_*f*_ = 0.15 (5% MeOH/CH_2_Cl_2_); mp = 104–105 °C; IR (film)/cm^–1^ 2959, 1632 (C=O), 1602 (C=O), 1513, 1420, 1364, 1326, 1241, 1172, 1021, 834, 764, 715; ^1^H NMR (400 MHz, CDCl_3_) δ 7.49–7.44 (m, 2H, 2 × Ar–CH), 6.91–6.86 (m, 2H, 2 × Ar–CH), 4.79 (ddd, *J* = 6.5, 3.9, 2.1 Hz, 1H, NCHH), 4.42–4.32 (m, 1H, CH), 3.95–3.87 (m, 1H, NCHH), 3.84 (s, 3H, OCH_3_), 3.23–3.12 (m, 1H, NCHH), 2.67–2.54 (m, 2H, NCHH + CH), 2.13 (s, 3H, CH_3_), 1.96 (ddd, *J* = 24.0, 12.4, 4.2 Hz, 4H, 2 × CH_2_CH), 0.70–0.53 (m, 2H, 2 × CHH), 0.50–0.35 (m, 2H, 2 × CHH); ^13^C NMR (101 MHz, CDCl_3_) δ 172.5 (C=O), 168.8 (C=O), 160.6 (Ar–C_q_OMe), 129.9 (Ar–C_q_C=O), 129.4 (2 × Ar–CH), 113.1 (2 × Ar–CH), 56.4 (CH), 55.3 (OCH_3_), 46.2 (NCH_2_), 41.4 (NCH_2_), 31.1 (CHCH_2_), 30.0 (CHCH_2_), 28.8 (CH), 21.4 (CH_3_), 10.1 (CH_2_), 10.0 (CH_2_); HRMS (TOFMSES^+^) *m*/*z* calcd for C_18_H_25_N_2_O_3_^+^ [M + H]^+^: 317.1860; found 317.1866.

#### *N*-(4-((3-(4-Methoxyphenyl)­Oxetan-3-yl)­Amino)­Phenyl)­Acetamide (**6a**)

Prepared according to General Procedure A using K_2_CO_3_, (36.0 mg, 0.26 mmol, 1.3 equiv), *N-*(4-aminophenyl) acetamide (36 μL, 0.24 mmol, 1.2 equiv) and PMP OSF **13** (49.2 mg, 0.2 mmol, 1.0 equiv). Purification by flash column chromatography (60% acetone/hexane) afforded aminooxetane **6a** as a white solid (58.0 mg, 93%). R_*f*_ = 0.30 (60% acetone/hexane); ^1^H NMR (400 MHz, CDCl_3_) δ 7.57–7.53 (m, 2H, 2 × Ar–CH), 7.19–7.15 (m, 2H, 2 × Ar–CH), 7.13 (br s, 1H, AcNH), 6.94–6.87 (m, 2H, 2 × Ar–CH), 6.26–6.22 (m, 2H, 2 × Ar–CH), 4.95 (d, *J* = 6.3 Hz, 2H, CHHOCHH), 4.85 (d, *J* = 6.3 Hz, 2H, CHHOCHH), 4.61 (br s, 1H, NH), 3.81 (s, 3H, OCH_3_), 2.10 (s, 3H, CH_3_). The observed characterization data (R_*f*_, ^1^H NMR) was consistent with that previously reported.^20a^

#### *N*-(4-Acetamidophenyl)-4-Methoxybenzamide (**6b**)

Prepared according to General Procedure B using *N*-(4-aminophenyl)­acetamide (82.6 mg, 0.55 mmol, 1.1 equiv). Purification by flash column chromatography (45% acetone/hexane) afforded benzamide **6b** as a white solid (74.0 mg, 72%). R_*f*_ = 0.24 (45% acetone/hexane); mp = 265–267 °C; IR (film)/cm^–1^ 3280 (NH), 1677 (C=O), 1647 (C=O), 1602, 1543, 1517, 1401, 1312, 1252, 1170, 1032, 827, 760, 670; ^1^H NMR (400 MHz, (CD_3_)_2_SO) δ 10.03 (s, 1H, NH), 9.90 (s, 1H, NH), 8.00–7.91 (m, 2H, 2 × Ar–CH), 7.71–7.62 (m, 2H, 2 × Ar–CH), 7.58–7.49 (m, 2H, 2 × Ar–CH), 7.10–7.01 (m, 2H, 2 × Ar–CH), 3.83 (s, 3H, OCH_3_), 2.03 (s, 3H, CH_3_); ^13^C NMR (101 MHz, (CD_3_)_2_SO) δ 168.0 (C=O), 164.6 (C=O), 161.8 (ArC_q_OMe), 135.1 (Ar–C_q_NH), 134.5 (Ar–C_q_NH), 129.5 (2 × Ar–CH), 127.0 (Ar–C_q_C=O), 120.8 (2 × Ar–CH), 119.2 (2 × Ar–CH), 113.6 (2 × Ar–CH), 55.4 (OCH_3_), 23.9 (CH_3_); HRMS (TOF-MS-ES^+^) *m*/*z* calcd for C_16_H_17_N_2_O_3_^+^ [M + H]^+^: 285.1234; found 285.1238.

#### 2,2-Difluoro-*N*-(3-(4-Methoxyphenyl)­Oxetan-3-yl)­Benzo­[*d*]­[1,3]­dioxol-5-Amine (**7a**)

Prepared according to General Procedure A using K_2_CO_3_, (36.0 mg, 0.26 mmol, 1.3 equiv), 2,2-difluorobenzo­[*d*]­[1,3]­dioxol-5-amine-methane (30 μL, 0.24 mmol, 1.2 equiv) and PMP OSF **13** (49.2 mg, 0.2 mmol, 1.0 equiv). Purification by flash column chromatography (40% Et_2_O/hexane) afforded aminooxetane **7a** as a white solid (66.0 mg, 98%). R_*f*_ = 0.18 (40% Et_2_O/hexane); ^1^H NMR (400 MHz, CDCl_3_) δ 7.58–7.51 (m, 2H, 2 × Ar–CH), 6.97–6.91 (m, 2H, 2 × Ar–CH), 6.78 (d, *J* = 8.6 Hz, 1H, Ar–CH), 6.04 (d, *J* = 2.3 Hz, 1H, Ar–CH), 5.94 (dd, *J* = 8.6, 2.4 Hz, 1H, Ar–CH), 4.96 (d, *J* = 6.4 Hz, 2H, CHHOCHH), 4.83 (d, *J* = 6.4 Hz, 2H, CHHOCHH), 4.63 (br s, 1H, NH), 3.83 (s, 3H, OCH_3_). The observed characterization data (R_*f*_, ^1^H NMR) was consistent with that previously reported.^20a^

#### *N*-(2,2-Difluorobenzo­[*d*]­[1,3]­dioxol-5-yl)-4-Methoxybenzamide (**7b**)

Prepared according to General Procedure B using 2,2-difluorobenzo­[*d*]­[1,3]­dioxol-5-amine (68 μL, 0.55 mmol, 1.1 equiv). Purification by flash column chromatography (45% Et_2_O/hexane) afforded benzamide **7b** as a white solid (145 mg, 90%). R_*f*_ = 0.22 (45% Et_2_O/hexane); mp = 187–191 °C; IR (film)/cm^–1^ 3358 (NH), 1647 (C=O), 1602, 1498, 1442, 1241, 1162, 1025, 810, 725; ^1^H NMR (400 MHz, (CD_3_)_2_SO) δ 7.88–7.81 (m, 2H, 2 × ArCH), 7.77–7.70 (m, 2H, Ar–CH + NH), 7.13–6.94 (m, 4H, 4 × ArCH), 3.89 (s, 3H, OCH_3_); ^13^C NMR (101 MHz, (CD_3_)_2_SO) δ 165.0 (C=O), 162.1 (Ar–C_q_OMe), 142.5 (Ar–C_q_O), 138.4 (Ar–C_q_O), 136.1 (Ar–C_q_NH), 131.3 (t, *J* = 252.4 Hz, C_q_F_2_) 129.6 (2 × Ar–CH), 126.5 (Ar–C_q_C=O), 115.8 (Ar–CH), 113.7 (2 × Ar–CH), 109.9 (Ar–CH), 103.0 (Ar–CH), 55.5 (OCH_3_); ^19^F NMR (377 MHz, (CD_3_)_2_SO) δ −49.12; HRMS (TOF-MS-ES^+^) *m*/*z* calcd for C_15_H_12_NO_4_^+^ [M + H]^+^: 308.0729; found 308.0733.

#### 6-Methoxy-*N*-(3-(4-Methoxyphenyl)­Oxetan-3-yl)­Pyridin-3-Amine (**8a**)

Prepared according to General Procedure A using K_2_CO_3_, (36.0 mg, 0.26 mmol, 1.3 equiv), 6-methoxypyridin-3-amine (29.8 mg, 0.24 mmol, 1.2 equiv) and PMP OSF **13** (49.2 mg, 0.2 mmol, 1.0 equiv). Purification by flash column chromatography (20% Et_2_O/CH_2_Cl_2_) afforded aminooxetane **8a** as a pink solid (49.4 mg, 86%). R_*f*_ = 0.28 (20% Et_2_O/CH_2_Cl_2_); mp = 74–76 °C, IR (film)/cm^–1^ 3280 (NH), 2948, 1610, 1490, 1375, 1300, 1185, 1028, 976, 816; ^1^H NMR (400 MHz, CDCl_3_) δ 7.57–7.50 (m, 2H, 2 × Ar–CH), 7.19 (d, *J* = 2.6 Hz, 1H, Ar–CH), 6.97–6.88 (m, 2H, 2 × Ar–CH), 6.74 (dd, *J* = 8.8, 3.0 Hz, 1H, Ar–CH), 6.56 (dd, *J* = 8.8, 0.4 Hz, 1H, Ar–CH), 4.98 (d, *J* = 6.4 Hz, 2H, CHHOCHH), 4.83 (d, *J* = 6.4 Hz, 2H, CHHOCHH), 4.32 (br s, 1H, NH), 3.82 (s, 6H, 2 × OCH_3_) ^13^C NMR (101 MHz, CDCl_3_) δ 159.0 (Ar–C_q_OMe), 157.7 (ArC_q_OMe), 135.4 (ArC_q_C_q_), 133.2 (Ar–CH), 131.8 (Ar–C_q_NH), 127.1 (2 × Ar–CH), 126.7 (Ar–CH), 114.2 (2 × Ar–CH), 110.9 (Ar–CH), 83.9 (CH_2_OCH_2_), 60.4 (C_q_), 55.3 (OCH_3_), 53.2 (OCH_3_); HRMS (TOF-MS-ES^+^) *m*/*z* calcd for C_16_H_19_N_2_O_3_^+^ [M + H]^+^: 287.1396; found 287.1402.

#### 4-Methoxy-N-(6-Methoxypyridin-3-yl)­Benzamide (**8b**)

Methoxybenzoyl chloride **17** (74 μL, 0.60 mmol, 1.0 equiv) was added to a solution of 6-methoxypyridin-3-amine (74.8 mg, 0.60 mmol, 1.0 equiv) and triethylamine (95 μL, 0.68 mmol, 1.03 equiv) in CH_2_Cl_2_ (1.1 mL, 0.55 M). After stirring at rt for 13 h, the reaction mixture was quenched with aq. HCl (1 M, 10 mL) and diluted with CH_2_Cl_2_ (10 mL). The layers were separated, and the aqueous layer was extracted with CH_2_Cl_2_ (3 × 10 mL). The combined organic layers were washed with brine (10 mL), dried over anhydrous Na_2_SO_4_, filtered, and concentrated in vacuo. Purification by flash column chromatography (40% EtOAc/hexane) afforded benzamide **8b** as a pink solid (110 mg, 78%). R_*f*_ = 0.24 (40% EtOAc/hexane); mp = 180–181 °C; IR (film)/cm^–1^ 3313 (NH), 2940, 1643 (C=O), 1606, 1490, 1379, 1278, 1244, 1025, 898, 820, 764, 663;^1^H NMR (400 MHz, CDCl_3_) δ 8.25 (d, *J* = 2.6 Hz, 1H, Ar–CH), 8.04 (dd, *J* = 8.9, 2.7 Hz, 1H, Ar–CH), 7.93–7.80 (m, 2H, 2 × Ar–CH), 7.63 (br s, 1H, NH), 7.07–6.91 (m, 2H, 2 × Ar–CH), 6.79 (d, *J* = 8.9 Hz, 1H, Ar–CH), 3.95 (s, 3H, OCH_3_), 3.89 (s, 3H, OCH_3_); ^13^C NMR (101 MHz, (CD_3_)_2_SO) δ 164.8 (C=O), 162.0 (Ar–C_q_OMe), 159.7 (Ar–C_q_OMe), 138.8 (Ar–CH), 132.6 (Ar–CH), 130.2 (Ar–C_q_NH), 129.5 (2 × Ar–CH), 126.4 (Ar–C_q_C=O), 113.6 (2 × Ar–CH), 109.9 (Ar–CH), 55.4 (OCH_3_), 53.2 (OCH_3_). The observed characterization data (^1^H NMR, ^13^C NMR) was consistent with that previously reported.^42^

#### 4-(3-(4-(Pyridin-2-yloxy)­Phenyl)­Oxetan-3-yl)­Morpholine (**9a**)

Amino-oxetane **14a** (47 mg, 0.2 mmol, 1.0 equiv), picolinic acid (4.9 mg, 40 μmol, 20 mol %), CuI (3.8 mg, 20 μmol, 10 mol %), and K_3_PO_4_ (93 mg, 0.44 mmol, 2.2 equiv) were added to a reaction vial. The reaction vial was sealed then evacuated and backfilled with Ar three times. 2Iodopyridine (28 μL, 0.26 mmol, 1.3 equiv) and anhydrous, degassed DMSO (0.44 mL, 0.45 M) were added sequentially by syringe to the reaction vial. The reaction mixture was heated to 110 °C and stirred for 24 h. After cooling to 25 °C, water (15 mL) followed by EtOAc (15 mL) were added. The layers were separated, and the aqueous layer was extracted with EtOAc (2 ´ 15 mL). The combined organic layers were washed with brine (15 mL), dried over anhydrous Na_2_SO_4_, filtered, and concentrated in vacuo. Purification by flash column chromatography (80–100% EtOAc/hexane) afforded amino-oxetane **9a** as a pale-yellow solid (39 mg, 60%). R_*f*_ = 0.13 (80% EtOAc/hexane);^1^H NMR (400 MHz, CDCl_3_) δ 8.22 (dd, *J* = 5.1, 2.0 Hz, 1H, Ar–CH), 7.71 (td, *J* = 7.7, 2.0 Hz, 1H, Ar–CH), 7.17 (d, *J* = 8.5 Hz, 2H, 2 × Ar–CH), 7.13–7.06 (m, 2H, 2 × Ar–CH), 7.02 (dd, *J* = 7.2, 5.0 Hz, 1H, Ar–CH), 6.94 (d, *J* = 8.3 Hz, 1H, Ar–CH), 4.93 (d, *J* = 6.0 Hz, 2H, CHHOCHH), 4.89 (d, *J* = 6.0 Hz, 2H, CHHOCHH), 3.75 (t, *J* = 4.6 Hz, 4H, 2 × OCH_2_CH_2_), 2.36 (t, *J* = 4.6 Hz, 4H, 2 × NCH_2_). The observed characterization data (R_*f*_, ^1^H NMR) was consistent with that previously reported.^20b^

#### Morpholino­(4-(Pyridin-2-yloxy)­Phenyl)­Methanone (**9b**)

Benzamide **14b** (41 mg, 0.2 mmol, 1.0 equiv), picolinic acid (4.9 mg, 40 μmol, 20 mol %), CuI (3.8 mg, 20 μmol, 10 mol %), and K_3_PO_4_ (93 mg, 0.44 mmol, 2.2 equiv) were added to a reaction vial. The reaction vial was sealed then evacuated and backfilled with Ar three times. 2-Iodopyridine (28 μL, 0.26 mmol, 1.3 equiv) and anhydrous, degassed DMSO (0.44 mL, 0.45 M) were added sequentially by syringe to the reaction vial. The reaction mixture was heated to 110 °C and stirred for 24 h. After cooling to 25 °C, water (15 mL) followed by EtOAc (15 mL) were added. The layers were separated, and the aqueous layer was extracted with EtOAc (2 ´ 15 mL). The combined organic layers were washed with brine (15 mL), dried over anhydrous Na_2_SO_4_, filtered, and concentrated in vacuo. Purification by flash column chromatography (60–80% EtOAc/hexane) afforded benzamide **9b** as a white solid (50.0 mg, 88%). R_*f*_ = 0.32 (100% EtOAc); mp = 118–120 °C; IR (film)/cm^–1^ 2963, 2919, 2853, 1629 (C=O), 1590, 1506, 1463, 1426, 1260, 1242, 1112, 1013, 884, 842, 777, 547; ^1^H NMR (400 MHz, CDCl_3_) δ 8.22 (dd, *J* = 5.1, 2.0 Hz, 1H, Ar–CH), 7.73 (ddd, *J* = 8.6, 7.2, 2.0 Hz, 1H, Ar–CH), 7.50–7.44 (m, 2H, 2 × ArCH), 7.21–7.16 (m, 2H, 2 × Ar–CH), 7.08–7.02 (m, 1H, Ar–CH), 6.96 (d, *J* = 8.2 Hz, 1H, Ar–CH), 3.71 (br, 8H, 2 × NCH_2_CH_2_); ^13^C NMR (101 MHz, CDCl_3_) δ 169.9 (C=O), 163.0 (Ar–C_q_O), 155.7 (Ar–C_q_O), 147.7 (Ar–CH), 139.7 (Ar–CH), 131.1 (Ar–C_q_C_q_), 129.1 (2 × Ar–CH), 120.8 (2 × Ar–CH), 119.1 (Ar–CH), 112.2 (Ar–CH), 66.9 (2 × NCH_2_CH_2_O); HRMS (TOF-MS-ES^+^) *m*/*z* calculated for C_16_H_17_N_2_O_3_ [M + H]: 285.1239; found 285.1246.

#### 4-(3-(4-(Phenylsulfonyl)­Phenyl)­Oxetan-3-yl)­Morpholine (**10a**)

Phenyllithium (1.82 M, 0.11 mL, 0.2 mmol, 2.0 equiv) was added dropwise to a solution of DABSO (24 mg, 0.1 mmol, 1.0 equiv) in degassed 1,4-dioxane (0.77 mL, 0.13 M). The reaction was stirred at 25 °C for 2 h. Oxetane triflate **16a** (49.0 mg, 0.13 mmol, 1.3 equiv), Pd­(OAc)_2_ (3.0 mg, 0.013 mmol, 10 mol %), Cs_2_CO_3_ (65.0 mg, 0.2 mmol, 2.0 equiv), and Xantphos (7.7 mg, 0.013 mmol, 10 mol %) were added to a separate vial. The vial was evacuated and backfilled with nitrogen three times. The 1,4-dioxane suspension was then added via syringe. After stirring at 110 °C for 16 h, the reaction was cooled to rt and CH_2_Cl_2_ (10 mL) was added. The resulting suspension was filtered through Celite and concentrated in vacuo. Purification by flash column chromatography (50% EtOAc/CH_2_Cl_2_) afforded amino-oxetane **10a** as a colorless oil (21.0 mg, 58%). R*f* = 0.24 (50% EtOAc/CH_2_Cl_2_);^1^H NMR (400 MHz, CDCl_3_) δ 8.01–7.94 (m, 4H, 4 × Ar–CH), 7.64–7.58 (m, 1H, Ar–CH), 7.58–7.51 (m, 2H, 2 × Ar–CH), 7.25–7.18 (m, 2H, 2 × Ar–CH), 4.90 (d, *J* = 6.4 Hz, 2H, CHHOCHH), 4.83 (d, *J* = 6.4 Hz, 2H, CHHOCHH), 3.72 (t, *J* = 4.5 Hz, 4H, CH_2_CH_2_OCH_2_CH_2_), 2.29 (t, *J* = 4.5 Hz, 4H, CH_2_NCH_2_). The observed characterization data (R_*f*_, ^1^H NMR) was consistent with that previously reported.^20b^

#### Morpholino­(4-(Phenylsulfonyl)­Phenyl)­Methanone (**10b**)

Amide triflate **16b** (68.0 mg, 0.2 mmol, 1.0 equiv), Pd­(OAc)_2_ (4.5 mg, 20 μmol, 10 mol %), Xantphos (12.0 mg, 20 μmol, 10 mol %), Cs_2_CO_3_ (98.0 mg, 0.3 mmol, 1.5 equiv), and sodium benzenesulfinate salt (24.0 mg, 0.2 mmol, 1.0 equiv) were added to a reaction vial. The reaction vial was sealed then evacuated and backfilled with Ar three times. Degassed 1,4-dioxane (1.12 mL, 0.18 M) was added by syringe and the reaction mixture was stirred at 110 °C for 16 h. After cooling to rt, the reaction mixture was diluted with CH_2_Cl_2_ (10 mL) and filtered through a plug of Celite, eluting through with more CH_2_Cl_2_ (3 × 10 mL). The solvent was then removed in vacuo. Purification by automated flash column chromatography (25–100% EtOAc/hexane) afforded benzamide **10b** as a white solid (11.0 mg, 15%). R*f* = 0.43 (100% EtOAc); mp = 124–126 °C; IR (film)/cm^–1^ 3061, 2965, 2919, 2855, 1631 (C=O), 1431, 1318, 1278, 1258, 1150, 1109, 1069, 1014, 917, 841, 725, 689, 598, 572, 543; ^1^H NMR (400 MHz, CDCl_3_) δ 8.03–7.98 (m, 2H, 2 × Ar–CH), 7.98–7.90 (m, 2H, 2 × Ar–CH), 7.66–7.49 (m, 5H, 5 × ArCH), 3.91–3.23 (m, 8H, 2 × NCH_2_CH_2_); ^13^C NMR (101 MHz, CDCl_3_) δ 168.5 (C=O), 142.9 (Ar–C_q_C_q_), 140.9 (Ar–C_q_SO_2_), 140.1 (Ar–C_q_SO_2_), 133.6 (Ar–CH), 129.5 (2 × Ar–CH), 128.2 (2 × Ar–CH), 127.9 (2 × Ar–CH), 127.8 (2 × Ar–CH), 66.8 (2 × OCH_2_), 48.0 (NCH_2_), 42.5 (NCH_2_); HRMS (TOF-MS-ES^+^) *m*/*z* calculated for C_19_H_21_N_2_O_4_S^+^ [M + CH_3_CNH]^+^: 373.1222; found 373.1215.

#### 4-(3-(4-(2-(Trifluoromethyl)­Pyrimidin-5-yl)­Phenyl)­Oxetan-3-yl)­Morpholine (**11a**)

Amino-oxetane triflate 16a (36.0 mg, 0.1 mmol, 1.0 equiv), Pd­(PPh_3_)_4_ (3.5 mg, 3 μmol, 3 mol %), Cs_2_CO_3_ (65.0 mg, 0.2 mmol, 2.0 equiv), and pyrimidine pinacol ester **24** (41.0 mg, 0.15 mmol, 1.5 equiv) were added to a reaction vial. The reaction vial was sealed then evacuated and backfilled with Ar three times. A degassed solution of 1,4-dioxane:water (7:3, 0.5 mL, 0.2 M) was added by syringe and the reaction mixture was stirred at 110 °C for 20 h. After cooling to 25 °C, the reaction mixture was diluted with Et_2_O (10 mL) and filtered through a plug of Celite, eluting through with more Et_2_O (3 × 10 mL). The solvent was then removed in vacuo. Purification by flash column chromatography (60–80% EtOAc/hexane) afforded amino-oxetane 11a as a white solid (35.0 mg, 93%). R_*f*_ = 0.15 (80% EtOAc/hexane); ^1^H NMR (400 MHz, CDCl_3_) δ 9.11 (s, 2H, 2 × ArCH), 7.66 (d, *J* = 8.0 Hz, 2H, 2 × ArCH), 7.29 (d, *J* = 8.0 Hz, 2H, 2 × Ar–CH), 4.96 (d, *J* = 6.2 Hz, 2H, CHHOCHH), 4.94 (d, *J* = 6.2 Hz, 2H, CHHOCHH), 3.77 (t, *J* = 4.6 Hz, 4H, CH_2_CH_2_OCH_2_CH_2_), 2.38 (d, *J* = 4.6 Hz, 4H, CH_2_NCH_2_); ^19^F NMR (377 MHz, CDCl_3_) δ −70.1. The observed characterization data (R_*f*_, ^1^H NMR, ^19^F NMR) were consistent with that previously reported.^20b^

#### Morpholino­(4-(2-(Trifluoromethyl)­Pyrimidin-5-yl)­Phenyl)­Methanone (**11b**)

Benzamide triflate **16b** (68.0 mg, 0.2 mmol, 1.0 equiv), Pd­(OAc)_2_ (2.2 mg, 10 μmol, 5 mol %), SPhos (8.4 mg, 20 μmol, 10 mol %), K_3_PO_4_ (85.0 mg, 0.4 mmol, 2.0 equiv), and pyrimidine pinacol ester **24** (82.0 mg, 0.3 mmol, 1.5 equiv) were added to a reaction vial. The reaction vial was sealed then evacuated and backfilled with Ar three times. Degassed 1,4-dioxane:water (4:1, 2 mL, 0.1 M) was added by syringe and the reaction mixture was stirred at 65 °C for 24 h. After cooling to rt, the reaction mixture was diluted with Et_2_O (10 mL) and filtered through a plug of Celite, eluting through with more Et_2_O (3 × 10 mL). The solvent was then removed in vacuo. Purification by flash column chromatography (60–80% EtOAc/hexane) afforded benzamide **11b** as a white solid (48.0 mg, 70%). R_*f*_ = 0.24 (80% EtOAc/hexane); mp = 168–170 °C; IR (film)/cm^–1^ 2967, 2921, 2857, 1628 (C=O), 1550, 1459, 1431, 1352, 1278, 1192, 1145, 1114, 1009, 842, 650, 566; ^1^H NMR (400 MHz, CDCl_3_) δ 9.10 (s, 2H, 2 × Ar–CH), 7.69 (d, *J* = 8.2 Hz, 2H, 2 × Ar–CH), 7.62 (d, *J* = 8.2 Hz, 2H, 2 × Ar–CH), 3.89–3.43 (m, 8H, 2 × NCH_2_CH_2_); ^13^C NMR (101 MHz, CDCl_3_) δ 169.2 (C=O), 155.9 (q, *J* = 36.7 Hz, Ar–C_q_CF_3_), 155.8 (2 × Ar–CH), 136.9 (Ar–C_q_C_q_), 135.3 (Ar–C_q_Ar–C_q_), 134.4 (ArC_q_Ar–C_q_), 128.5 (2 × Ar–CH), 127.6 (2 × Ar–CH), 119.7 (q, *J* = 274.9 Hz, CF_3_), 66.9 (2 × OCH_2_), 48.2 (NCH_2_), 42.6 (NCH_2_); ^19^F NMR (377 MHz, CDCl_3_) δ −70.1; HRMS (TOF-MS-ES^+^) *m*/*z* calculated for C_16_H_15_N_3_O_2_F_3_^+^ [M + H]^+^: 338.1116; found 338.1108.

#### 4-(4-(3-Morpholinooxetan-3-yl)­Phenyl)­Morpholine (**12a**)

Amino-oxetane triflate **16a** (73.0 mg, 0.2 mmol, 1.0 equiv), Pd­(OAc)_2_ (2.2 mg, 10 μmol, 5 mol %), JohnPhos (6.0 mg, 20 μmol, 10 mol %), and K_3_PO_4_ (59.0 mg, 0.3 mmol, 1.5 equiv) were added to a reaction vial. The reaction vial was sealed then evacuated and backfilled with Ar three times. Morpholine (29 μL, 0.24 mmol, 1.2 equiv) followed by anhydrous, degassed THF (0.4 mL, 0.5 M) were added by syringe and the reaction mixture was stirred at 65 °C for 24 h. After cooling to rt, the reaction mixture was diluted with Et_2_O (10 mL) and filtered through a plug of Celite, eluting through with more Et_2_O (3 × 10 mL). The solvent was then removed in vacuo. Purification by flash column chromatography (80% EtOAc/hexane) afforded amino-oxetane **12a** as a pale-yellow solid (19.0 mg, 32%). R_*f*_ = 0.20 (80% EtOAc/hexane); ^1^H NMR (400 MHz, CDCl_3_) δ 6.96 (d, *J* = 8.5 Hz, 2H, 2 × Ar–CH), 6.91 (d, *J* = 8.5 Hz, 2H, 2 × Ar–CH), 4.89 (d, *J* = 6.0 Hz, 2H, CHHOCHH), 4.86 (d, *J* = 6.0 Hz, 2H, CHHOCHH), 3.88 (t, *J* = 4.8 Hz, 4H, CH_2_CH_2_OCH_2_CH_2_), 3.73 (t, *J* = 4.6 Hz, 4H, CH_2_CH_2_OCH_2_CH_2_), 3.18 (t, *J* = 4.8 Hz, 4H, CH_2_NCH_2_), 2.31 (t, *J* = 4.6 Hz, 4H, CH_2_NCH_2_). The observed characterization data (R_*f*_, ^1^H NMR) was consistent with that previously reported.^20b^

#### 4-Morpholinonebenzene-4-Yl-Morpholine (**12b**)

Benzamide triflate **16b** (73.0 mg, 0.2 mmol, 1.0 equiv), Pd­(OAc)_2_ (2.2 mg, 10 μmol, 5 mol %), JohnPhos (6.0 mg, 20 μmol, 10 mol %), and K_3_PO_4_ (59.0 mg, 0.3 mmol, 1.5 equiv) were added to a reaction vial. The reaction vial was sealed then evacuated and backfilled with Ar three times. Morpholine (29 μL, 0.24 mmol, 1.2 equiv) followed by anhydrous, degassed THF (0.4 mL, 0.5 M) were added by syringe and the reaction mixture was stirred at 65 °C for 24 h. After cooling to rt, the reaction mixture was diluted with Et_2_O (10 mL) and filtered through a plug of Celite, eluting through with more Et_2_O (3 × 10 mL). The solvent was then removed in vacuo. Purification by flash column chromatography (80% EtOAc/hexane) afforded benzamide **12b** as a white solid (23.0 mg, 42%). R_*f*_ = 0.20 (100% EtOAc); mp = 122–124 °C; IR (film)/cm^–1^ 2960, 2915, 2893, 2852, 1627 (C=O), 1608, 1517, 1451, 1426, 1301, 1276, 1262, 1232, 1196, 1115, 1067, 1025, 928, 834, 763, 701, 643;^1^H NMR (400 MHz, CDCl_3_) δ 7.36 (d, *J* = 8.7 Hz, 2H, 2 × Ar–CH), 6.88 (d, *J* = 8.7 Hz, 2H, 2 × Ar–CH), 3.85 (t, *J* = 4.8 Hz, 4H, 2 × OCH_2_), 3.79 – 3.53 (m, 8H, 2 × NCH_2_CH_2_), 3.21 (t, *J* = 4.8 Hz, 4H, 2 × NCH_2_); ^13^C NMR (101 MHz, CDCl_3_) δ 170.6 (C=O), 152.4 (Ar–C_q_N), 129.1 (2 × Ar–CH), 125.6 (ArC_q_C_q_), 114.4 (2 × Ar–CH), 67.0 (2 × OCH_2_), 66.7 (2 × OCH_2_), 48.4 (4 × NCH_2_). The observed characterization data (mp, IR, ^1^H NMR, ^13^C NMR) was consistent with that previously reported.^43^

#### 4-(3-Morpholinooxetan-3-yl)­Phenol (**14a**)

Prepared according to General Procedure A using K_2_CO_3_, (223 mg, 1.4 mmol), morpholine (0.11 mL, 1.3 mmol) and OTIPS OSF **15** (440 mg, 1.1 mmol). Purification by flash column chromatography (80–100% EtOAc/hexane) afforded aminooxetane **14a** as a white solid (263 mg, 98%). R_*f*_ = 0.20 (80% EtOAc/hexane); ^1^H NMR (400 MHz, CDCl_3_) δ 6.93 (d, *J* = 8.1 Hz, 2H, 2 ´ Ar–CH), 6.84 (d, *J* = 8.1 Hz, 2H, 2 ´ Ar–CH), 5.09 (s, 1H, OH), 4.90 (d, *J* = 9.1 Hz, 2H, CHHOCHH), 4.89 (d, *J* = 9.1 Hz, 2H, CHHOCHH), 3.75 (t, *J* = 4.6 Hz, 4H, CH_2_CH_2_OCH_2_CH_2_), 2.33 (s, 4H, CH_2_NCH_2_). The observed characterization data (R_*f*_, ^1^H NMR) was consistent with that previously reported.^20a^

#### 4-(3-Morpholinooxetan-3-yl)­Phenyl Trifluoromethanesulfonate (**16a**)

Triflic anhydride (106 μL, 0.63 mmol, 1.1 equiv) was added dropwise to a solution of pyridine (91 μL, 1.15 mmol, 2.0 equiv) and amino-oxetane 14a (135 mg, 0.57 mmol, 1.0 equiv) in CH_2_Cl_2_ (1.15 mL, 0.5 M). After stirring at rt for 3 h, the reaction was quenched with sat. NaHCO_3_ solution (10 mL). The layers were separated, and the aqueous layer was extracted with CH_2_Cl_2_ (3 ´ 10 mL). The combined organic layers were dried over anhydrous Na_2_SO_4_, filtered, and concentrated in vacuo. Purification by flash column chromatography (80% EtOAc/hexane) afforded amino-oxetane triflate 16a as red gum (141 mg, 96%). R_*f*_ = 0.37 (80% EtOAc/hexane);^1^H NMR (400 MHz, CDCl_3_) δ 7.32 (d, *J* = 8.7 Hz, 2H, 2 × Ar–CH), 7.19 (d, *J* = 8.7 Hz, 2H, 2 × Ar–CH), 4.92 (d, *J* = 6.2 Hz, 2H, CHHOCHH), 4.87 (d, *J* = 6.2 Hz, 2H, CHHOCHH), 3.75 (t, *J* = 4.6 Hz, 4H, CH_2_CH_2_OCH_2_CH_2_), 2.32 (s, 4H, CH_2_NCH_2_). ^19^F NMR (377 MHz, CDCl_3_) δ −72.7. The observed characterization data (R_*f*_, ^1^H NMR, ^19^F NMR) were consistent with that previously reported.^20a^

#### 4-(Morpholine-4-Carbonyl)­Phenyl Trifluoromethanesulfonate (**16b**)

Triflic anhydride (0.17 mL, 1.0 mmol, 1.0 equiv) was added dropwise to a solution of triethylamine (0.17 mL, 1.2 mmol, 1.2 equiv) and benzamide **14b** (207 mg, 1.0 mmol, 1.0 equiv) in CH_2_Cl_2_ (2.5 mL, 0.25 M). After stirring at rt for 3 h, the reaction was quenched with water (30 mL). The layers were separated, and the aqueous layer was extracted with CH_2_Cl_2_ (3 ´ 15 mL). The combined organic layers were washed with sat. aq. NaHCO_3_ (30 mL), dried over anhydrous Na_2_SO_4_, filtered, and concentrated in vacuo. Purification by flash column chromatography (40–60% EtOAc/hexane) afforded benzamide triflate **16b** as an offwhite solid (257 mg, 85%). R_*f*_ = 0.41 (100% EtOAc); mp = 70–72 °C; IR (film)/cm^–1^ 2968, 2922, 2857, 1633 (C=O), 1598, 1499, 1420, 1278, 1251, 1204, 1135, 1111, 1069, 1014, 880, 846, 758, 605, 548, 517; ^1^H NMR (400 MHz, CDCl_3_) δ 7.53 (d, *J* = 8.2 Hz, 2H, 2 × Ar–CH), 7.35 (d, *J* = 8.2 Hz, 2H, 2 × Ar–CH), 3.88–3.38 (m, 8H, 2 × NCH_2_CH_2_); ^13^C NMR (101 MHz, CDCl_3_) δ 168.5 (C=O), 150.2 (Ar–C_q_OTf), 135.6 (Ar–C_q_C_q_), 129.4 (2 × Ar–CH), 121.8 (2 × Ar–CH), 118.7 (q, *J* = 321.2 Hz, C_q_F_3_), 66.8 (2 × OCH_2_), 48.2 (NCH_2_), 42.7 (NCH_2_); ^19^F NMR (377 MHz, CDCl_3_) δ −72.7. The observed characterization data (^1^H NMR, ^13^C NMR, ^19^F NMR) were consistent with that previously reported.^44^

#### 4-(4-Methoxybenzyl)­Morpholine (**18**)

Sodium triacetoxyborohydride (51.0 mg, 0.24 mmol, 1.2 equiv) was added to a solution of *p*-anisaldehyde (23 μL, 0.2 mmol, 1.0 equiv) and morpholine (21 μL, 0.24 mmol, 1.2 equiv) in anhydrous CH_2_Cl_2_ (0.67 mL, 0.3 M). After stirring at rt for 20 h, the reaction mixture was quenched with aq. NaOH (1M, 10 mL). The phases were separated, and the aqueous layer was extracted with CH_2_Cl_2_ (3 ´ 10 mL). The combined organic layers were concentrated in vacuo. The residue was acidified by additions of aq. HCl (1 M, 7 mL) and washed with Et_2_O (2 ´ 10 mL). The organic layers were discarded, and the aqueous layer was basified by addition of aq. NaOH (1M, 14 mL). The aqueous layer was extracted with CH_2_Cl_2_ (3 ´ 10 mL). The combined organic layers were dried over anhydrous Na_2_SO_4_, filtered, and concentrated in vacuo to afford benzylamine **18** as a clear colorless oil (21.0 mg, 50%). R_*f*_ = 0.20 (50% EtOAc/hexane); IR (film)/cm^–1^ 2954, 2929, 2852, 2803, 2761, 1612, 1512, 1456, 1286, 1245, 1176, 1116, 1034, 1006, 914, 866, 829, 795, 568;^1^H NMR (400 MHz, CDCl_3_) δ 7.26 (d, *J* = 8.6 Hz, 2H, 2 ´ Ar–CH), 6.87 (d, *J* = 8.6 Hz, 2H, 2 ´ Ar–CH), 3.81 (s, 3H, OCH_3_), 3.73 (t, *J* = 4.7 Hz, 4H, CH_2_OCH_2_), 3.48 (s, 2H, Ar–C_q_CH_2_), 2.46 (t, *J* = 4.7 Hz, 4H, CH_2_NCH_2_); ^13^C NMR (101 MHz, CDCl_3_) δ 158.9 (Ar–C_q_OMe), 130.5 (2 ´ Ar–CH), 129.3 (Ar–C_q_CH_2_), 113.7 (2 × Ar–CH ), 66.9 (CH_2_OCH_2_), 62.8 (CH_2_), 55.3 (OCH_3_), 53.4 (CH_2_NCH_2_). The observed characterization data (IR, ^1^H, ^13^C) were consistent with that previously reported.^45^

#### 3-(4-Chlorophenyl)-*N*-(2-Morpholinoethyl)­Oxetan-3-Amine (**20a**)

Oxetane triflate **25** (41.0 mg, 0.1 mmol, 1.0 equiv), KCl (14.9 mg, 0.2 mmol, 2.0 equiv), and KF (2.9 mg, 0.05 mmol, 0.5 equiv) were added to a reaction vial (1). The reaction vial (1) was sealed then evacuated and backfilled with Ar three times. Pd_2_(dba)_3_ (1.4 mg, 1.5 μmol, 1.5 mol %) and *t-*BuBrettPhos (2.2 mg, 4.5 μmol, 4.5 mol %) were added to a separate reaction vial (2). The reaction vial (2) was sealed then evacuated and backfilled with Ar three times. Anhydrous, degassed 1,4-dioxane (0.1 mL) was added to reaction vial (2) and the reaction mixture was stirred at 120 °C for 5 min. After cooling to 25 °C, the contents of reaction vial (2) were transferred to reaction vial (1) by syringe, and the reaction mixture was diluted with further 1,4-dioxane (0.3 mL). The reaction mixture was then heated to 130 °C and stirred for 20 h. After cooling to 25 °C, the reaction mixture was filtered through a plug of Celite and eluted with Et_2_O (10 mL). The solvent was then removed in vacuo. Purification by flash column chromatography (0–10% MeOH/CH_2_Cl_2_) afforded amino-oxetane 20a as a white crystalline solid (9.7 mg, 32%). R_*f*_ = 0.6 (30% MeOH/CH_2_Cl_2_); ^1^H NMR (400 MHz, CDCl_3_) δ 7.41–7.32 (m, 4H, 4 × Ar–CH), 4.92 (d, *J* = 6.4 Hz, 2H, CHHOCHH), 4.74 (d, *J* = 6.4 Hz, 2H, CHHOHH), 3.72 (t, *J* = 4.7 Hz, 4H, CH_2_CH_2_OCH_2_CH_2_), 2.46 (m, 4H, CH_2_CH_2_N), 2.40 (m, 4H, CH_2_NCH_2_). The observed characterization data (R_*f*_, ^1^H NMR) was consistent with that previously reported.^20b^

## Supplementary Material





## Data Availability

The data underlying this study are available in the published article, in its Supporting Information and openly available in the Imperial College London Research Data Repository at [https://doi.org/10.14469/hpc/15454].

## Notes

Calculated molecular properties, assay procedures, X-ray crystallographic data, computational data, and copies of ^1^H and ^13^C NMR spectra (PDF)
Molecular formula strings (CSV)

## Notes

Deposition numbers 2477563–2477568 contain the supplementary crystallographic data for this paper. These data can be obtained free of charge via the joint Cambridge Crystallographic Data Centre (CCDC) and Fachinformationszentrum Karlsruhe.

## Abbreviations

Ac: Acetyl
CNS: Central nervous system
CSD: Cambridge Structural Database
HHEPG: Human hepatocytes
MMP: Matched molecular pairs
MPO: Multiparameter optimization
PDHc: Pyruvate dehydrogenase
PDB: Protein Data Bank
PMP: *para*-Methoxyphenyl
RRCK: Ralph Russ Canine Kidney
SCXRD: Single-crystal X-ray diffraction
TPSA: Topological Polar Surface Area
