# Supplementary material for: Do Amino-Oxetanes Resemble Amides? A Matched Molecular Pairs Property and Structural Comparison
Source: J Med Chem. 2026 Feb 4;69(4):3941–56. doi: 10.1021/acs.jmedchem.5c02614 (PMC12951551; doi:10.1021/acs.jmedchem.5c02614)
Supplement: Supplementary file 1 [file jm5c02614_si_001.pdf]

SUPPORTING INFORMATION

Do Amino-Oxetanes Resemble Amides? A Matched Molecular Pairs Property and Structural Comparison

Hikaru Ishikura,[a] Callum S. Begg,[a] Juan J. Rojas,[a] Luka Blagojevic,[a] Gavin J. Smith,[a] Joyce Luk,[a] Rosemary A. Croft,[a] Charles Romain,[a] Chulho Choi,[b] and James A. Bull[a]*

[a] Department of Chemistry, Imperial College London, Molecular Sciences Research Hub, 82 Wood Lane W12 0BZ, UK
[b] Medicine Design, Pfizer Inc, Groton, Connecticut 06340, United States.

*E-mail: j.bull@imperial.ac.uk

Contents of SI

Additional Structures in SI S2
Synthesis of Benzylamine **S1** S3
Calculated Molecular Properties and CNS MPO S4
ADME Assay Procedures and Additional Data S8
Melting Point Data S14
CSD Searches – Torsion Angle and Hydrogen Bonding Properties S15
X-Ray Crystallography Details S18
Computational Study Details S25
Purity by Absolute Quantitative ^{1}H NMR Spectroscopy S32
References S40
^{1}H and ^{13}C NMR Spectra of Novel Compounds S41

Additional Structures in SI

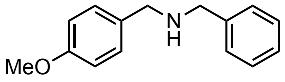

S1

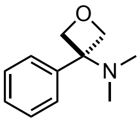

S2a

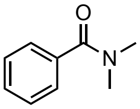

S2b

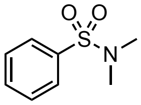

S2c

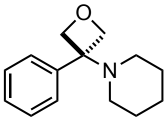

S3a

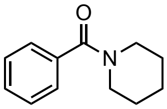

S3b

Figure S1: Structures of additional compounds in SI.

Synthesis of Benzylamines S1

***N*-Benzyl-1-(4-methoxyphenyl)methanamine (S1)**

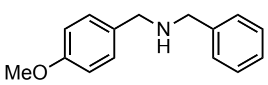

Sodium triacetoxyborohydride (51.0 mg, 0.24 mmol, 1.2 equiv) was added to a solution of *p*-anisaldehyde (23 μL, 0.2 mmol, 1.0 equiv) and benzylamine (26 μL, 0.24 mmol, 1.2 equiv) in anhydrous CH_2Cl_2 (0.67 mL, 0.3 M). After stirring at rt for 20 h, the reaction mixture was quenched with aq. NaOH (1M, 10 mL). The phases were separated, and the aqueous layer was extracted with CH_2Cl_2 (3 × 10 mL). The combined organic layers were concentrated *in vacuo*. The residue was acidified by additions of aq. HCl (1 M, 7 mL) and washed with Et_2O (2 × 10 mL). The organic layers were discarded, and the aqueous layer was basified by addition of aq. NaOH (1M, 14 mL). The aqueous layer was extracted with CH_2Cl_2 (3 × 10 mL). The combined organic layers were dried over anhydrous Na_2SO_4, filtered, and concentrated *in vacuo*. Purification by column chromatography (20% Et_2O/pentane) afforded benzylamine **S1** as a clear yellow oil (13.0 mg, 28%). R_f = 0.27 (20% Et_2O/pentane); IR (film)/cm^{-1} 3380 (NH), 3026, 2926, 2833, 1610, 1511, 1455, 1299, 1245, 1175, 1034, 814, 739, 699; ^1H NMR (400 MHz, $CDCl_3$) δ 7.37–7.26 (m, 7H, 7 × Ar-CH), 6.88 (d, *J* = 8.5 Hz, 2H, 2 × Ar-CH), 3.82 (s, 3H, OCH_3), 3.81 (s, 2H, CH_2), 3.76 (s, 2H, CH_2), 1.27 (s, 1H, NH); ^{13}C NMR (101 MHz, $CDCl_3$) δ 158.7 (Ar-C_qOMe), 140.3 (Ar-$C_q$$CH_2$), 132.4 (Ar-$C_q$$CH_2$), 129.4 (2 × Ar-CH), 128.4 (2 × Ar-CH), 128.2 (2 × Ar-CH), 127.0 (Ar-CH), 113.8 (2 × Ar-CH), 55.3 (OCH_3), 53.1 (CH_2), 52.5 (CH_2). The observed characterization data (^1H, ^{13}C) were consistent with that previously reported.[1]

Calculated Molecular Properties

Molecular properties were determined using Chemicalize developed by ChemAxon.[2] cLog*P* was calculated according to Ghose and co-workers,[3] cLog*D* (pH = 7.4) was calculated according to Csizmadia and co-workers,[4] TPSA was calculated according to Ertl and co-workers,[5] cpK_{aH} of the most basic site and cpK_a of the most acidic site was calculated according to Szegezdi and Csizmadia,[6] and CNS MPO was calculated according to Wager and co-workers.[7] (Where no basic site was present in the molecule, the contribution of the pK_a to the CNS MPO score was assumed to be 1.0).
Note: amino-oxetanes **21a** and **22a**, benzamides **20b–22b**, and sulfonamides **1c**, **3c–12c**, and **20c–22c** were not synthesized as part of this study, however, the properties were predicted to provide a comparison of calculated properties.

Table S1: Calculated molecular properties of amino-oxetanes **1a–12a**, **20a–22a**, amides **1b–12b**, **20b–22b**, and sulfonamides **1c–12c** and **20c–22c**.

Compound	Number	Property	Amino-oxetane (**a**)	Amide (**b**)	Sulfonamide (**c**)
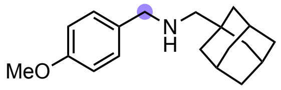	**1**	cLog*P*	3.565	3.316	3.071
		cLog*D*	2.94	3.32	3.07
		TPSA	30.49	38.33	55.4
		MW	327.47	299.19	335.46
		HBD	1	1	1
		pK_a	-	15.07	10.45
		pK_{aH}	7.91	0.19	-
		CNS MPO	4.6	4.9	5.3
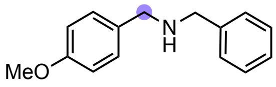	**2**	cLog*P*	2.863	2.614	2.370
		cLog*D*	2.79	2.61	2.37
		TPSA	30.49	38.33	55.40
		MW	269.34	241.29	277.34
		HBD	1	1	1
		pK_a	-	15.05	10.52
		pK_{aH}	6.67	–0.02	-
		CNS MPO	5.0	5.4	5.6
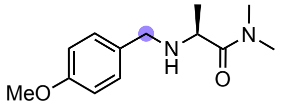	**3**	cLog*P*	0.830	0.577	0.332
		cLog*D*	0.80	0.58	0.33
		TPSA	50.80	58.64	75.71
		MW	278.35	250.3	286.35
		HBD	1	1	1
		pK_a	-	15.12	10.42
		pK_{aH}	6.22	2.30	2.30
		CNS MPO	5.8	5.8	5.8
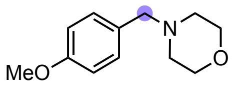	**4**	cLog*P*	1.304	0.895	0.65
		cLog*D*	1.30	0.90	0.65
		TPSA	30.93	38.77	55.84
		MW	249.31	221.26	257.30
		HBD	0	0	0
		pK_a	-	-	-
		pK_{aH}	4.98	0.35	-
		CNS MPO	5.5	5.9	6.0

Compound	Number	Property	Amino-oxetane (**a**)	Amide (**b**)	Sulfonamide (**c**)
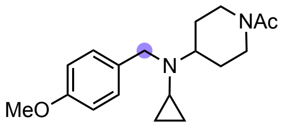	**5**	cLog*P*	1.241	0.833	0.588
		cLog*D*	1.14	0.83	0.59
		TPSA	42.01	49.85	66.92
		MW	344.46	316.40	352.45
		HBD	0	0	0
		pK_a	-	-	-
		pK_{aH}	6.84	1.57	1.49
		CNS MPO	6.0	6.0	6.0
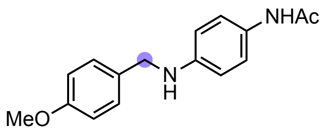	**6**	cLog*P*	2.016	2.145	1.541
		cLog*D*	2.02	2.15	1.48
		TPSA	59.59	67.43	84.5
		MW	312.37	284.32	320.36
		HBD	2	2	2
		pK_a	15.59	14.45	8.17
		pK_{aH}	3.47	-	-
		CNS MPO	5.5	5.4	5.5
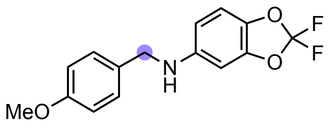	**7**	cLog*P*	3.794	3.923	3.319
		cLog*D*	3.79	3.92	3.24
		TPSA	48.95	56.79	73.86
		MW	335.307	307.25	343.3
		HBD	1	1	1
		pK_a	-	15.11	8.05
		pK_{aH}	3.64	-	-
		CNS MPO	4.5	4.4	4.7
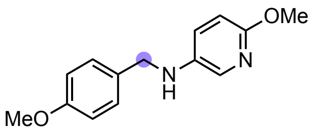	**8**	cLog*P*	1.997	2.127	1.522
		cLog*D*	2.00	2.13	1.34
		TPSA	52.61	60.45	77.52
		MW	286.33	258.28	294.33
		HBD	1	1	1
		pK_a	-	15.55	7.61
		pK_{aH}	2.64	2.04	0.89
		CNS MPO	5.8	5.8	5.8
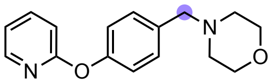	**9**	cLog*P*	2.338	1.93	1.685
		cLog*D*	2.34	1.93	1.69
		TPSA	43.82	51.66	68.73
		MW	312.37	284.32	320.36
		HBD	0	0	0
		pK_a	-	-	-
		pK_{aH}	4.89	1.96	1.94
		CNS MPO	5.8	6.0	6.0

Compound	Number	Property	Amino-oxetane (**a**)	Amide (**b**)	Sulfonamide (**c**)
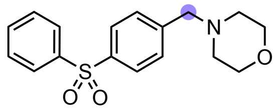	**10**	cLog*P*	2.416	2.008	1.763
		cLog*D*	2.42	2.01	1.76
		TPSA	55.84	63.68	80.75
		MW	359.44	331.39	367.43
		HBD	0	0	0
		pK_a	-	-	-
		pK_{aH}	3.87	0.41	-
		CNS MPO	5.8	6.0	5.9
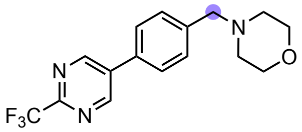	**11**	cLog*P*	2.489	2.233	2.125
		cLog*D*	2.48	2.23	2.13
		TPSA	47.48	55.32	72.39
		MW	365.36	337.30	373.35
		HBD	0	0	0
		pK_a	-	-	-
		pK_{aH}	5.46	0.38	–1.54
		CNS MPO	5.7	5.9	5.8
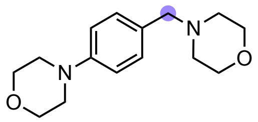	**12**	cLog*P*	1.351	0.942	0.698
		cLog*D*	1.35	0.94	0.70
		TPSA	34.17	42.01	59.08
		MW	304.39	276.34	312.38
		HBD	0	0	0
		pK_a	-	-	-
		pK_{aH}	5.41	0.46	–1.1
		CNS MPO	5.7	6.0	6.0
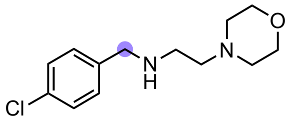	**20**	cLog*P*	1.701	1.452	1.207
		cLog*D*	1.50	1.39	1.19
		TPSA	33.73	41.57	58.64
		MW	296.8	268.74	304.79
		HBD	1	1	1
		pK_a	-	14.73	9.63
		pK_{aH}	7.18	6.62	5.83
		CNS MPO	5.5	5.8	5.8
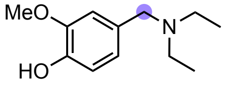	**21**	cLog*P*	1.932	1.524	1.279
		cLog*D*	1.65	1.51	1.26
		TPSA	41.93	49.77	66.84
		MW	251.33	223.27	259.32
		HBD	1	1	1
		pK_a	9.92	8.95	8.79
		pK_{aH}	7.36	0.33	-
		CNS MPO	5.8	5.8	5.4

Compound	Number	Property	Amino-oxetane (**a**)	Amide (**b**)	Sulfonamide (**c**)
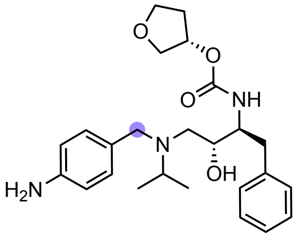	**22**	cLog*P*	3.080	2.672	2.427
		cLog*D*	2.48	2.67	2.43
		TPSA	106.28	114.12	131.19
		MW	497.64	469.58	505.63
		HBD	3	3	3
		pK_a	13.91	13.82	13.61
		pK_{aH}	3.73	3.37	2.39
		CNS MPO	3.4	3.2	3.0

For exemplar changes to N-H acidity in water. Calculated pKa values:

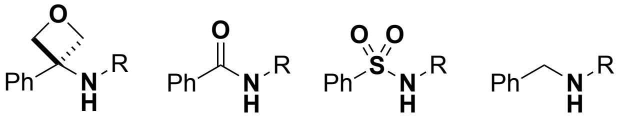

R = Me	**pK_a**	>15	**14.93**	**10.2**	>15
	**pK_{aH}**	**7.6**	-0.02	<0	**9.41**
R = Ph	**pK_a**	**15.65**	**15.92**	**7.88**	>15
	**pK_{aH}**	**3.51**	<0	<0	**4.31**
R = 2-pyridyl	**pK_a**	**15.76**	**15.10**	**6.86**	>15
	**pK_{aH}**	**6.49**	2.69	0.47	**6.59**

ADME Assay Procedures

SFlog*D* assay: Shake flask LogD (SFLogD) is the log of the octanol/water ratio measured using the shake-flask method, measured at pH 7.4.[8]

Chemical stability assay: The chemical stability assay was performed in 384 well plate format (volume = 450 µL). Plates were prefilled with 70 µL of the appropriate buffer (pH 1.2, 7.4, 10) after which the wells were spiked with 5 µL of test compounds (1 mM). Samples were incubated for their timepoint of interest (1 h, 4 h, 24 h) at 37 °C after which the samples were diluted in MeCN (and pH 12.8 buffer for experiment at pH 1.2), mixed with an internal standard and analysed by LCMS.
Aqueous buffer preparation:
pH 3: 50 µL of 1N hydrochloric acid was added to 500 mL of deionized water. The solution was titrated with 1N hydrochloric acid to pH 3, and back titrated with 1N sodium hydroxide.
pH 7.4: 100 mM sodium phosphate buffer is made by adding 56.4 g of dibasic sodium phosphate and 10.3 g of monobasic sodium phosphate to 4 L of deionized water. 250 µL of 100 mM sodium phosphate buffer was diluted with 250 µL of deionized water. The solution was titrated with 1N hydrochloric acid to pH 7.4, and back titrated with 1N sodium hydroxide.
pH 10: Fisher Scientific pH 10 buffer (SB116-500) containing potassium hydroxide, potassium carbonate, and potassium borate.
pH 12.8: 30 mL of 1N NaOH was added to 200 mL of deionized water. The solution was titrated with 1N sodium hydroxide to pH 12.8 and back titrated with 1N hydrochloric acid.

Kinetic solubility: Samples were diluted to 600 µM in in the appropriate pH buffer (pH: 3, 7.4, 10) and to 600 µM (assumed 100% solubility) in a DMSO standard. Samples were mixed thoroughly and incubated at 37 °C for 24 h. Following incubation, pH samples were transferred to a 22-micron PVDF (low binding) and filtered to remove any precipitate. The filtrate was collected and analyzed via MS/MS. The ratio of MS response (DMSO:pH) is taken and multiplied by the initial 600 µM concentration to determine the concentration (solubility) of the samples in pH buffers.

Human Hepatocyte (HHEP) assay: The high throughput HHEP stability assay was performed in a 384-well format (Greiner Bio-One, Monroe, NC). The cryopreserved HHEP were thawed, and re-suspended in Williams E medium (WEM, custom formula number 91-5233EC; Invitrogen, Grand Island, NY) supplemented with HEPES and Na_2CO_3. The cells were counted using the Trypan Blue exclusion method. A multidrop liquid dispenser (Multidrop DW, Thermo Scientific, Waltham, MA) was used to add the HHEP suspensions to the 384-well plates. The cell plates were covered and transferred to a Cytomat 2 incubator (ThermoFisher) and Biomek i7 (Beckman Coulter, Indianapolis, IN), equipped with three 3position MéCour heat exchangers. Test compounds were diluted on the Biomek i7 with buffer (15 µL) and added to the HHEP (30 µL). The final incubation contained 0.5 million cells/mL and 1 µM test compound in 45 µL total volume containing 0.01% DMSO. The incubation was carried out at 37 °C in the Cytomat 2 incubator (relative humidity ≥ 90%, 5% CO_2/95% air). At various time points (2, 4, 11, 45, 75, 120, and 240 min), the plate was pulled out from the incubator by the Biomek i7 robot and put on the MéCour heated exchanger. Samples (3 µL) were transferred to a 384-well plate containing 30 µL acetonitrile with an internal standard (IS) to quench the reaction. Water (60 µL) was added to the quenched plate to dilute the organic solvent content for LC-MS/MS analysis. The plates were centrifuged at 3000 rpm for 10 min at 4 °C, sealed and subsequently analyzed using LC-MS/MS without further transfer of the supernatant. Propranolol (CYP2D6), midazolam (CYP3A) and triazolam (CYP3A, low clearance), naloxone (UGT2B7), carbazeran (AO), and verapamil (CYP3A, CYP2C8) were used as positive controls. For CYP inhibition study, 1 mM ABT (a pan-cYP inhibitor) was pre-incubated with HHEP for 30 minutes at 37 °C before adding test compounds. Values are n >3 unless otherwise noted.

Permeability assay (RRCK): Ralph Russ canine kidney cells were used for permeability measurements using a cell monolayer in 96-transwell format. The detailed assay conditions have been reported previously.[9] Test compounds were added to the donor wells and a buffer was added to the receiver wells to start the apical to basolateral transport assay. Time 0 and 1.5 h incubation samples were taken for LC-MS/MS analysis. Apparent permeability (P_{app}) was calculated using the equation below (1), where Area is the surface area of the cell monolayer (0.0625 cm^2), $C_D(0)$ is the

concentration in the donor at time 0, t is time in seconds, M_r is the mass of compound appearing in the receiver as a function of time, and dM_r/dt is flux of the compound across the cell monolayer.

$$P_{app} = \frac{1}{Area \times C_D(0)} \times \frac{dM_r}{dt} \cdots (1)$$

Acid dissociation constant (pKa) calculator: ACD/pK_a predictor (Percepta version 2021.2.2) was used to calculate accurate acid-base ionization constants (pK_a values) at 25 °C and zero ionic strength in aqueous solutions. The accuracy of calculations is usually better than ±0.2 pK_a units except for very complex structures or poorly characterized substituents, where the accuracy is usually better than ±0.5 pK_a units. By default, pK_a was computed in the range of 1–13.

Table S2: cpK_a and pK_a of compounds **4a**, **10a**, **17**, **S1**.

Compound	Number	cpK_a	pK_a
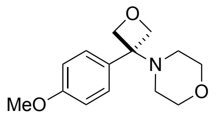	**4a**	5.44	4.61
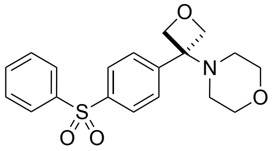	**10a**	4.65	3.08
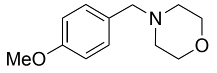	**17**	6.68	7.25
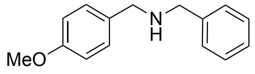	**S1**	8.78	8.81

cSFlog*D* calculator: ACD/SFlog*D* predictor (Percepta version 2021.2.2) trained with an internal Pfizer dataset was used to calculate the SFlog*D* values at pH 7.4.

Table S3: SFlogD (pH = 7.4) and RRCK permeability of compounds **1–12**.

Compound	Number	cSFlog*D*	SFlog*D*[a]	RRCK permeability (P_{app}, × 10^{-6} cm s^{-1})[a]
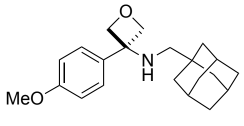	**1a**	3.37	3.595	25.786[b]
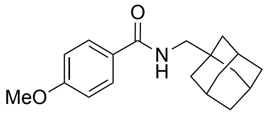	**1b**	3.74	3.831	32.710[b]
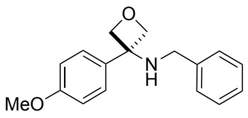	**2a**	2.53	2.489	36.506
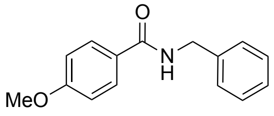	**2b**	2.61	2.737	38.051 [b]
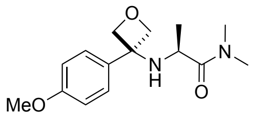	**3a**	0.75	0.418	27.329[b]
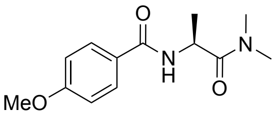	**3b**	0.95	1.003	32.187[b]
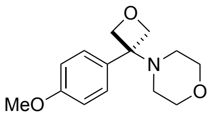	**4a**	1.93	1.171	39.173
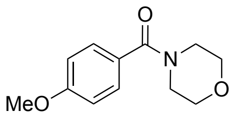	**4b**	0.78	0.823	48.306[b]
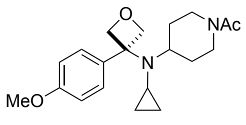	**5a**	1.89	1.678	38.754[b]
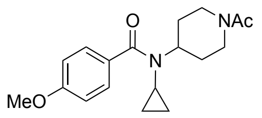	**5b**	1.21	1.246	31.720[b]

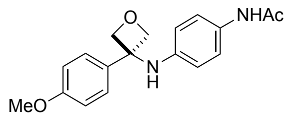	**6a**	2.40	ND	ND
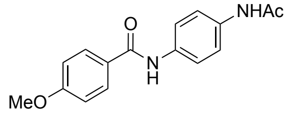	**6b**	2.04	1.917	14.035[b]
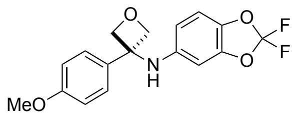	**7a**	3.98	ND	ND
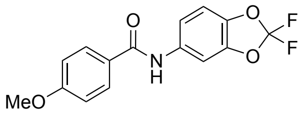	**7b**	3.76	3.815	28.269[b]
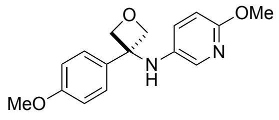	**8a**	2.50	2.535	37.199[b]
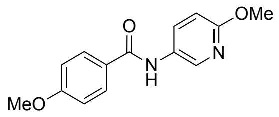	**8b**	2.80	2.488	20.930[b]
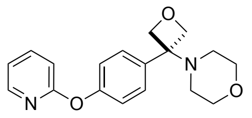	**9a**	2.27	1.324	43.590
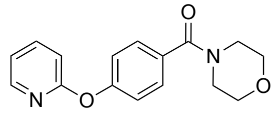	**9b**	1.45	1.079	47.203
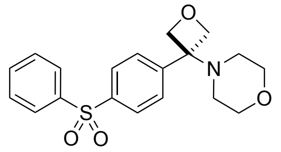	**10a**	2.25	1.552	36.723
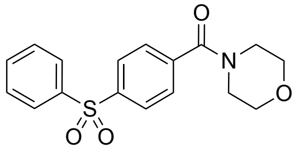	**10b**	1.24	1.110	37.772
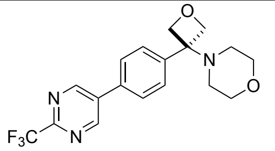	**11a**	2.85	1.660	<36.073

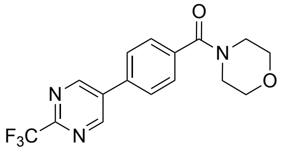	**11b**	2.19	1.311	43.211
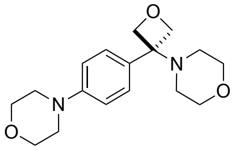	**12a**	1.23	0.331	39.335
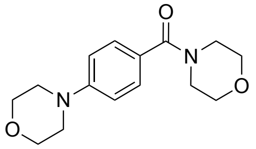	**12b**	0.84	0.444	36.418

[a]Average of 2 runs unless otherwise stated. [b]Data from only one replicate.
Note compounds 6a and 7a were not detected in the assay read out.

Melting Point Data for Selected Compounds

Table S4: Melting point data for amino-oxetanes **1a**–**12a** and benzamides **1b**–**12b.**

Compound	Number	m.p. Amino-oxetane (**a**)	m.p. Amide (**b**)
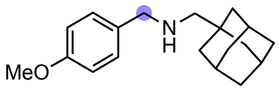	**1**	Oil	195–196
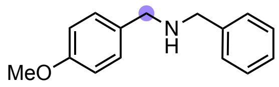	**2**	77–79	134–136
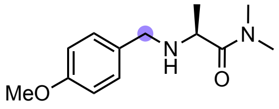	**3**	Oil	Paste
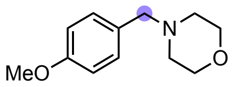	**4**	98–100	Paste
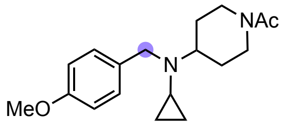	**5**	154–156	104–105
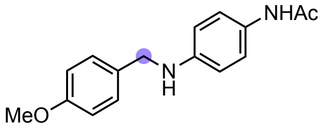	**6**	100–103	265–267
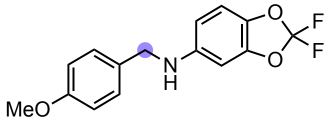	**7**	116–118	187–191
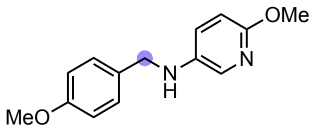	**8**	74–76	180–181
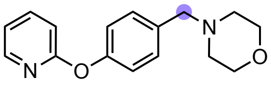	**9**	Decomp. at 200	118–120
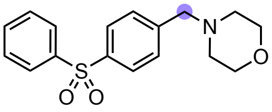	**10**	Oil	124–126
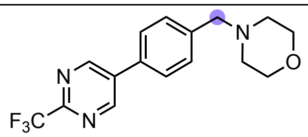	**11**	160–162	168–170
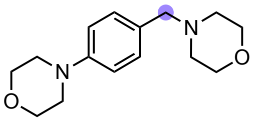	**12**	174–176	122–124

CSD Searches – Torsion Angle and Hydrogen Bonding Properties

CSD version 5.44 (April 2023) and 5.44 updates (June 2023) was used for the searches. Queries were generated using *Conquest* Version 2023 1.0 (Build 376230). The search parameters dictated that the crystal structures must be organic, non-polymeric, single crystal structures only, R1 ≤ 0.10, no errors and no disorder allowed.

Torsion Angles

The draw tool was used to search for 3,3'-substituted amino-oxetane, amide, sulfonamide, and α-trifluoroethyl amine containing compounds. The following structural representations were used to identify the torsion angles in hit compounds, with the atoms selected in the denoted order to measure the torsion angles:

3,3'-Amino-oxetanes | Amides | Sulfonamides | α-CF_3 amines

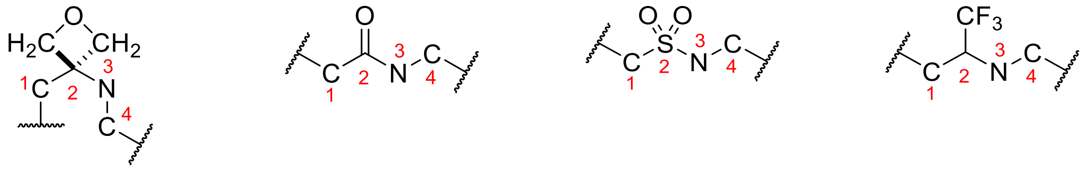

46 torsion angles identified | 169859 torsion angles identified | 43111 torsion angles identified | 1750 torsion angles identified

The Hitlists were then exported to *Mercury* and the data saved as a .csv file for processing in *Excel*. Histograms of the torsion angles were generated in *Excel* with the y-axis normalized as a percentage to allow comparison across the functionalities.

Hydrogen Bonding

The draw tool was used to search for 3,3'-amino-oxetane and amide containing compounds that accepted a hydrogen bond to the oxygen atom in the functionality. The following structural representations (X = any atom) were used to identify the hydrogen bonds in the hit compounds, with the atoms selected in the denoted order to measure the bond angles and the distance of the hydrogen bond measured if it was less than the Van der Waals radius:

3,3'-Amino-oxetanes | Amides | Sulfonamides

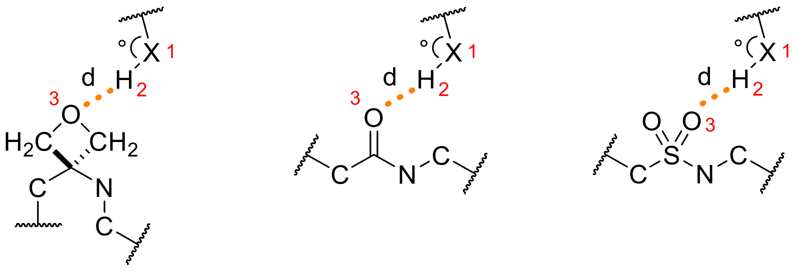

79 hydrogen bonds identified | 229802 hydrogen bonds identified | 12316 hydrogen bonds identified

The Hitlists were then exported to *Mercury* and the data saved as a .csv file for processing in *Excel*. Histograms of the torsion angles were generated in *Excel* with the y-axis normalized as a percentage to allow comparison across the functionalities.

The same parameters were utilized to evaluate the NH hydrogen bond donation propensity of 3,3'-amino-oxetanes. However, the CSD search yielded only 12 instances of NH hydrogen bonding (less that Van der Waals radius) within the Hitlist, an insufficient sample size for robust statistical analysis.

A. Torsion angles of amino-oxetane 2a, benzamide 2b, and analogues

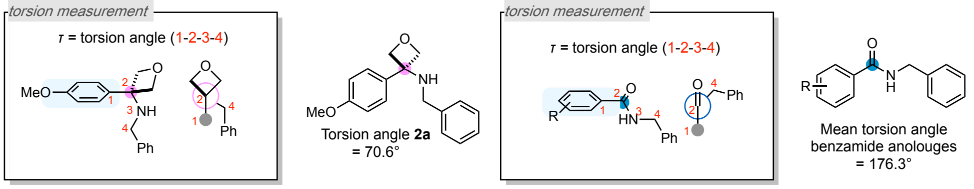

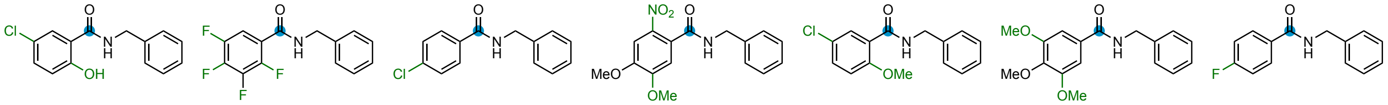

BATXOL: τ = 177.2° **CEDQIN**: τ = 178.6° **DULGOI**: τ = 173.7° **GEVLEZ**: τ = 167.6° **ZAVTAU**: τ = 179.3° **SUJFUA**: τ = 172.2° **SUSFUI**: τ = 176.7°

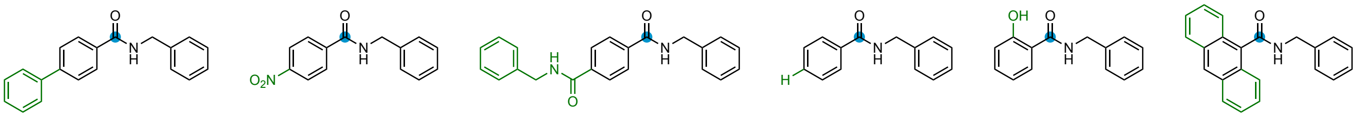

TAHYAD: τ = 175.0° **TAHYEH**: τ = 179.4° **UMOPAO**: τ = 179.5° **XEQNEP**: τ = 177.8° **XIZCEP**: τ = 179.0° **KEYJAD** (Z`=4): = 175.8°, 179.6°, 177.6° and 172.0°

B. Torsion angles of amino-oxetanes

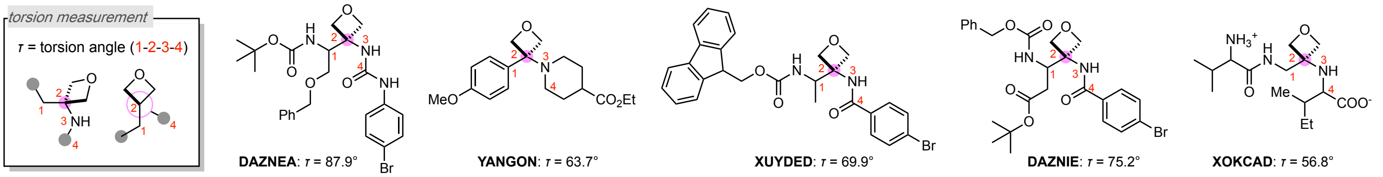

DAZNEA: τ = 87.9° **YANGON**: τ = 63.7° **XUYDED**: τ = 69.9° **DAZNIE**: τ = 75.2° **XOKCAD**: τ = 56.8°

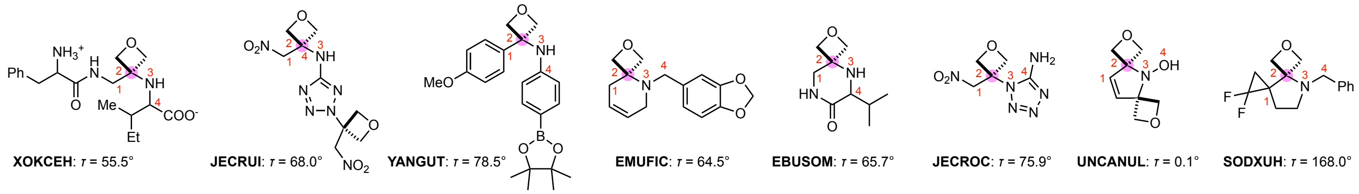

XOKCEH: τ = 55.5° **JECRUI**: τ = 68.0° **YANGUT**: τ = 78.5° **EMUFIC**: τ = 64.5° **EBUSOM**: τ = 65.7° **JECROC**: τ = 75.9° **UNCANUL**: τ = 0.1° **SODXUH**: τ = 168.0°

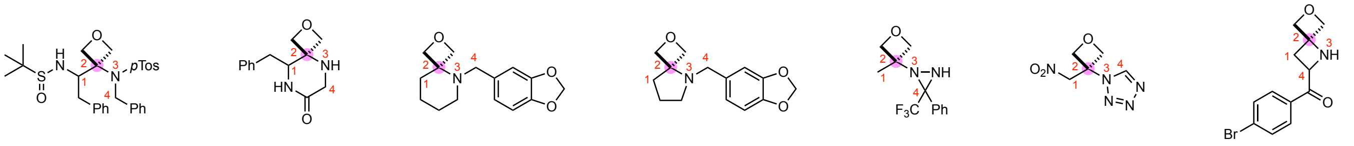

DAZNOK: τ = 66.0° **EBUSIG**: τ = 63.5° **SOBYEO**: τ = 68.6° **SOBYIS**: τ = 170.4° **UJERAF**: τ = 95.2° **JECSOD**: τ = 105.5° **VAFWIK**: τ = 169.1°

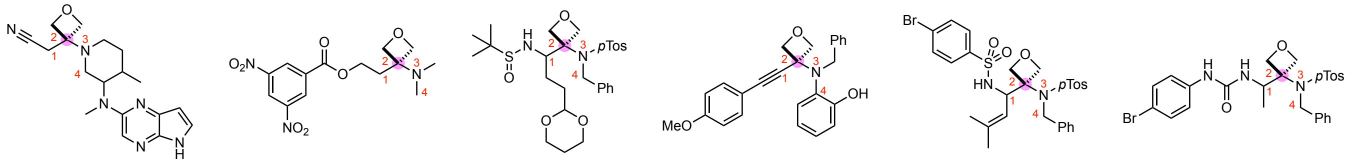

LOFBUF: τ = 64.6° **EMUDIA**: τ = 67.6° **DAZNUQ**: τ = 88.9° **BUKWUB**: τ = 77.1° **DAZNAW**: τ = 69.7° **DAZMUP**: τ = 83.4°

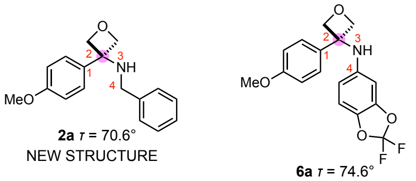

2a τ = 70.6°
NEW STRUCTURE

6a τ = 74.6°
NEW STRUCTURE

Additional structures from ref. 2 (not included in the search parameters due to the version of CSD used):

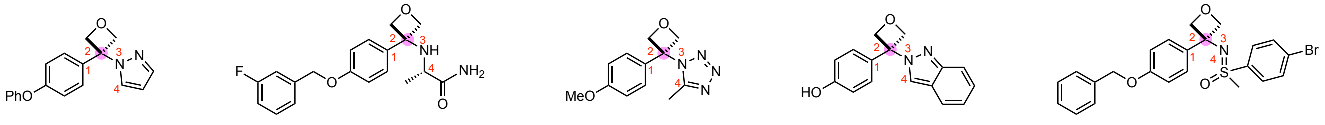

MUNTAU: τ = 86.1° **MUNTEY** (Z`=2): τ = 66.7° and 52.4° **MUNTIC**: τ = 94.3° **MUNTOI**: τ = 81.4° **MUNWIF**: τ = 67.3°

Figure S2: A. Compounds from the CSD relevant to the comparison of torsion angles for amino-oxetane and benzamide pair **2**. B. Torsion angles of amino-oxetanes in the CSD.

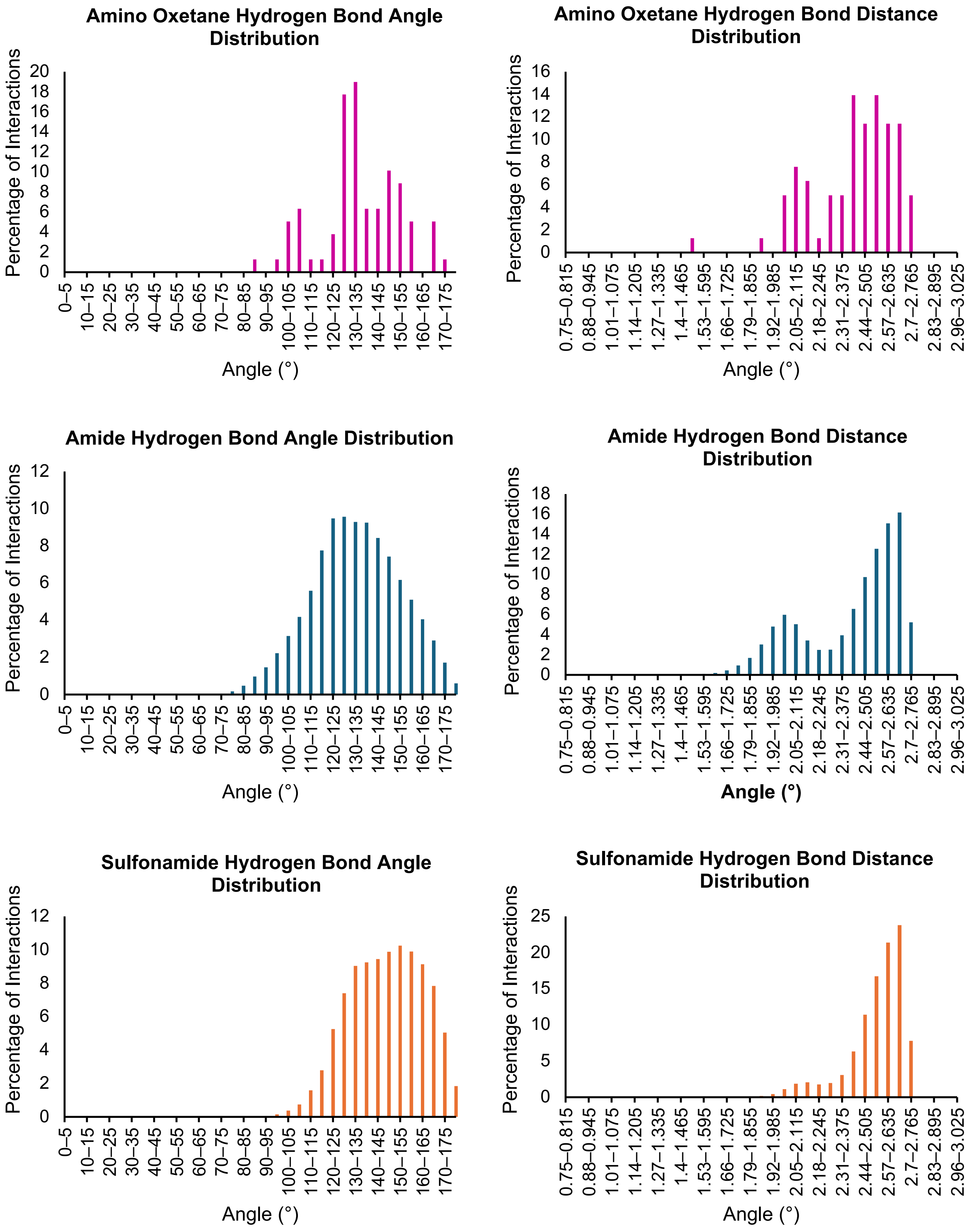

Figure S3: Histograms indicating the hydrogen bond angles and distances for amino-oxetanes, benzamides, and sulfonamides in the CSD.

X-Ray Crystallography Details

Crystals suitable for X-ray analysis were grown by slow evaporation from acetone at 25 °C.

Data were collected using Agilent Xcalibur PX Ultra A [**2a**, **6a**, **2c**, **20a**] and Agilent Xcalibur 3 E [**2b**, **6b**] diffractometers, and the structures were solved by direct methods and refined by full-matrix least squares on F2 for all data using the OLEX2,[10] SHELXTL,[11] and SHELX-2013[12] program systems.[13] Crystallographic data and related CIFs for the structures **2a**, **2b**, **6a**, **6b**, **2c** and **20a** have been deposited with the joint Cambridge Crystallographic Data Centre and Fachinformationszentrum Karlsruhe Access Structures service and are available free of charge with the following deposition numbers: CCDC Deposition Numbers: 2477563-2477568.

X-Ray Crystallography Notes

The N–H hydrogen atoms in the structures of **2a**, **2b**, **6a**, **6b**, **2c** and **20a** were located from a Δ*F* map and refined freely subject to an N–H distance constraint of 0.90 Å.

2b was attempted to be solved as a twin but the R_{int} was significantly more reliable when the data was refined as a single crystal.

The structure of **20a** was found to contain two crystallographically independent molecules (**20a** and **20a**-B) in the asymmetric unit.

Crystal Structure: 2a

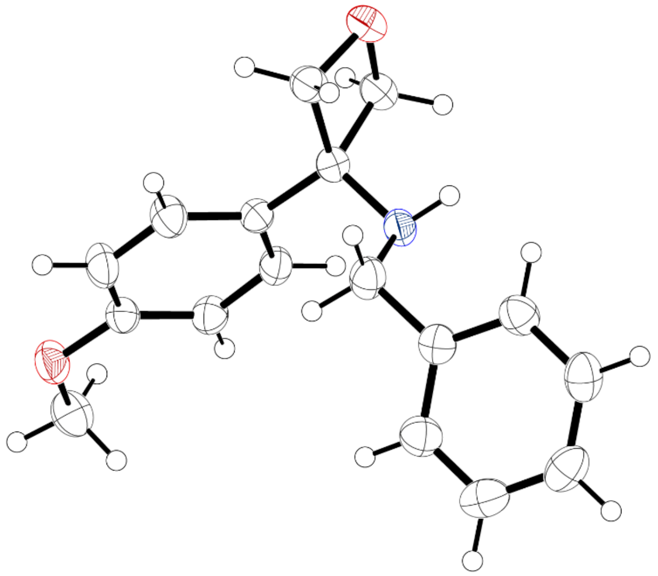

Figure S4: The crystal structure of **2a** (50% probability ellipsoids).

CCDC deposition number	2477563
Empirical formula	$C_{17}H_{19}NO_2$
Formula weight	269.33
Temperature/K	174(2)
Crystal system	triclinic
Space group	*P*-1
a/Å	8.0448(4)
b/Å	8.8727(4)
c/Å	10.9231(5)
α/°	76.400(4)
β/°	89.496(4)
γ/°	69.032(4)
Volume/Å^3	705.19(6)
Z	2
ρ_{calc}g/cm^3	1.268
μ/mm^{-1}	0.658
F(000)	288.0
Crystal size/mm^3	0.29 × 0.26 × 0.21
Radiation	Cu Kα (λ = 1.54184)
2Θ range for data collection/°	8.358 to 147.388
Index ranges	-10 ≤ h ≤ 9, -10 ≤ k ≤ 10, -13 ≤ l ≤ 13
Reflections collected	10920
Independent reflections	2779 [R_{int} = 0.0325, R_{sigma} = 0.0236]
Data/restraints/parameters	2779/1/186
Goodness-of-fit on F^2	1.055
Final R indexes [I>=2σ (I)]	R_1 = 0.0421, wR_2 = 0.1048
Final R indexes [all data]	R_1 = 0.0524, wR_2 = 0.1137
Largest diff. peak/hole / e Å^{-3}	0.19/-0.21

Crystal Structure: 2b

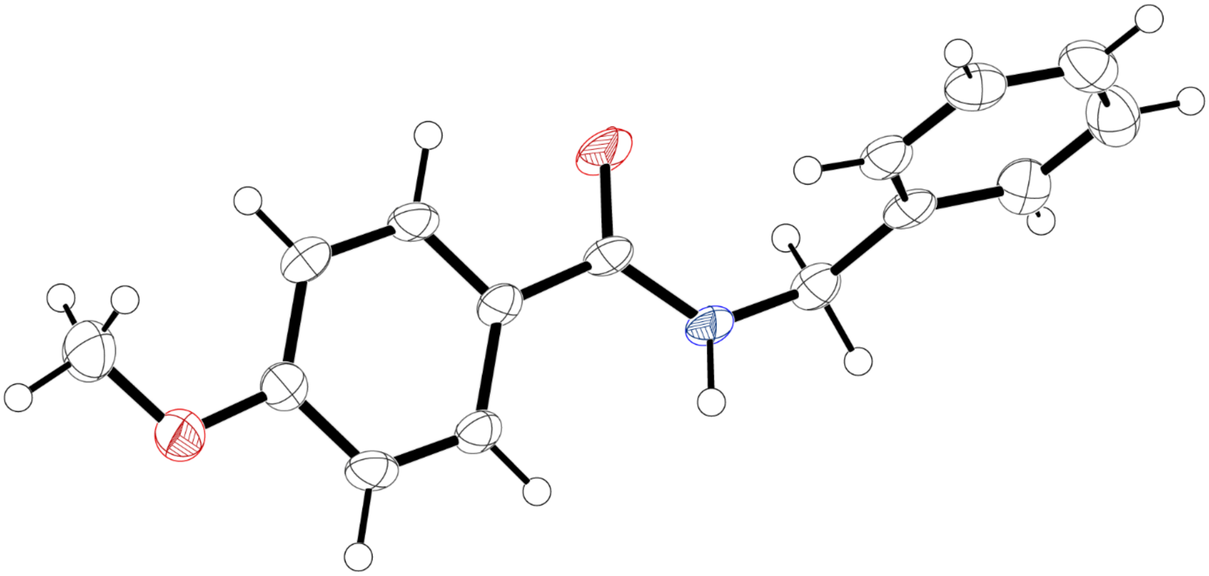

Figure S5: The crystal structure of **2b** (50% probability ellipsoids).

CCDC deposition number	2477564
Empirical formula	$C_{15}H_{15}NO_2$
Formula weight	241.28
Temperature/K	173.00(14)
Crystal system	monoclinic
Space group	$P2_1/n$
a/Å	5.8100(3)
b/Å	28.0135(16)
c/Å	7.7795(4)
α/°	90
β/°	91.451(6)
γ/°	90
Volume/Å^3	1265.77(12)
Z	4
ρ_{calc}g/cm^3	1.266
μ/mm^{-1}	0.084
F(000)	512.0
Crystal size/mm^3	0.287 × 0.215 × 0.148
Radiation	Mo Kα (λ = 0.71073)
2Θ range for data collection/°	5.436 to 56.324
Index ranges	-7 ≤ h ≤ 7, -33 ≤ k ≤ 35, -6 ≤ l ≤ 10
Reflections collected	7842
Independent reflections	2625 [R_{int} = 0.0479, R_{sigma} = 0.0530]
Data/restraints/parameters	2625/1/168
Goodness-of-fit on F^2	1.087
Final R indexes [I>=2σ (I)]	R_1 = 0.0644, wR_2 = 0.1483
Final R indexes [all data]	R_1 = 0.0906, wR_2 = 0.1654
Largest diff. peak/hole / e Å^{-3}	0.32/-0.23

Crystal Structure: 6a

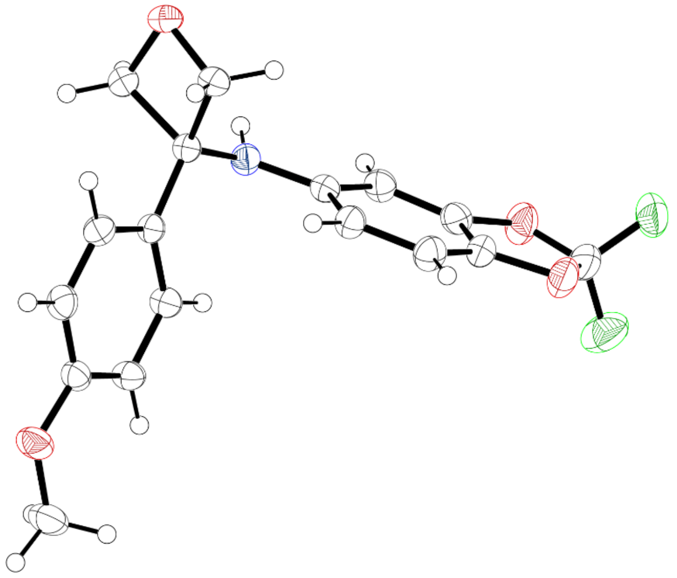

Figure S6: The crystal structure of **6a** (50% probability ellipsoids).

CCDC deposition number	2477565
Empirical formula	$C_{17}H_{15}F_2NO_4$
Formula weight	335.30
Temperature/K	173.1(6)
Crystal system	monoclinic
Space group	$P2_1/c$
a/Å	10.6381(2)
b/Å	5.24920(10)
c/Å	27.5688(5)
α/°	90
β/°	99.940(2)
γ/°	90
Volume/Å^3	1516.37(5)
Z	4
ρ_{calc}g/cm^3	1.469
μ/mm^{-1}	1.035
F(000)	696.0
Crystal size/mm^3	0.32 × 0.13 × 0.06
Radiation	Cu Kα (λ = 1.54184)
2Θ range for data collection/°	6.51 to 147.184
Index ranges	-12 ≤ h ≤ 13, -6 ≤ k ≤ 6, -34 ≤ l ≤ 32
Reflections collected	11383
Independent reflections	3036 [R_{int} = 0.0311, R_{sigma} = 0.0293]
Data/restraints/parameters	3036/1/222
Goodness-of-fit on F^2	1.054
Final R indexes [I>=2σ (I)]	R_1 = 0.0365, wR_2 = 0.0891
Final R indexes [all data]	R_1 = 0.0445, wR_2 = 0.0956
Largest diff. peak/hole / e Å^{-3}	0.18/-0.19

Crystal Structure: 6b

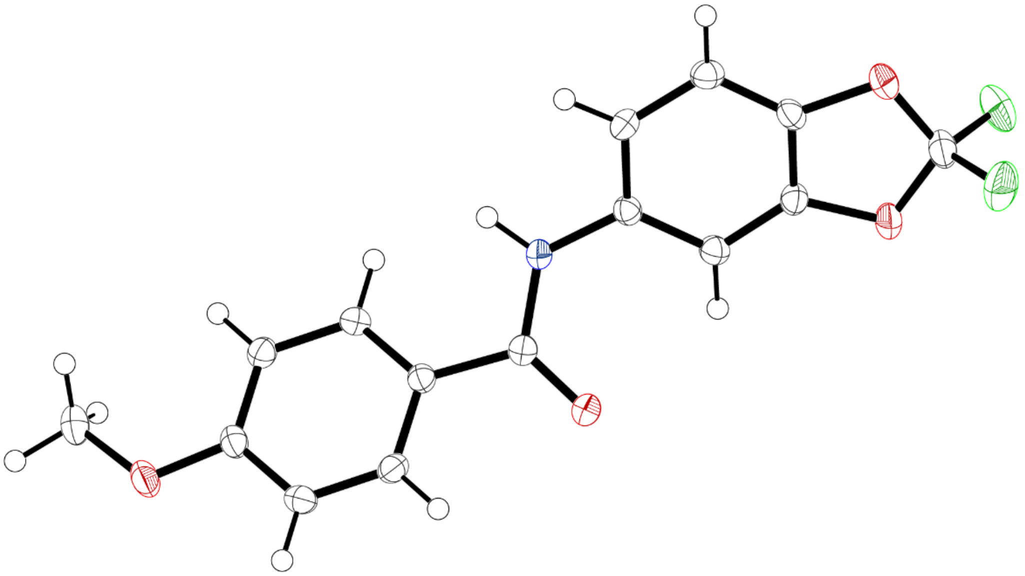

Figure S7: The crystal structure of **6b** (50% probability ellipsoids).

CCDC deposition number	2477566
Empirical formula	$C_{15}H_{11}F_2NO_4$
Formula weight	307.25
Temperature/K	172.95(10)
Crystal system	triclinic
Space group	*P*-1
a/Å	5.4268(4)
b/Å	7.6145(5)
c/Å	15.5073(10)
α/°	93.085(5)
β/°	97.996(5)
γ/°	90.359(5)
Volume/Å^3	633.59(8)
Z	2
ρ_{calc}g/cm^3	1.611
μ/mm^{-1}	0.136
F(000)	316.0
Crystal size/mm^3	0.6 × 0.2 × 0.07
Radiation	Mo Kα (λ = 0.71073)
2Θ range for data collection/°	5.314 to 57.75
Index ranges	-6 ≤ h ≤ 7, -10 ≤ k ≤ 9, -20 ≤ l ≤ 20
Reflections collected	8458
Independent reflections	2822 [R_{int} = 0.0256, R_{sigma} = 0.0207]
Data/restraints/parameters	2822/1/204
Goodness-of-fit on F^2	1.026
Final R indexes [I>=2σ (I)]	R_1 = 0.0345, wR_2 = 0.0893
Final R indexes [all data]	R_1 = 0.0405, wR_2 = 0.0940
Largest diff. peak/hole / e Å^{-3}	0.28/-0.25

Crystal Structure: 2c

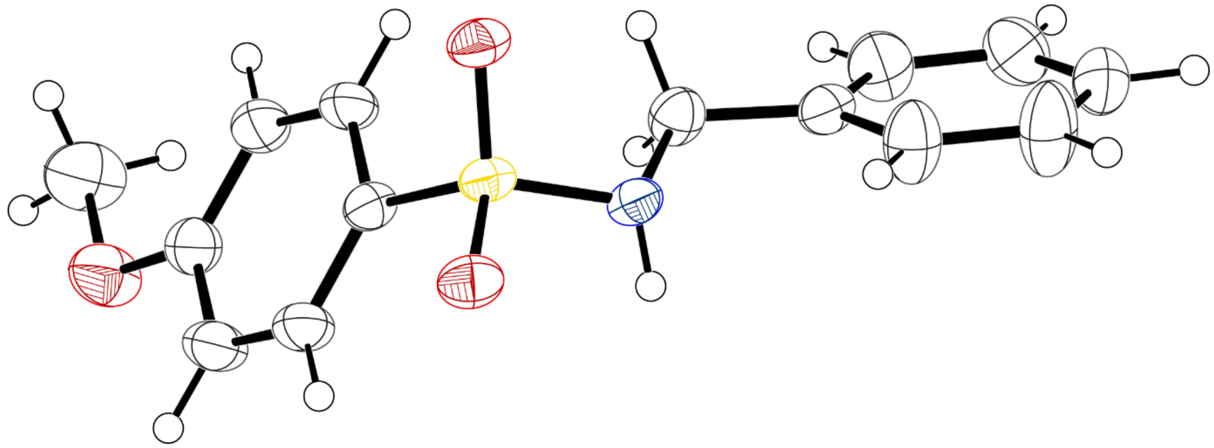

Figure S8: The crystal structure of **2c** (50% probability ellipsoids).

CCDC deposition number	2477567
Empirical formula	$C_{14}H_{15}NO_3S$
Formula weight	277.33
Temperature/K	173(2)
Crystal system	monoclinic
Space group	$P2_1/c$
a/Å	9.1985(2)
b/Å	5.68690(10)
c/Å	25.4255(6)
α/°	90
β/°	93.431(2)
γ/°	90
Volume/Å^3	1327.65(5)
Z	4
ρ_{calc}g/cm^3	1.387
μ/mm^{-1}	2.206
F(000)	584.0
Crystal size/mm^3	0.27 × 0.22 × 0.11
Radiation	Cu Kα (λ = 1.54184)
2Θ range for data collection/°	6.966 to 147.536
Index ranges	-11 ≤ h ≤ 11, -7 ≤ k ≤ 6, -30 ≤ l ≤ 31
Reflections collected	20214
Independent reflections	2660 [R_{int} = 0.0437, R_{sigma} = 0.0200]
Data/restraints/parameters	2660/1/177
Goodness-of-fit on F^2	1.024
Final R indexes [I>=2σ (I)]	R_1 = 0.0372, wR_2 = 0.0979
Final R indexes [all data]	R_1 = 0.0427, wR_2 = 0.1020
Largest diff. peak/hole / e Å^{-3}	0.27/-0.32

Crystal Structure: 20a

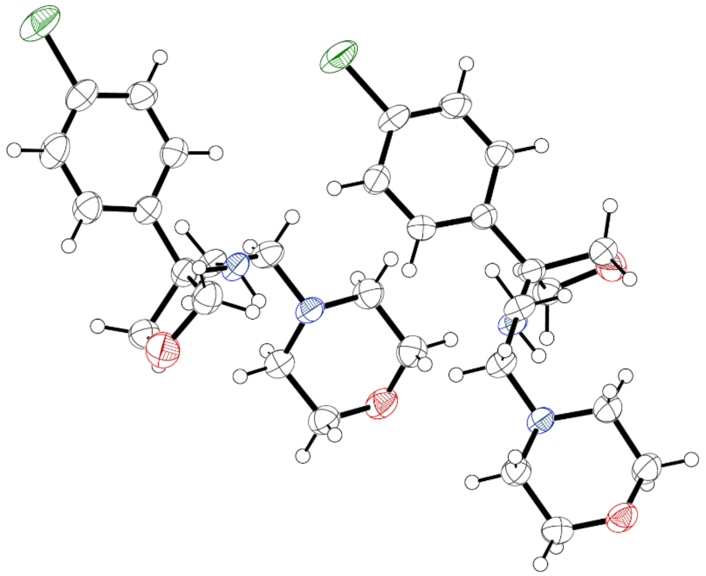

Figure S9: The crystal structure of **20a** (50% probability ellipsoids).

CCDC deposition number	2477568
Empirical formula	$C_{15}H_{21}ClN_2O_2$
Formula weight	296.79
Temperature/K	173(2)
Crystal system	triclinic
Space group	*P*-1
a/Å	9.0670(5)
b/Å	10.8325(5)
c/Å	16.2404(8)
α/°	75.416(4)
β/°	81.531(4)
γ/°	77.689(4)
Volume/Å^3	1500.81(14)
Z	4
ρ_{calc}g/cm^3	1.313
μ/mm^{-1}	2.280
F(000)	632.0
Crystal size/mm^3	0.2 × 0.11 × 0.05
Radiation	Cu Kα (λ = 1.54184)
2Θ range for data collection/°	8.582 to 147.298
Index ranges	-11 ≤ h ≤ 11, -13 ≤ k ≤ 13, -20 ≤ l ≤ 20
Reflections collected	21690
Independent reflections	5906 [R_{int} = 0.0503, R_{sigma} = 0.0453]
Data/restraints/parameters	5906/2/369
Goodness-of-fit on F^2	1.012
Final R indexes [I>=2σ (I)]	R_1 = 0.0457, wR_2 = 0.1026
Final R indexes [all data]	R_1 = 0.0799, wR_2 = 0.1210
Largest diff. peak/hole / e Å^{-3}	0.22/-0.28

Computational Study Details

General considerations:

DFT calculations were performed using Gaussian 16 (revision C.01).[14] Unless stated otherwise, calculations were performed at B3LYP/6-311G+(d,p)-GD3BJ, SMD=water level of theory. All calculations were performed without restriction on symmetry at 298.15K (default), unless stated otherwise (e.g. constrained dihedral angles for "bound drug molecules, see details in distortion analysis). All transition states were characterized by normal coordinate analysis revealing precisely one imaginary mode corresponding to the intended reaction. Gibbs free energies (ΔG) were calculated at 298.15 K.

Initial structures of molecule bound to its respective drug target taken from the Protein Data Bank in Europe (PDBe), i.e. Ethamivan: PDBe – 2xdl and Amprenavir: PDBe - 3nu3. The investigated dihedral angle was frozen, and the rest of the structure optimized freely.

Natural Bond Orbital (NBO) analysis was carried out using the NBO 7 program.[15]

Conformational analyses were performed with CREST 2.12 and the conformer ensembles were sorted and refined using CENSO 2.1.2.[16]

Full coordinates for all the stationary points (e.g. Gaussian log files), conformer ensembles and output files from CREST and CENSO are available in the Imperial College London Data repository here: 10.14469/hpc/15454

Examination of the C-N bond rotation:

360° relaxed scans of the C-N dihedral angle for each of the relevant structures was performed. Examples for amino-oxetane **S6a**, *N*,*N*-dimethylbenzamide **S6b**, and sulfonamide **S6c** are provided in Figures S10–S12. Molecular geometries from relevant minima and maxima (i.e. TS) were extracted and re-optimized at B3LYP/6311G+dp-GD3BJ, SMD=water level of theory (maxima were subject to transition state optimization). Gibbs free energies of the TS and minima (ΔG at 298K) were calculated and plotted against the relevant dihedral angle to create the full rotational profiles (Figures S13–S20).

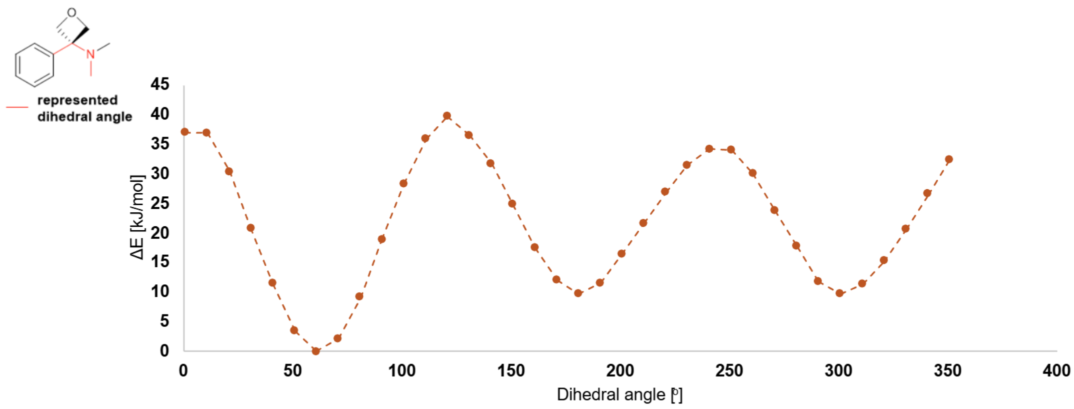

Figure S10: 360º scan of dihedral angle in amino-oxetane **S6a**. Minima used as starting geometries for stable minima optimization, maxima used as starting geometries for TS optimization.

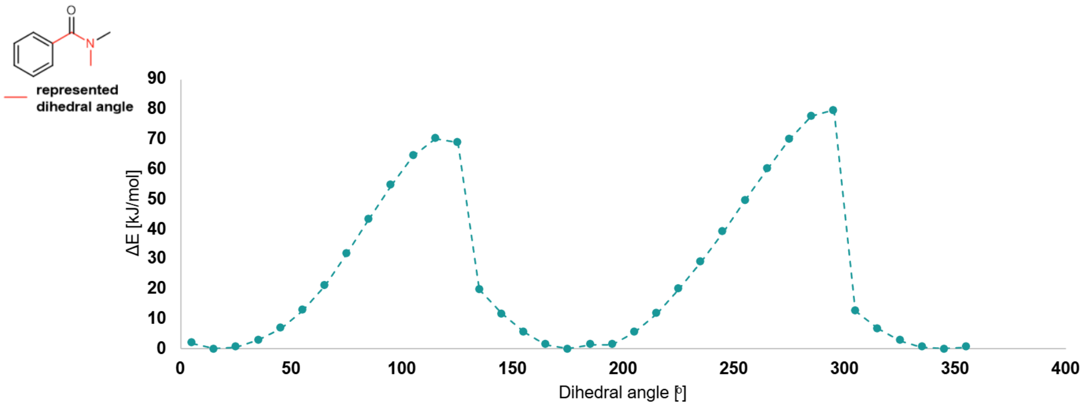

Figure S11: 360º scan of dihedral angle in benzamide **S6b**. Minima used as starting geometries for stable minima optimization, maxima used as starting geometries for TS optimization.

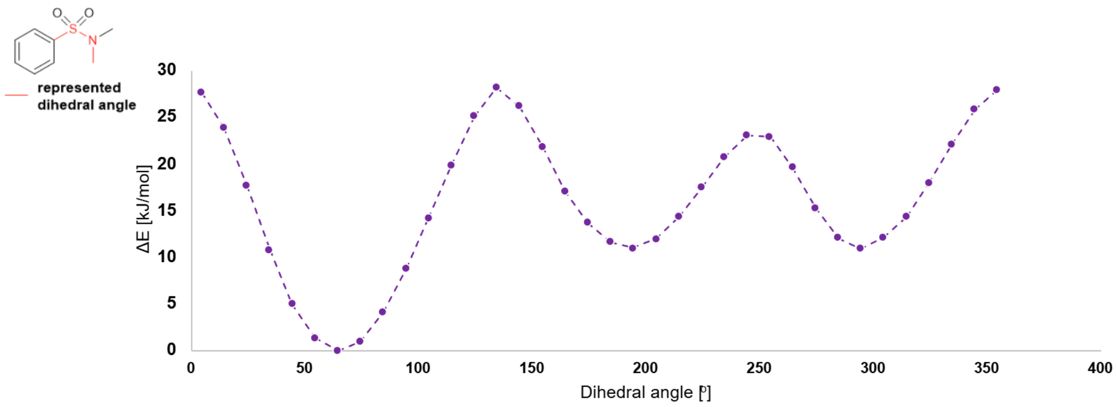

Figure S12: 360º scan of dihedral angle in sulfonamide **S6c**. Minima used as starting geometries for stable minima optimization, maxima used as starting geometries for TS optimization.

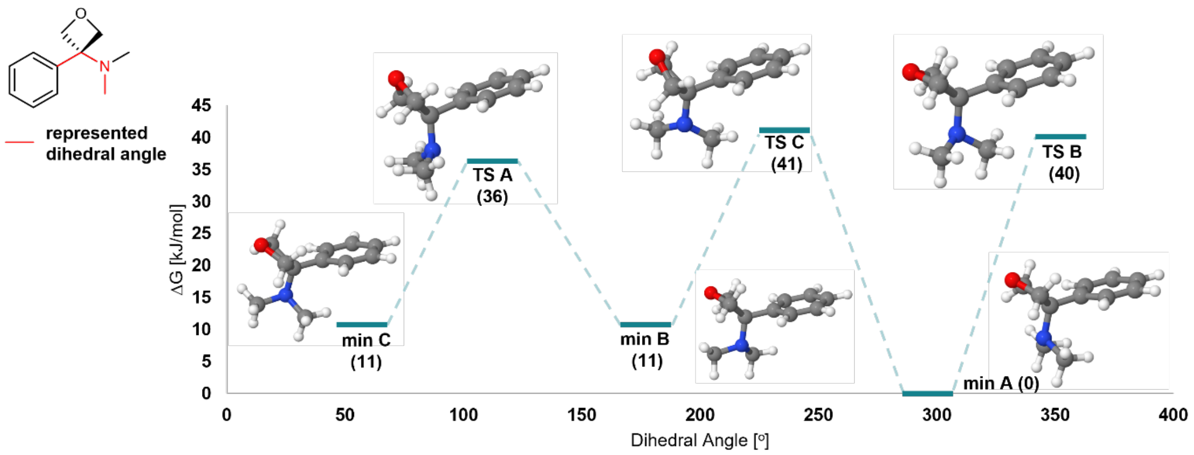

Figure S13: Stable minima and TS of rotation around C-N bond in amino-oxetane **S6a**. Calculations performed at B3LYP/6311G+dp-GD3BJ, SMD=water level of theory. ΔG quoted in kJ/mol compared to the lowest energy minima.

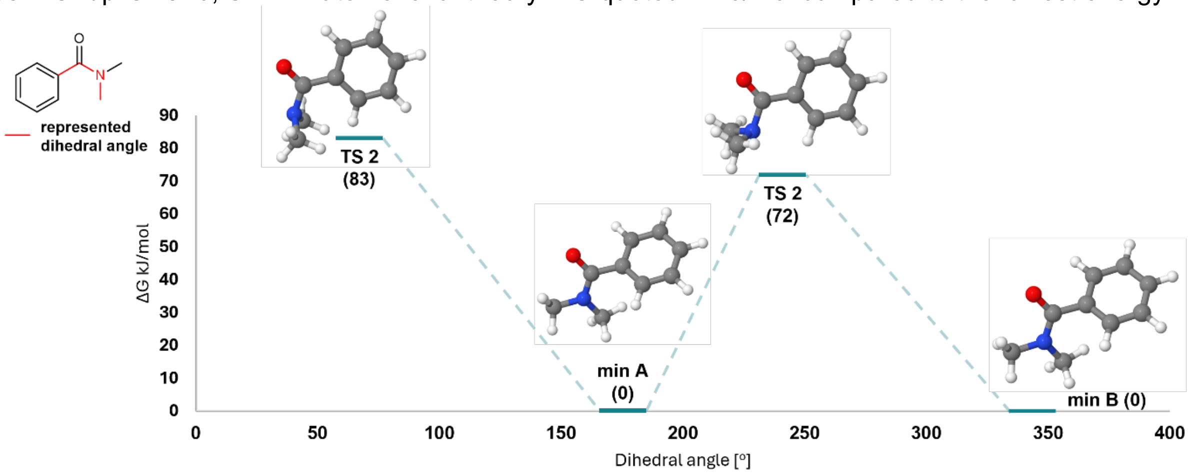

Figure S14: Stable minima and TS of rotation around C-N bond in benzamide **S6b**. Calculations performed at B3LYP/6311G+dp-GD3BJ, SMD=water level of theory. ΔG quoted in kJ/mol compared to the lowest energy minima.

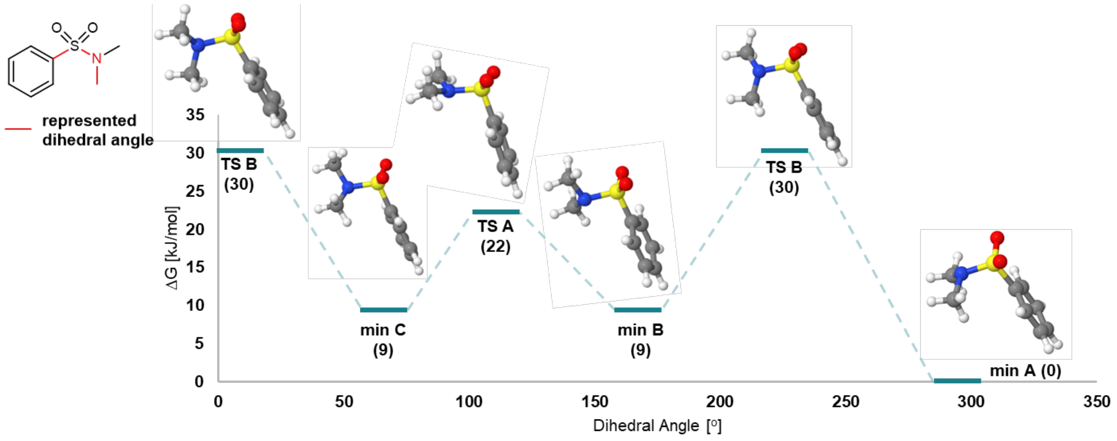

Figure S15: Stable minima and TS of rotation around C-N bond in sulfonamide **S6c**. Calculations performed at B3LYP/6311G+dp-GD3BJ, SMD=water level of theory. ΔG quoted in kJ/mol compared to the lowest energy minima.

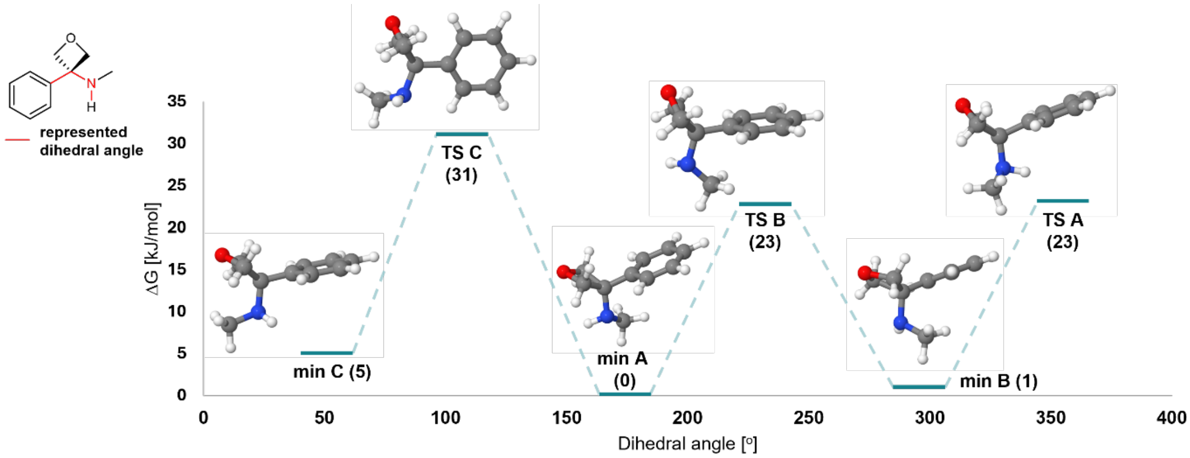

Figure S16: Stable minima and TS of rotation around C-N bond in amino-oxetane **19a**. Calculations performed at B3LYP/6311G+dp-GD3BJ, SMD=water level of theory. ΔG quoted in kJ/mol compared to the lowest energy minima.

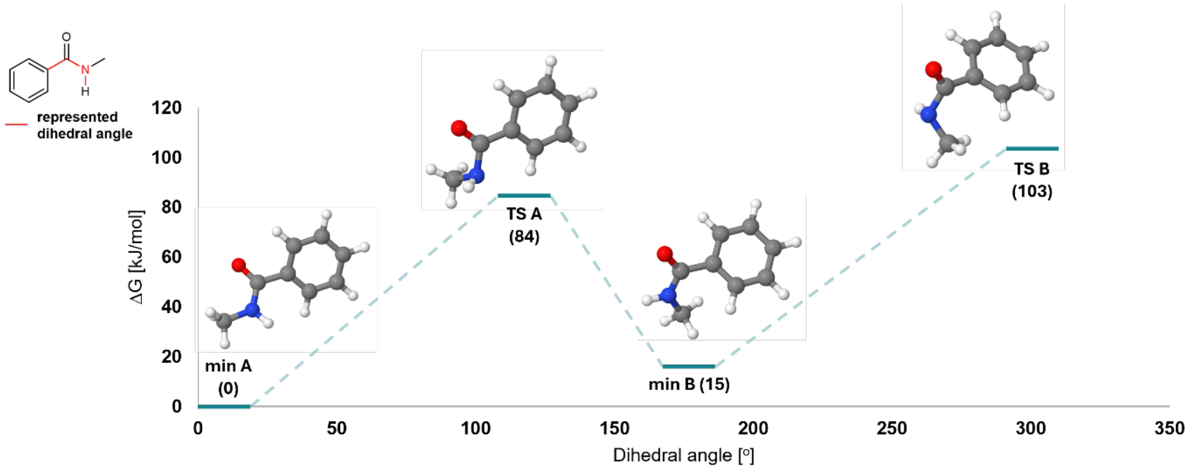

Figure S17: Stable minima and TS of rotation around C-N bond in benzamide **19b**. Calculations performed at B3LYP/6311G+dp-GD3BJ, SMD=water level of theory. ΔG quoted in kJ/mol compared to the lowest energy minima.

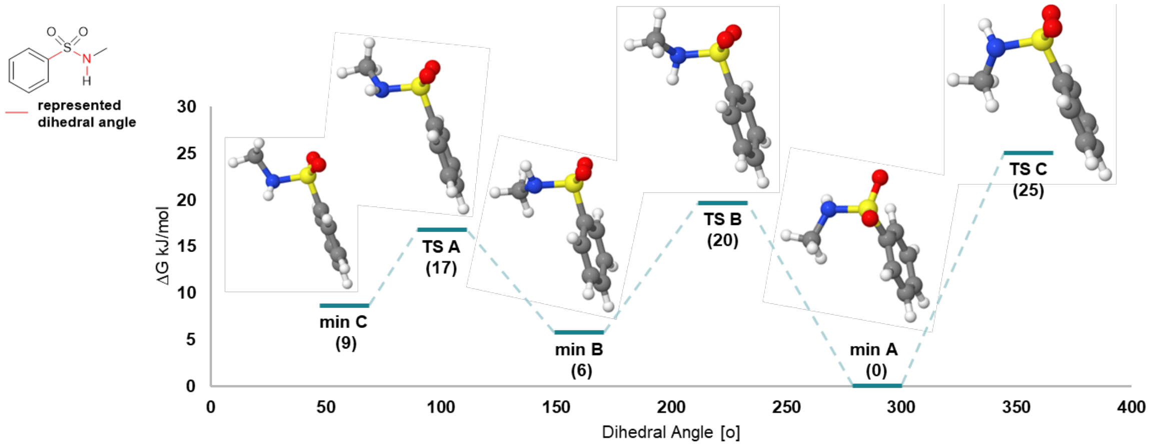

Figure S18: Stable minima and TS of rotation around C-N bond in sulfonamide **19c**. Calculations performed at B3LYP/6311G+dp-GD3BJ, SMD=water level of theory. ΔG quoted in kJ/mol compared to the lowest energy minima.

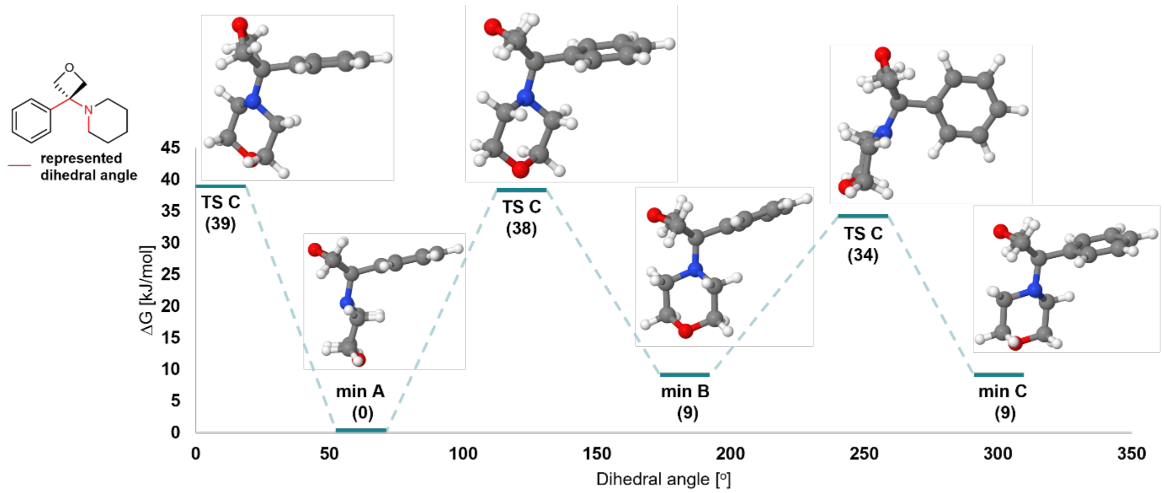

Figure S19: Stable minima and TS of rotation around C-N bond in amino-oxetane **S7a**. Calculations performed at B3LYP/6311G+dp-GD3BJ, SMD=water level of theory. ΔG quoted in kJ/mol compared to the lowest energy minima.

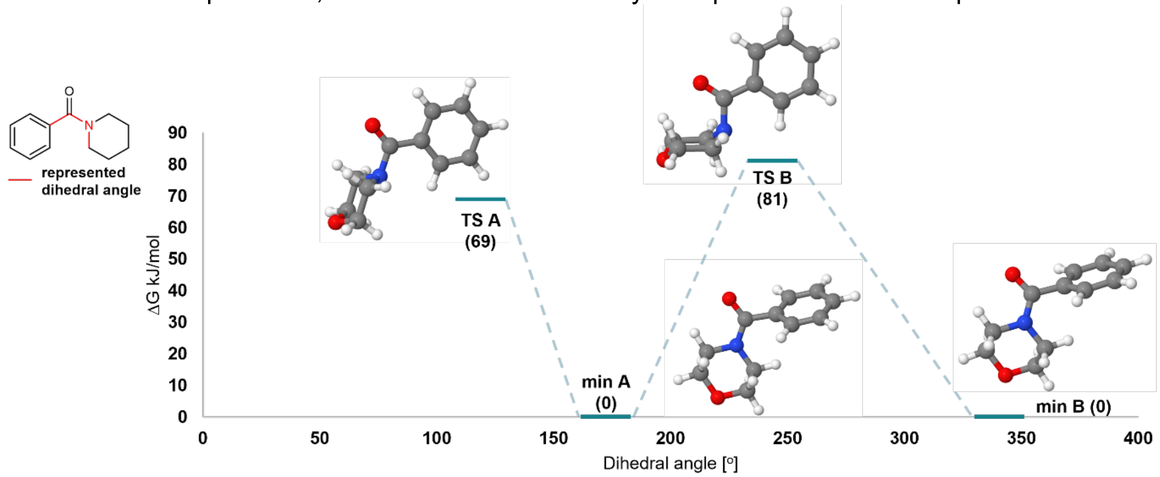

Figure S20: Stable minima and TS of rotation around C-N bond in benzamide **S7b**. Calculations performed at B3LYP/6311G+dp-GD3BJ, SMD=water level of theory. ΔG quoted in kJ/mol compared to the lowest energy minima.

Conformational analysis:

Conformers of **21a-b, 22a** and **22c** were generated in water (*alpb*) at GFN2-xTB level using CREST 2.12 with the following parameters (--ewin = 4 kcal/mol, --ethr = 0.10 kcal/mol and --rthr = 0.250 Å).

The conformer ensembles (provided in SI) were then sorted using CENSO 2.1.2 with the following parameters:

- Pre-screening: PBE-D4/def2-SV(P), alpb =water, with a threshold of 4 kcal/mol
- Screening: r2scan-3c/def2-TZVP, smd = water, with a threshold of 3.5 kcal/mol
- Optimization: r2scan-3c/def2-TZVP, smd = water, with a threshold of 1.5 kcal/mol
- No refinement (False)

The three lowest conformers from the CENSO refinement were then further optimized (incl. *freq* calculations) at B3LYP/6-311G+(d,p)-GD3BJ, SMD=water level of theory (see general considerations).

Conformer ensembles and log files from CREST and CENSO are provided in supporting information.

Distortion analysis:

The energy of distortion in the dihedral angle between a ‘bound’ drug molecule and a ‘free’ structure of the same compound was evaluated for an amide drug (ethamivan, **21b**) and a sulfonamide drug (amprenavir, **22c**) and their amino-oxetane analogues **21a** and **22a**. Bound structures refer to a version of the molecule bound to its respective drug target taken from the relevant database (ethamivan: PDB - 2xdl,[17] amprenavir: PDB - 3nu3[18]). The investigated dihedral angle was frozen, and the rest of the structure optimized freely (SMD=water). Free structures refer to the lowest energy conformers of ethamivan **21b** and amprenavir **22c** in aqueous solvation.

The initial lowest energy conformers were selected based on the minima of typical amide or sulfonamide structures determined in the rotational studies. The starting conformers were optimized at the B3LYP/6311G+dp-GD3BJ, SMD=water level of theory and then used as a starting point for CREST/CENSO analysis (see details in conformational analysis). The lowest energy structures (as obtained by CREST/CENSO) were used for comparison with the bound structures.

The initial molecular geometries of amino-oxetane analogues **21a** and **22a** were obtained by replacing the amide group in ethamivan **21b** and the sulfonamide group in amprenavir **22c** with an amino-oxetane group, and subsequently optimizing at B3LYP/6311G+dp-GD3BJ, SMD=water level of theory.

The “bound” amino-oxetane structures were obtained via *constrained optimizations* where the dihedral angle was frozen at the same value present in the bound amide and sulfonamide structures.

The “free” amino-oxetane structures were obtained via relaxed optimization (no constrain on the dihedral angles). The lowest free energy conformers were identified using CREST/CENSO (see details in conformational analysis), as per analogous procedure performed for their amide and sulfonamide counterparts.

The distortion energy of the dihedral angle was evaluated by calculating the energy differences between the bound and the free structures of each compound (Tables S3–S6). The four compounds evaluated are ethamivan **21b**, amprenavir **22c** as well as their amino-oxetane analogues **21a** and **22a**, respectively.

Different level of theories were also investigated. Both the bound and the free structures obtained at B3LYP/6311G+dp-GD3BJ, SMD=water level of theory were also re-optimized at M06-2X/6311G+dp,SMD=water and WB97XD/6311G+dp, SMD=water levels of theory.

Table S5: Energy differences between ethamivan **21b** 'bound' and 'free' at B3LYP/6311G+dp-GD3BJ, M06-2X/6311G+dp and WB97XD/6311G+dp level with aqueous solvation (SMD).

kcal mol^{-1}	ΔG_{298K}	ΔH	$T\Delta S_{298K}$
B3LYP/6311G+dp-GD3BJ, SMD=water			
Ethamivan (free)	0	0	0
Ethamivan (bound)	2.1	2.6	0.5
M06-2X/6311G+dp,SMD=water			
Ethamivan (free)	0	0	0
Ethamivan (bound)	2.6	2.2	–0.5
WB97XD/6311G+dp,SMD=water			
Ethamivan (free)	0	0	0
Ethamivan (bound)	1.7	2.4	0.7

Table S6: Energy differences between ethamivan analogue amino-oxetane **21a** 'bound' and 'free' at B3LYP/6311G+dp-GD3BJ, M06-2X/6311G+dp and WB97XD/6311G+dp level with aqueous solvation (SMD).

kcal mol^{-1}	ΔG_{298K}	ΔH	$T\Delta S_{298K}$
B3LYP/6311G+dp-GD3BJ,SMD=water			
Amino-oxetane **21a** (free)	0	0	0
Amino-oxetane **21a** (bound)	5.1	5.5	0.5
M06-2X/6311G+dp,SMD=water			
Amino-oxetane **21a** (free)	0	0	0
Amino-oxetane **21a** (bound)	5.1	5.5	0.5
WB97XD/6311G+dp,SMD=water			
Amino-oxetane **21a** (free)	0	0	0
Amino-oxetane **21a** (bound)	5.3	5.7	0.5

Table S7: Energy differences between amprenavir **22c** 'bound' and 'free' at B3LYP/6311G+dp-GD3BJ, M06-2X/6311G+dp and WB97XD/6311G+dp level with aqueous solvation (SMD).

kcal mol^{-1}	ΔG_{298K}	ΔH	$T\Delta S_{298K}$
B3LYP/6311G+dp-GD3BJ,SMD=water			
Amprenavir (free)	0	0	0
Amprenavir (bound)	3.8	4.5	0.7
M06-2X/6311G+dp,SMD=water			
Amprenavir (free)	0	0	0
Amprenavir (bound)	2.4	3.6	1.4
WB97XD/6311G+dp,SMD=water			
Amprenavir (free)	0	0	0
Amprenavir (bound)	3.6	5.0	1.2

Table S8: Energies between amprenavir amino-oxetane analogue **22a** 'bound' and 'free' at B3LYP/6311G+dp-GD3BJ, M06-2X/6311G+dp and WB97XD/6311G+dp level with aqueous solvation (SMD).

kcal mol^{-1}	ΔG_{298K}	ΔH	$T\Delta S_{298K}$
B3LYP/6311G+dp-GD3BJ,SMD=water			
Amino-oxetane **22a** (free)	0	0	0
Amino-oxetane **22a** (bound)	3.3	5.0	1.7
M06-2X/6311G+dp,SMD=water			
Amino-oxetane **22a** (free)	0	0	0
Amino-oxetane **22a** (bound)	1.4	4.3	2.9
WB97XD/6311G+dp,SMD=water			
Amino-oxetane **22a** (free)	0	0	0
Amino-oxetane **22a** (bound)	3.1	5.3	2.2

Purity by Absolute Quantitative ^{1}H NMR Spectroscopy

Representative examples of the determination of purity by absolute quantitative ^{1}H NMR spectroscopy are shown below. The stated amount of sample and stated amount of 1,3,5-trimethoxybenzene (≥99%) was dissolved in $CDCl_3$ (600 μL) in a 5 mm standard NMR tube. The tube was sealed and the ^{1}H NMR spectrum was collected on a 400 MHz AVANCE III HD NanoBay console equipped with a Bruker BioSpin BBO probe using the parameters below:

Pulse program: zg30
Sample temperature: 296 K
Data points: 64K
Zero-Filling (SI): 64K
Pulse width (PW): 30°
Relaxation delay (D1): 30 s
Scans (NS): 16
Acquisition time: 4.089 s
Spectral Window (SW): 20 ppm
Transmitter Offset (O1P): 6 ppm

The raw data was processed using MestreNova with a full auto phase correction and a full auto Bernstein polynomial baseline correction.

Sample of **5b** (8.0 mg mL⁻¹) in $CDCl_3$ with addition of 1,3,5-trimethoxybenzene (TMB, ≥99%) as an internal standard.

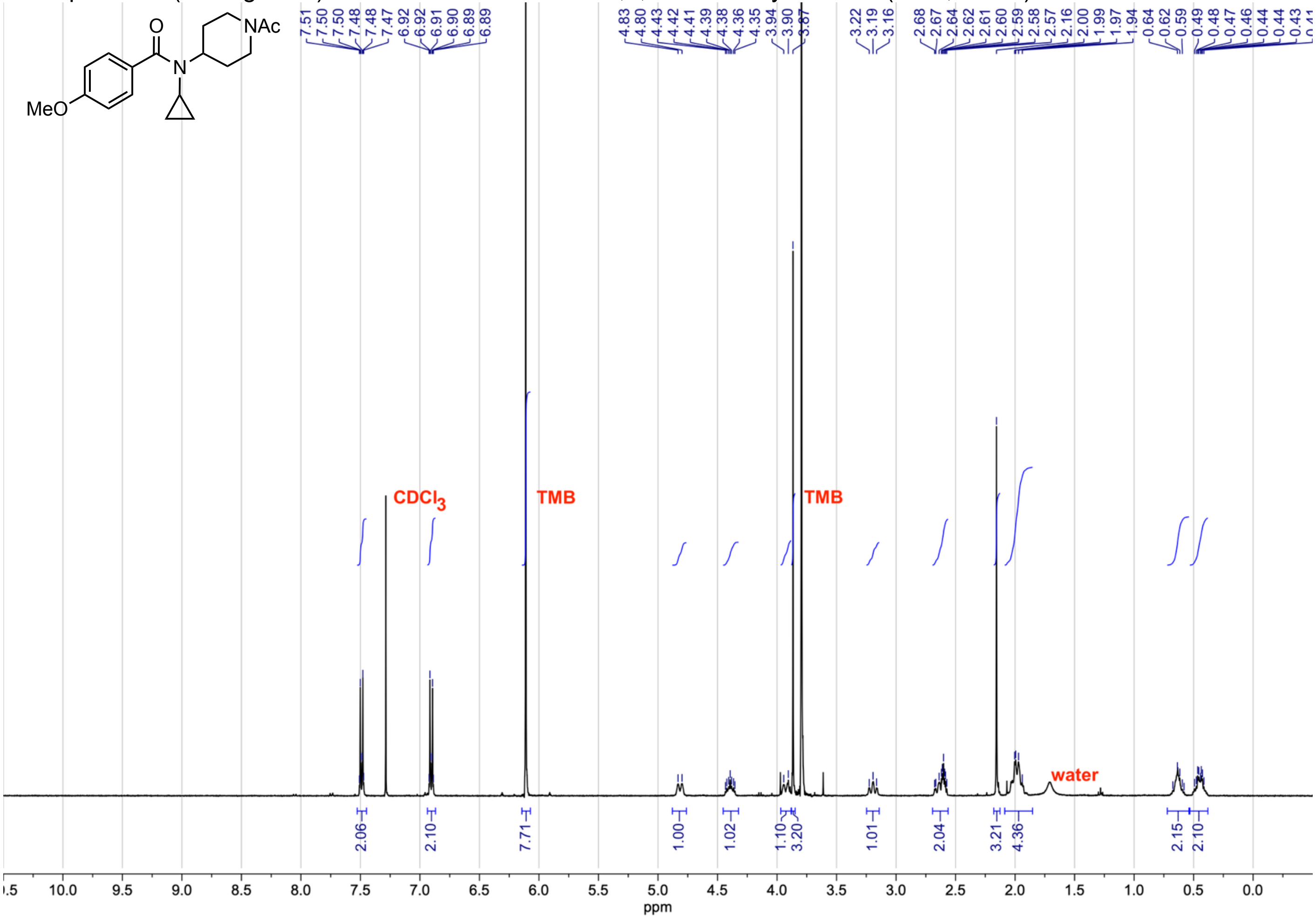

m_s = 4.80 mg, m_{IC} = 6.10 mg, P_{IC} = 99%

Int_t^a = 1.06, n_t = 1

Int_{IC} = 7.71, n_{IC} = 3

MW_t = 316.40 g mol⁻¹, MW_{IC} = 168.19 g mol⁻¹

$$P[\%] = \frac{n_{IC}\times Int_t \times MW_t \times m_{IC} \times P_{IC}}{n_t \times Int_{IC} \times MW_{IC} \times m_s} = 97.6\%$$

[a]The integral of the target analyte was calculated as the average of signals at 7.53–7.45, 6.94–6.87, 4.81, 4.39, 3.92, 3.87, 3.19, 2.69–2.56, 2.16, 2.09–1.85, 0.63, and 0.46 ppm.

Sample of **8a** (11.3 mg mL^{-1}) in $CDCl_3$ with addition of 1,3,5-trimethoxybenzene (TMB, ≥99%) as an internal standard.

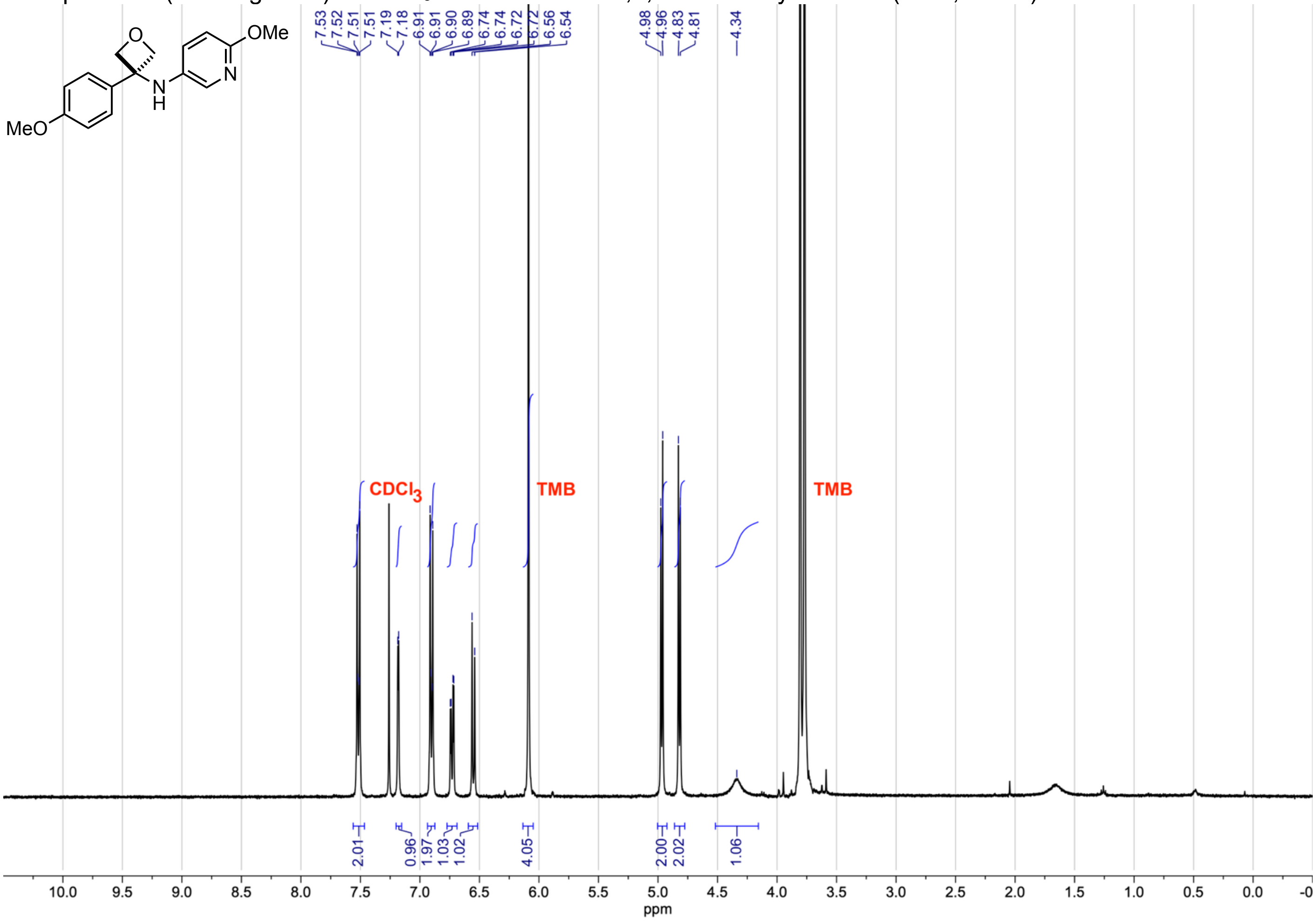

m_s = 6.80 mg, m_{IC} = 5.40 mg, P_{IC} = 99%

$Int_t^{a,b}$ = 1.01, n_t = 1

Int_{IC} = 4.05, n_{IC} = 3

MW_t = 286.33 g mol^{-1}, MW_{IC} = 168.19 g mol^{-1}

$$P[\%] = \frac{n_{IC} \times Int_t \times MW_t \times m_{IC} \times P_{IC}}{n_t \times Int_{IC} \times MW_{IC} \times m_s} = 100.1\%^{c}$$

[a]The integral of the target analyte was calculated as the average of signals at 7.56–7.47, 7.18, 6.94–6.87, 6.73, 6.55, 4.97, 4.82, 4.34 ppm.

[b]The signals of the methoxy protons were not included/integrated due to overlap with the methoxy signals from the internal standard (1,3,5-trimethoxybenzene).

[c]The +0.1% difference between the determined purity (100.1%) and the theoretical maximum of 100% is well within the accuracy of typical laboratory settings (NMR and balance validation).

Sample of **9a** (19.5 mg mL^{-1}) in $CDCl_3$ with addition of 1,3,5-trimethoxybenzene (TMB, ≥99%) as an internal standard.

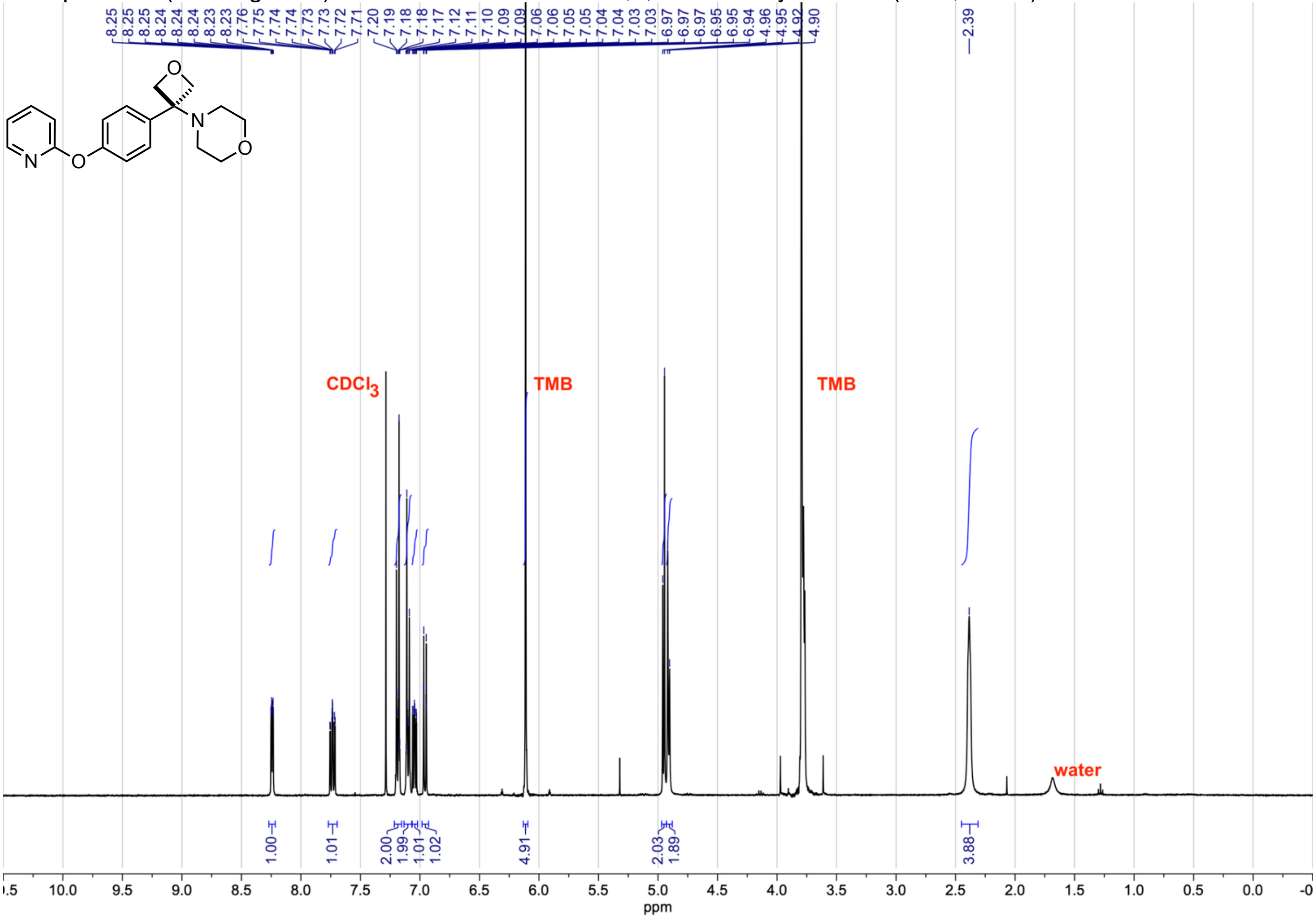

m_s = 11.7 mg, m_{IC} = 10.3 mg, P_{IC} = 99%

$Int_t^{a,b}$ = 0.99, n_t = 1

Int_{IC} = 4.91, n_{IC} = 3

MW_t = 312.36 g mol^{-1}, MW_{IC} = 168.19 g mol^{-1}

$$P[\%] = \frac{n_{IC} \times Int_t \times MW_t \times m_{IC} \times P_{IC}}{n_t \times Int_{IC} \times MW_{IC} \times m_s} = 97.9\%$$

[a]The integral of the target analyte was calculated as the average of signals at 8.24, 7.73, 7.21–7.15, 7.13–7.07, 7.05, 6.96, 4.95, 4.91, 2.39 ppm.

[b]The signals of the morpholine protons adjacent to the oxygen were not included/integrated due to overlap with the methoxy signals from the internal standard (1,3,5-trimethoxybenzene).

Sample of **9b** (7.3 mg mL^{-1}) in $CDCl_3$ with addition of 1,3,5-trimethoxybenzene (TMB, ≥99%) as an internal standard.

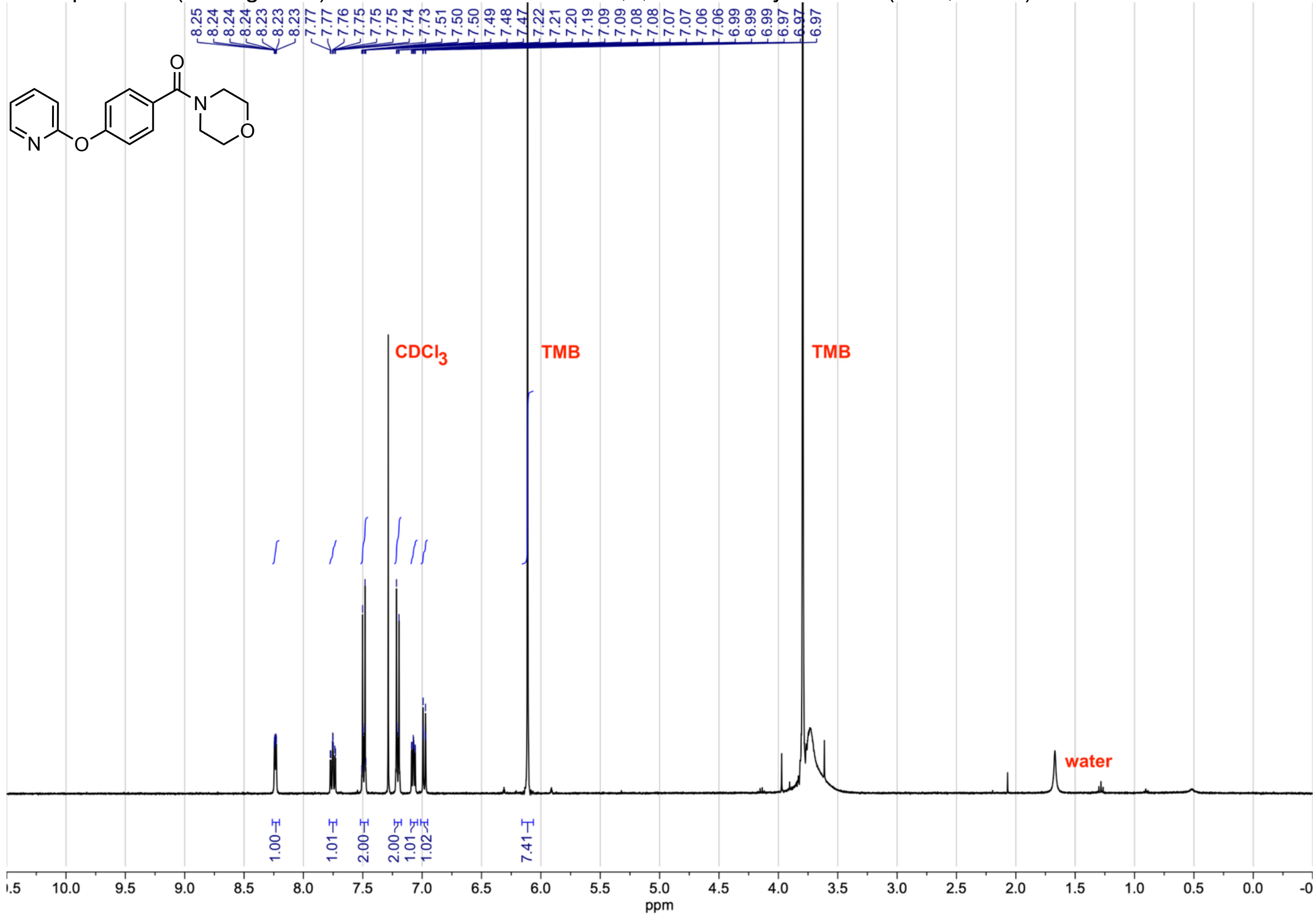

m_s = 4.40 mg, m_{IC} = 6.30 mg, P_{IC} = 99%

$Int_t^{a,b}$ = 1.01, n_t = 1

Int_{IC} = 7.41, n_{IC} = 3

MW_t = 284.31 g mol^{-1}, MW_{IC} = 168.19 g mol^{-1}

$$P[\%] = \frac{n_{IC} \times Int_t \times MW_t \times m_{IC} \times P_{IC}}{n_t \times Int_{IC} \times MW_{IC} \times m_s} = 98.0\%$$

[a]The integral of the target analyte was calculated as the average of signals at 8.26–8.20, 7.75, 7.52–7.46, 7.23–7.18, 7.07, 6.98 ppm.

[b]The signals of the morpholine protons were not included/integrated due to overlap with the methoxy signals from the internal standard (1,3,5-trimethoxybenzene).

Sample of **10b** (7.5 mg mL^{-1}) in $CDCl_3$ with addition of 1,3,5-trimethoxybenzene (TMB, ≥99%) as an internal standard.

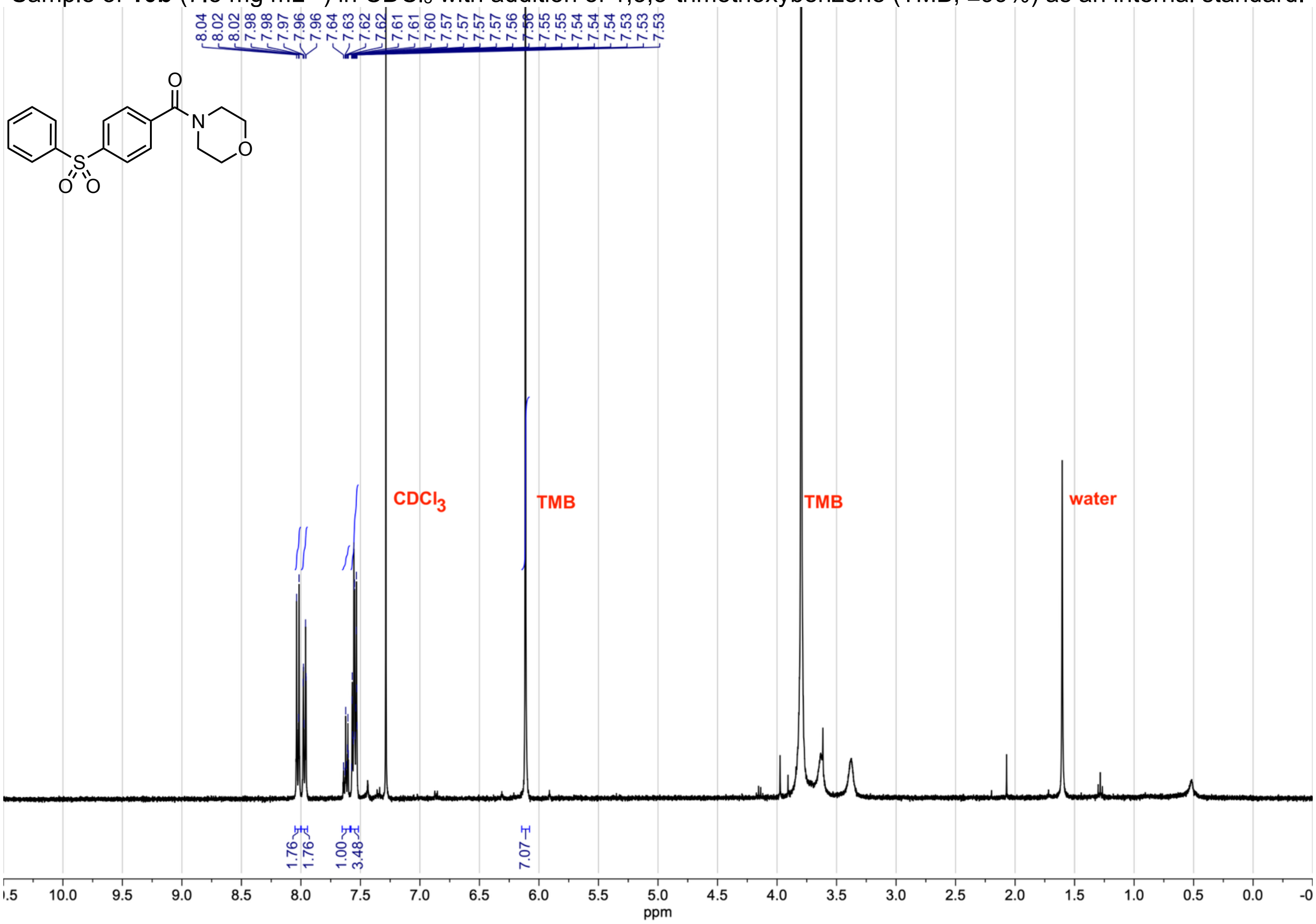

m_s = 4.50 mg, m_{IC} = 6.00 mg, P_{IC} = 99%

$Int_t^{a,b}$ = 0.89, n_t = 1

Int_{IC} = 7.07, n_{IC} = 3

MW_t = 331.39 g mol^{-1}, MW_{IC} = 168.19 g mol^{-1}

$$P[\%] = \frac{n_{IC} \times Int_t \times MW_t \times m_{IC} \times P_{IC}}{n_t \times Int_{IC} \times MW_{IC} \times m_s} = 98.2\%$$

[a]The integral of the target analyte was calculated as the average of signals at 8.03, 8.00–7.94, 7.65–7.59, 7.58–7.52 ppm.

[b]The signals of the morpholine protons were not included/integrated due to overlap with the methoxy signals from the internal standard (1,3,5-trimethoxybenzene).

Sample of **11a** (17.7 mg mL^{-1}) in $CDCl_3$ with addition of 1,3,5-trimethoxybenzene (TMB, ≥99%) as an internal standard.

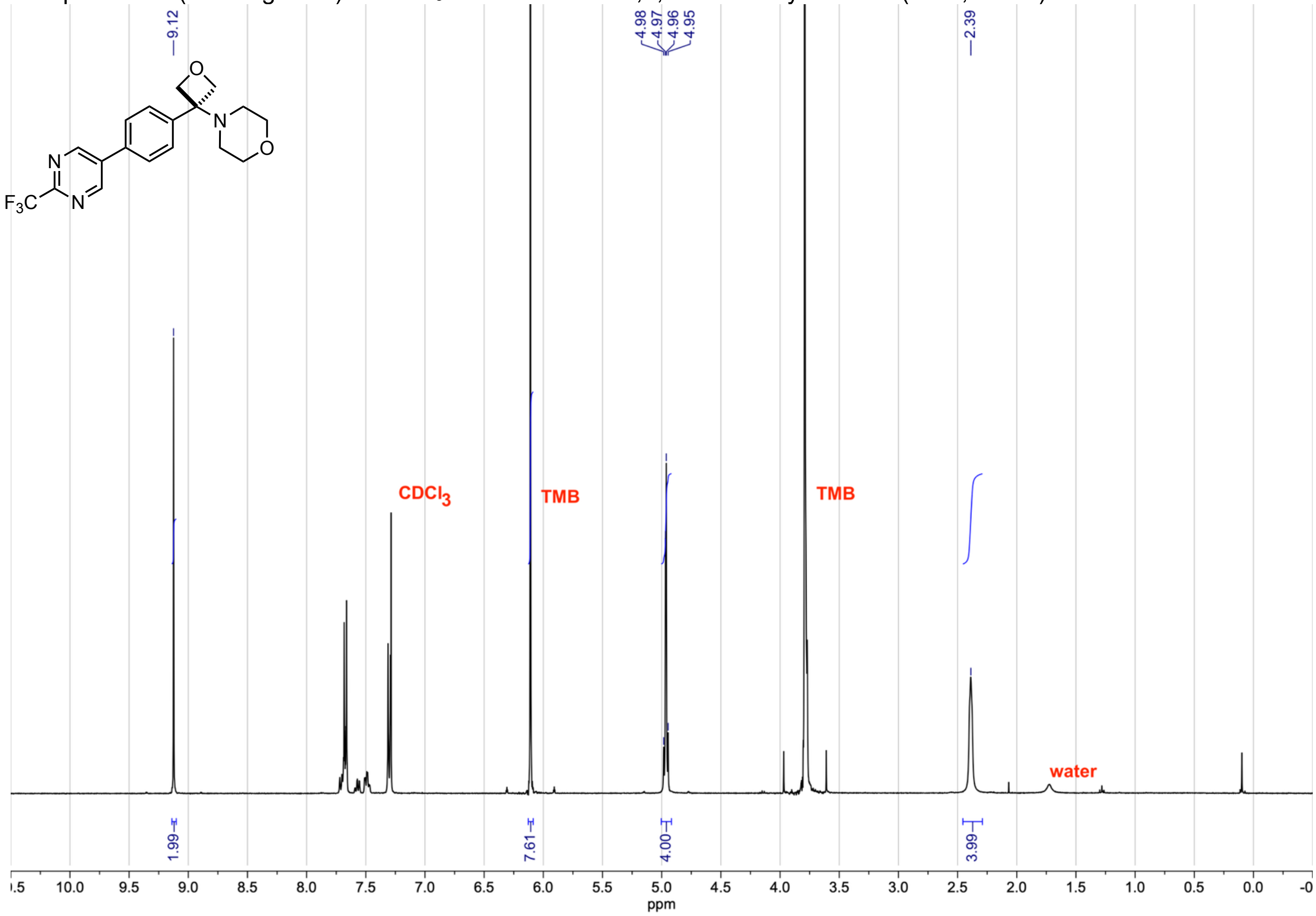

m_s = 10.6 mg, m_{IC} = 12.1 mg, P_{IC} = 99%

$Int_t^{a,b}$ = 0.99, n_t = 1

Int_{IC} = 7.61, n_{IC} = 3

MW_t = 365.35 g mol^{-1}, MW_{IC} = 168.19 g mol^{-1}

$$P[\%] = \frac{n_{IC} \times Int_t \times MW_t \times m_{IC} \times P_{IC}}{n_t \times Int_{IC} \times MW_{IC} \times m_s} = 95.8\%$$

[a]The integral of the target analyte was calculated as the average of signals at 9.12, 5.00–4.92, 2.39 ppm.

[b]The signals of the aromatic protons were not included/integrated due to overlap with an unknown aromatic impurity and the signal from $CDCl_3$.

Sample of **11b** (6.67 mg mL^{-1}) in $CDCl_3$ with addition of 1,3,5-trimethoxybenzene (TMB, ≥99%) as an internal standard.

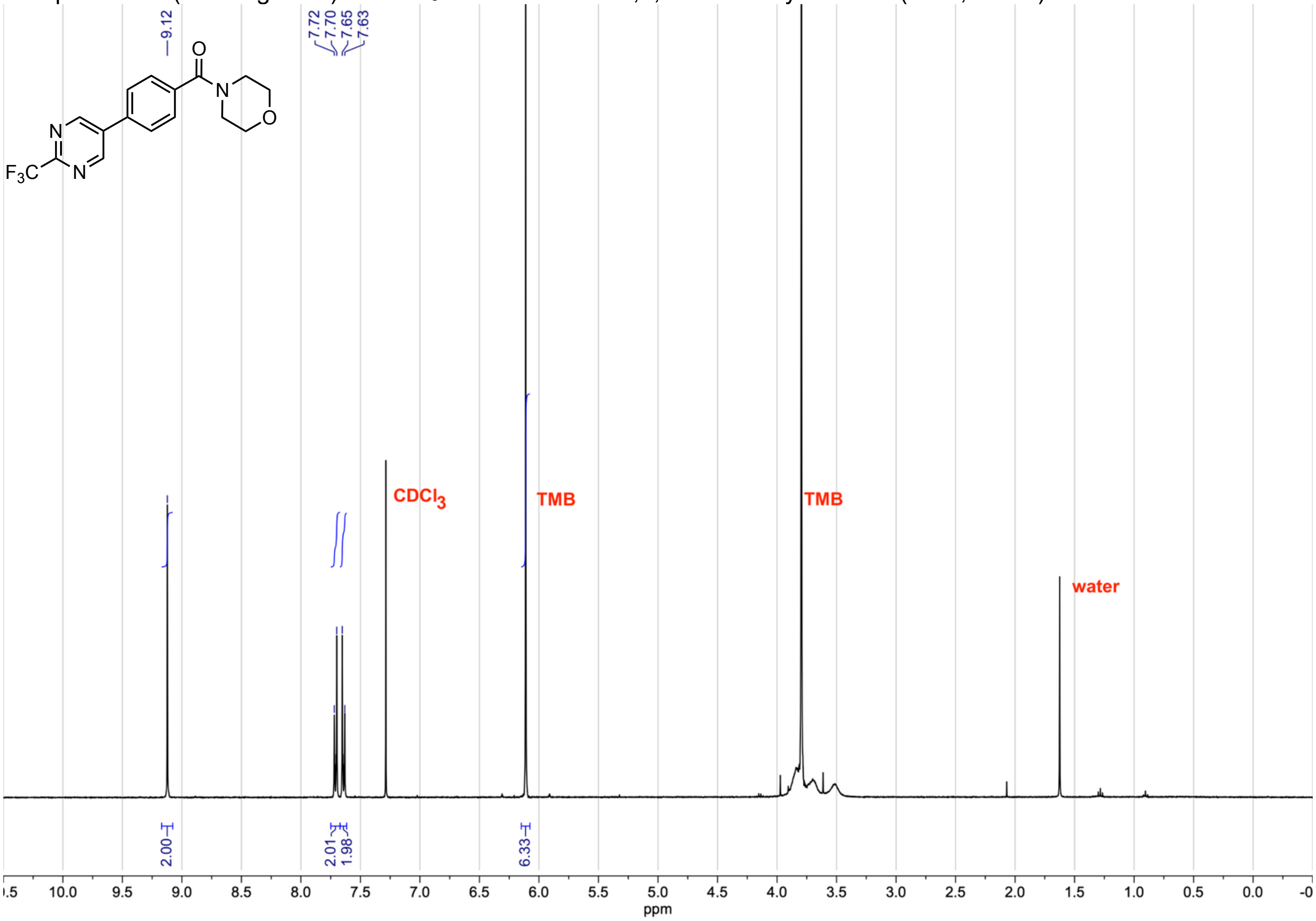

$$m_s = 4.00 \text{ mg}, m_{IC} = 4.30 \text{ mg}, P_{IC} = 99\%$$
$$Int_t^{a,b} = 0.99, n_t = 1$$
$$Int_{IC} = 6.33, n_{IC} = 3$$
$$MW_t = 337.30 \text{ g mol}^{-1}, MW_{IC} = 168.19 \text{ g mol}^{-1}$$
$$P[\%] = \frac{n_{IC} \times Int_t \times MW_t \times m_{IC} \times P_{IC}}{n_t \times Int_{IC} \times MW_{IC} \times m_s} = 100.1\%^{c}$$

[a]The integral of the target analyte was calculated as the average of signals at 9.12, 7.71, 7.64 ppm.
[b] The signals of the morpholine protons were not included/integrated due to overlap with the methoxy signals from the internal standard (1,3,5-trimethoxybenzene).
[c]The +0.1% difference between the determined purity (100.1%) and the theoretical maximum of 100% is well within the accuracy of typical laboratory settings (NMR and balance validation).

References

1. Cui, X.; Zhang, Y.; Shi, F.; Deng, Y. *Chem. Eur. J.* **2011**, *17* (9), 2587–2591.
2. Chemicalize was used for prediction of Log*P*, Log*D*, TPSA, and pK_a, accessed December 2025, https://chemicalize.com/, developed by ChemAxon.
3. Viswanadhan, V. N.; Ghose, A. K.; Revankar, G. R.; Robins, R. K. *J. Chem. Inf. Comput. Sci.* **1989**, *29* (3), 163–172.
4. Csizmadia, F.; Tsantili-Kakoulidou, A.; Panderi, I.; Darvas, F. *J. Pharm. Sci.* **1997**, *86* (7), 865–871.
5. Ertl, P.; Rohde, B.; Selzer, P. *J. Med. Chem.* **2000**, *43* (20), 3714–3717.
6. Szegezdi, J.; Csizmadia, F. Prediction of Dissociation Constant Using Microconstants. Presented at the *American Chemical Society National Meeting*; Anaheim, CA, 2004.
7. Wager, T. T.; Hou, X.; Verhoest, P. R.; Villalobos, A. *ACS Chem. Neurosci.* **2010**, *1* (6), 435–449.
8. Wilson, D. M.; Wang, X.; Walsh, E.; Rourick, R. A. *Comb. Chem. High Throughput Screen* **2001**, *4* (6), 511–519.
9. Di, L.; Whitney-Pickett, C.; Umland, J. P.; Zhang, H.; Zhang, X.; Gebhard, D. F.; Lai, Y.; Federico, J. J.; Davidson, R. E.; Smith, R.; Reyner, E. L.; Lee, C.; Feng, B.; Rotter, C.; Varma, M. V.; Kempshall, S.; Fenner, K.; El-kattan, A. F.; Liston, T. E.; Troutman, M. D. *J. Pharm. Sci*. **2011**, *100*, 4974–4985.
10. Dolomanov, O. V.; Bourhis, L. J.; Gildea, R. J.; Howard, J. A. K.; Puschmann, H. *J. Appl. Crystallogr*. **2009**, *42* (2), 339–341.
11. SHELXTL v5.1, Bruker AXS, Madison, WI, 1998.
12. SHELX-2013, Sheldrick, G. M. *Acta Crystallogr. C Struct. Chem*. **2015**, *71* (1), 3–8.
13. A.L. Spek (2003, 2009) PLATON, A Multipurpose Crystallographic Tool, Utrecht University, Utrecht, The Netherlands. See also Spek, A. L. *Acta Crystallogr. C Struct. Chem.* **2015**, *71* (1), 9–18.
14. M. J. Frisch, G. W. Trucks, H. B. Schlegel, G. E. Scuseria, M. A. Robb, J. R. Cheeseman, G. Scalmani, V. Barone, G. A. Petersson, H. Nakatsuji, X. Li, M. Caricato, A. V. Marenich, J. Bloino, B. G. Janesko, R. Gomperts, B. Mennucci, H. P. Hratchian, J. V. Ortiz, A. F. Izmaylov, J. L. Sonnenberg, D. Williams-Young, F. Ding, F. Lipparini, F. Egidi, J. Goings, B. Peng, A. Petrone, T. Henderson, D. Ranasinghe, V. G. Zakrzewski, J. Gao, N. Rega, G. Zheng, W. Liang, M. Hada, M. Ehara, K. Toyota, R. Fukuda, J. Hasegawa, M. Ishida, T. Nakajima, Y. Honda, O. Kitao, H. Nakai, T. Vreven, K. Throssell, J. A. Montgomery, Jr., J. E. Peralta, F. Ogliaro, M. J. Bearpark, J. J. Heyd, E. N. Brothers, K. N. Kudin, V. N. Staroverov, T. A. Keith, R. Kobayashi, J. Normand, K. Raghavachari, A. P. Rendell, J. C. Burant, S. S. Iyengar, J. Tomasi, M. Cossi, J. M. Millam, M. Klene, C. Adamo, R. Cammi, J. W. Ochterski, R. L. Martin, K. Morokuma, O. Farkas, J. B. Foresman, and D. J. Fox, Gaussian, Inc., Wallingford CT, 2019.
15. Glendening, E. D.; Badenhoop, J, K.; Reed, A. E.; Carpenter, J. E.; Bohmann, J. A.; Morales, C. M.; Karafiloglou, P.; Landis, C. R.; Weinhold, F. NBO 7.0. Theoretical Chemistry Institute, University of Wisconsin, Madison (2018).
16. Pracht, P.; Grimme, S.; Bannwarth, C.; Bohle, F.; Ehlert, S.; Feldmann, G.; Gorges, J.; Müller, M.; Neudecker, T.; Plett, C.; Spicher, S.; Steinbach, P.; Wesołowski, P. A.; Zeller, F. *J. Chem. Phys.* **2024**, *160*, 114110.
17. Murray, C. W.; Carr, M. G.; Callaghan, O.; Chessari, G.; Congreve, M.; Cowan, S.; Coyle, J. E.; Downham, R.; Figueroa, E.; Frederickson, M.; Graham, B.; McMenamin, R.; O'Brien, M. A.; Patel, S.; Phillips, T. R.; Williams, G.; Woodhead, A. J.; Woolford, A. J.-A. *J. Med. Chem.* **2010**, *53* (16), 5942–5955.
18. Shen, C.; Wang, Y.; Kovalevsky, A. Y.; Harrison, R. W.; Weber, I. T. *The FEBS Journal* **2010**, *277* (18), 3699–3714.

^{1}H and ^{13}C NMR Spectra of Novel Compounds

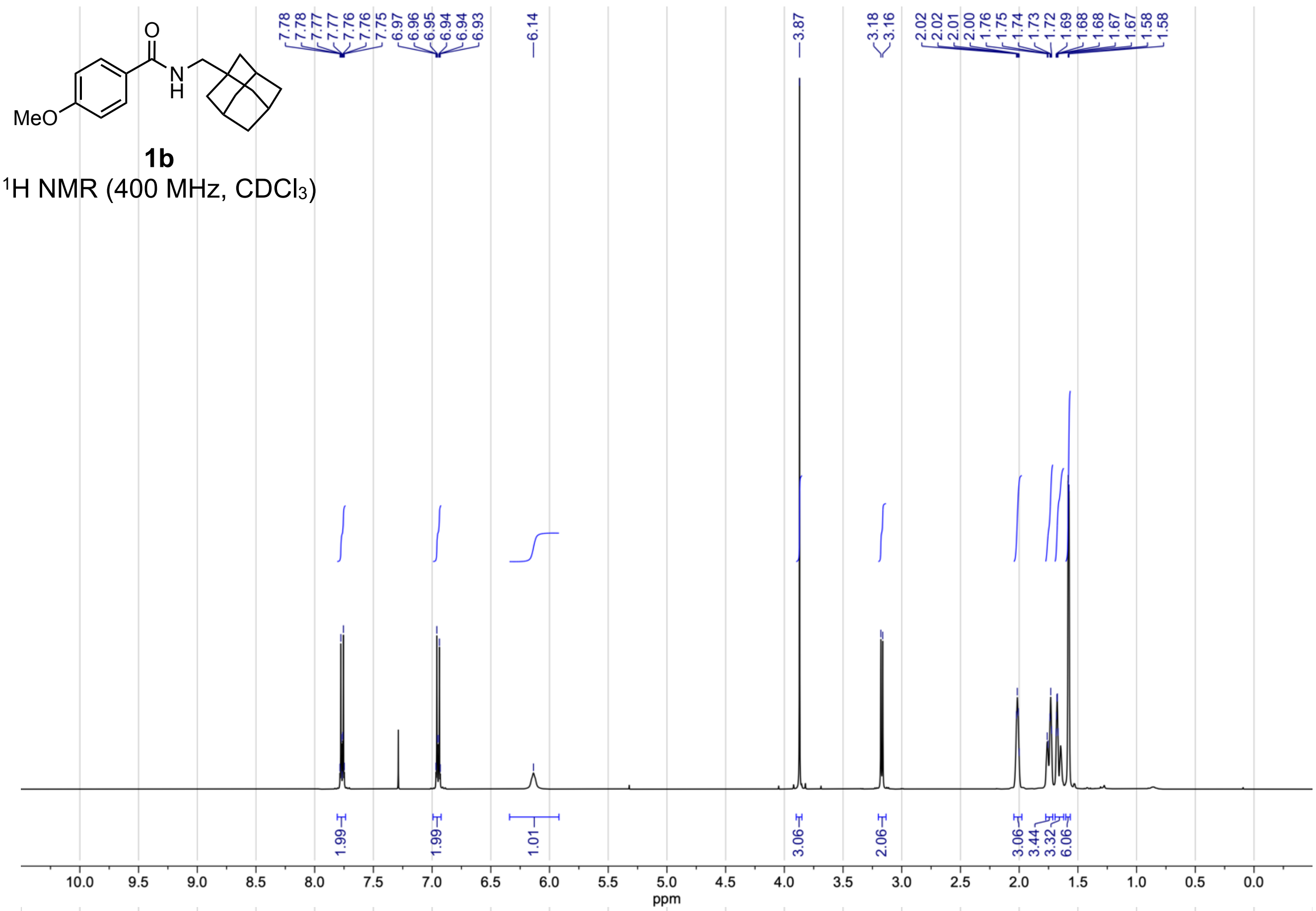

1b

^{1}H NMR (400 MHz, $CDCl_3$)

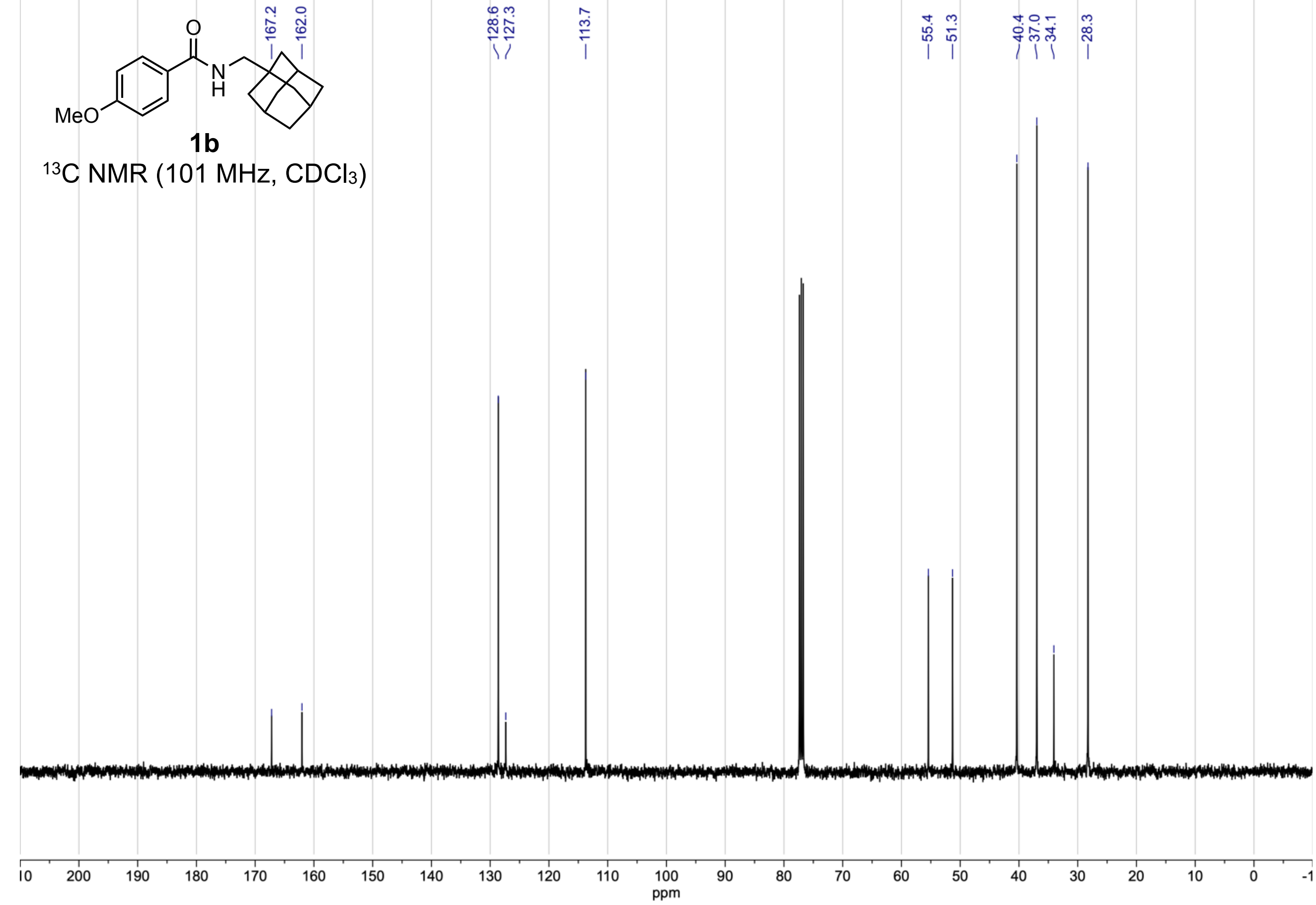

1b

^{13}C NMR (101 MHz, $CDCl_3$)

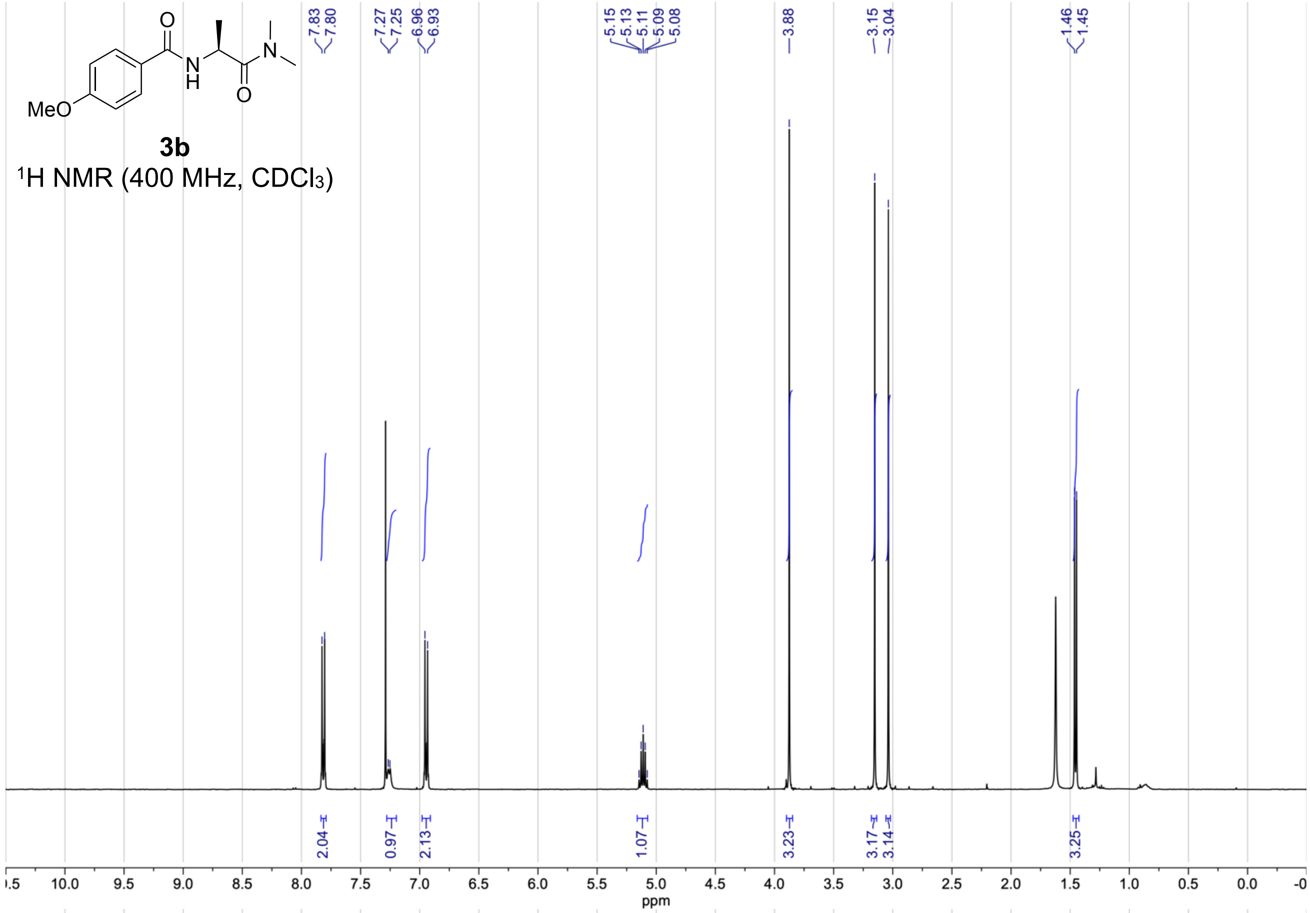

3b
^{1}H NMR (400 MHz, $CDCl_3$)

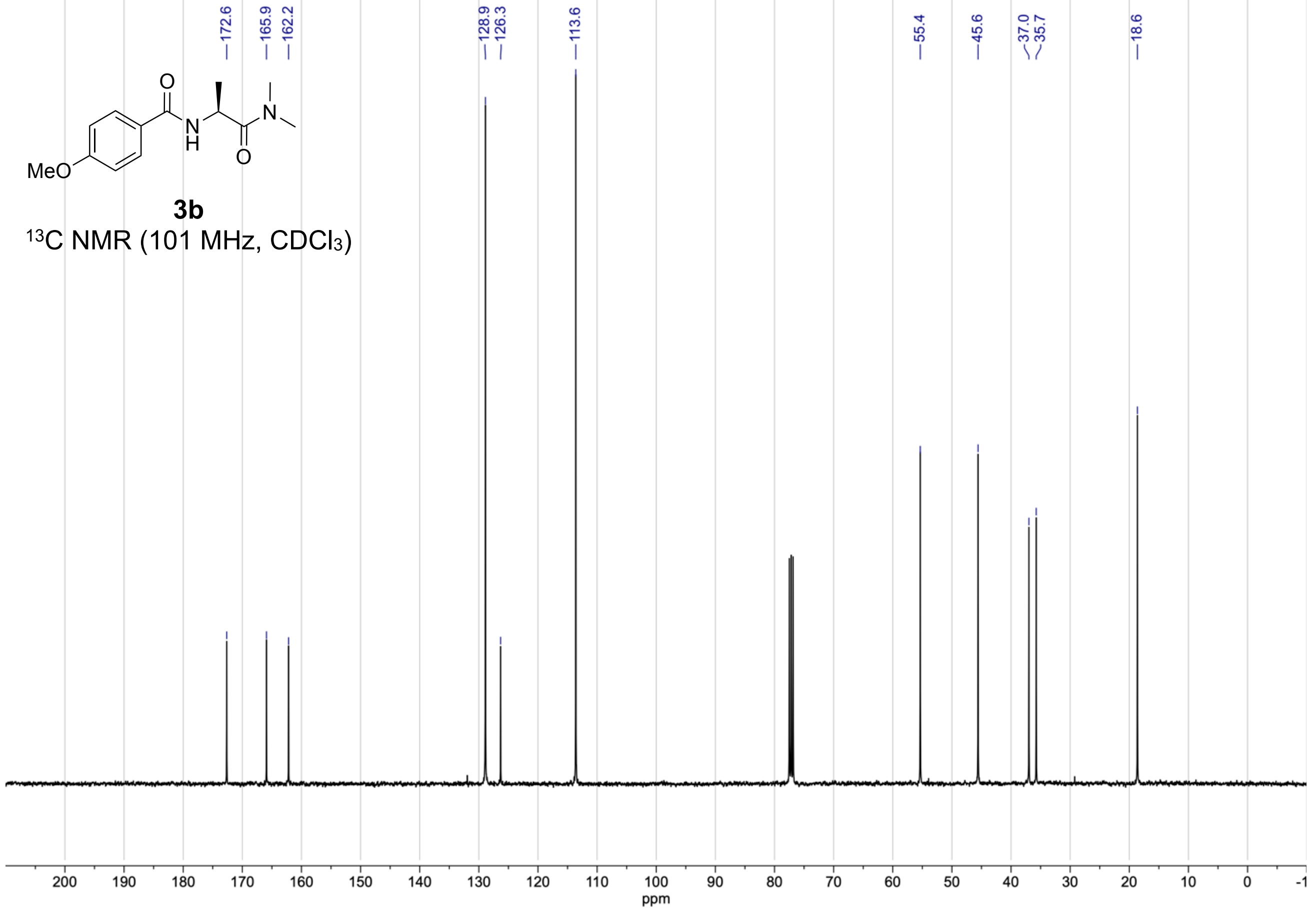

3b
^{13}C NMR (101 MHz, $CDCl_3$)

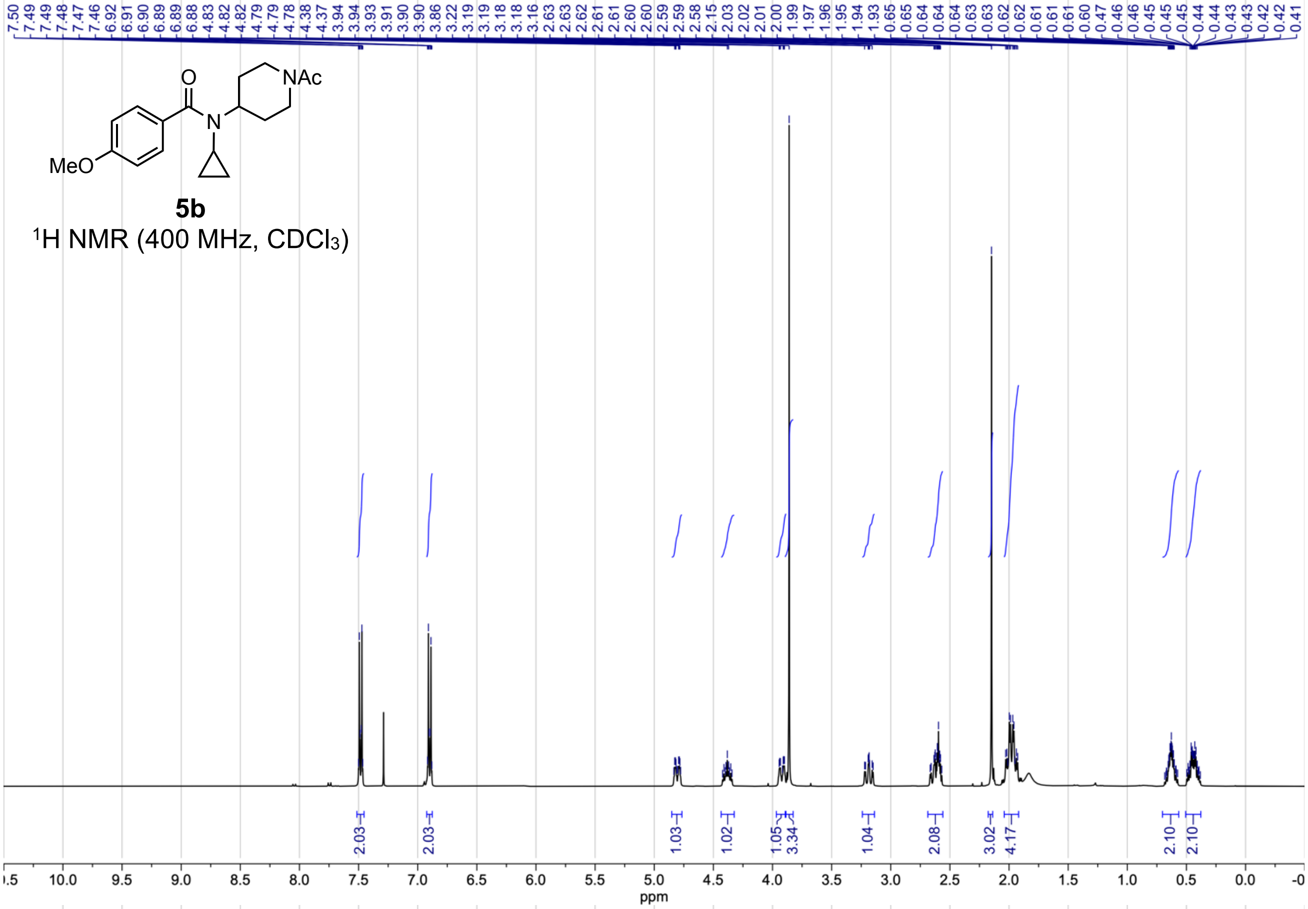
NAc
O
N
MeO
5b
¹H NMR (400 MHz, CDCl3)
2.03
2.03
1.03
1.02
1.05
3.34
1.04
2.08
3.02
4.17
2.10
2.10
ppm

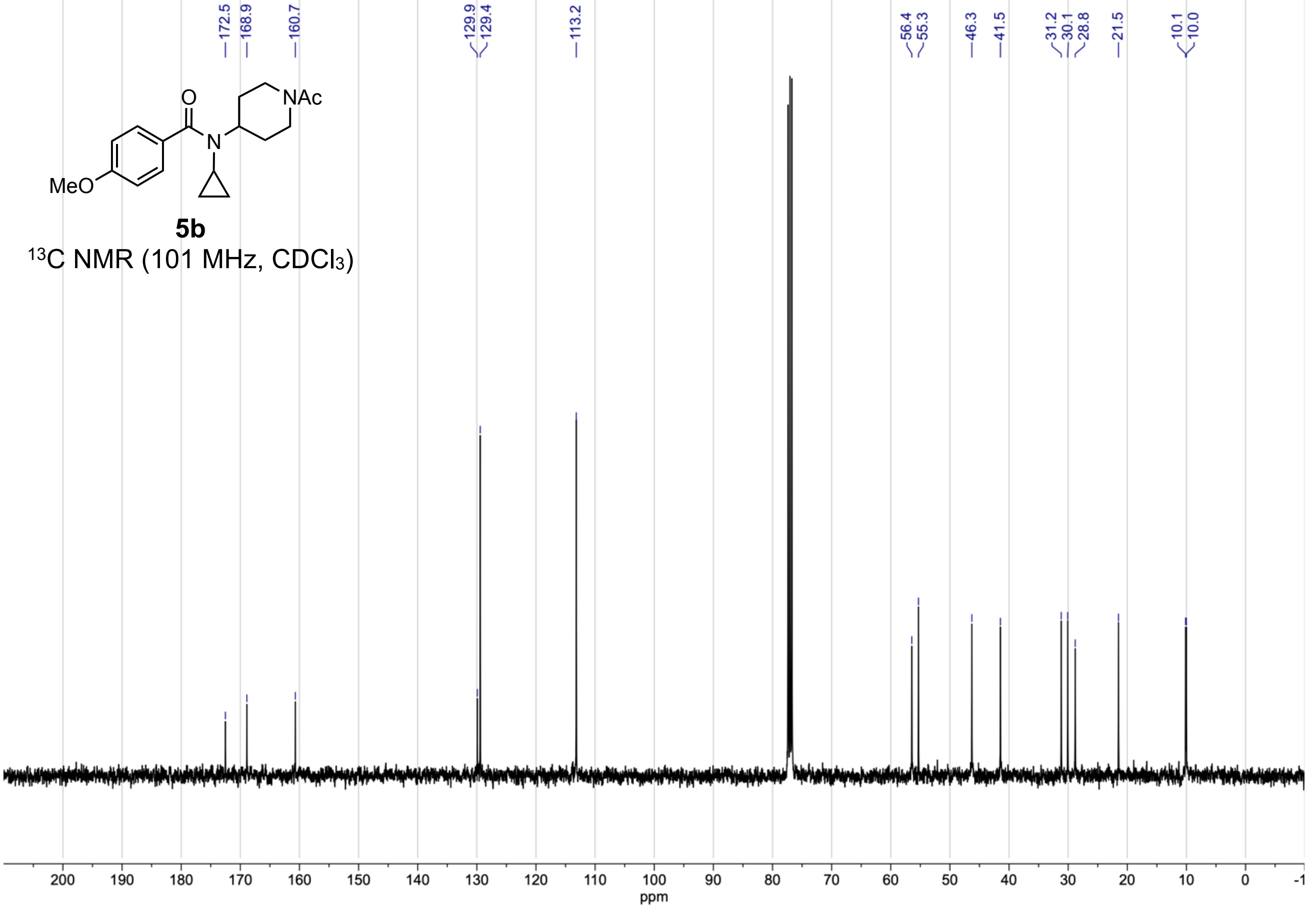
172.5
168.9
160.7
129.9
129.4
113.2
56.4
55.3
46.3
41.5
31.2
30.1
28.8
21.5
10.1
10.0
NAc
O
N
MeO
5b
¹³C NMR (101 MHz, CDCl3)
ppm

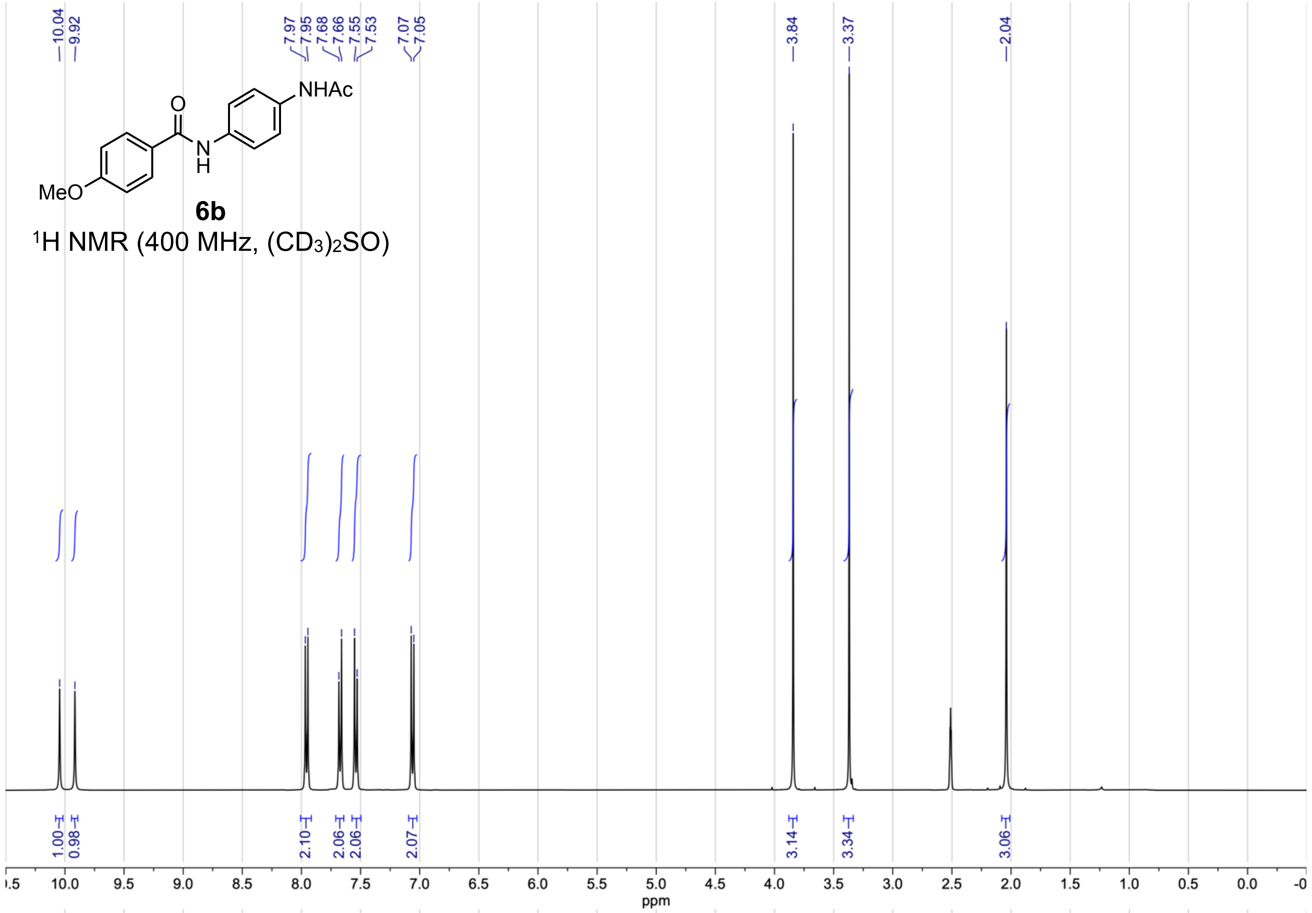
10.04
9.92
7.97
7.95
7.68
7.66
7.55
7.53
7.07
7.05
3.84
3.37
2.04
NHAc
O
N
H
MeO
6b
¹H NMR (400 MHz, (CD₃)₂SO)
1.00
0.98
2.10
2.06
2.06
2.07
3.14
3.34
3.06
ppm

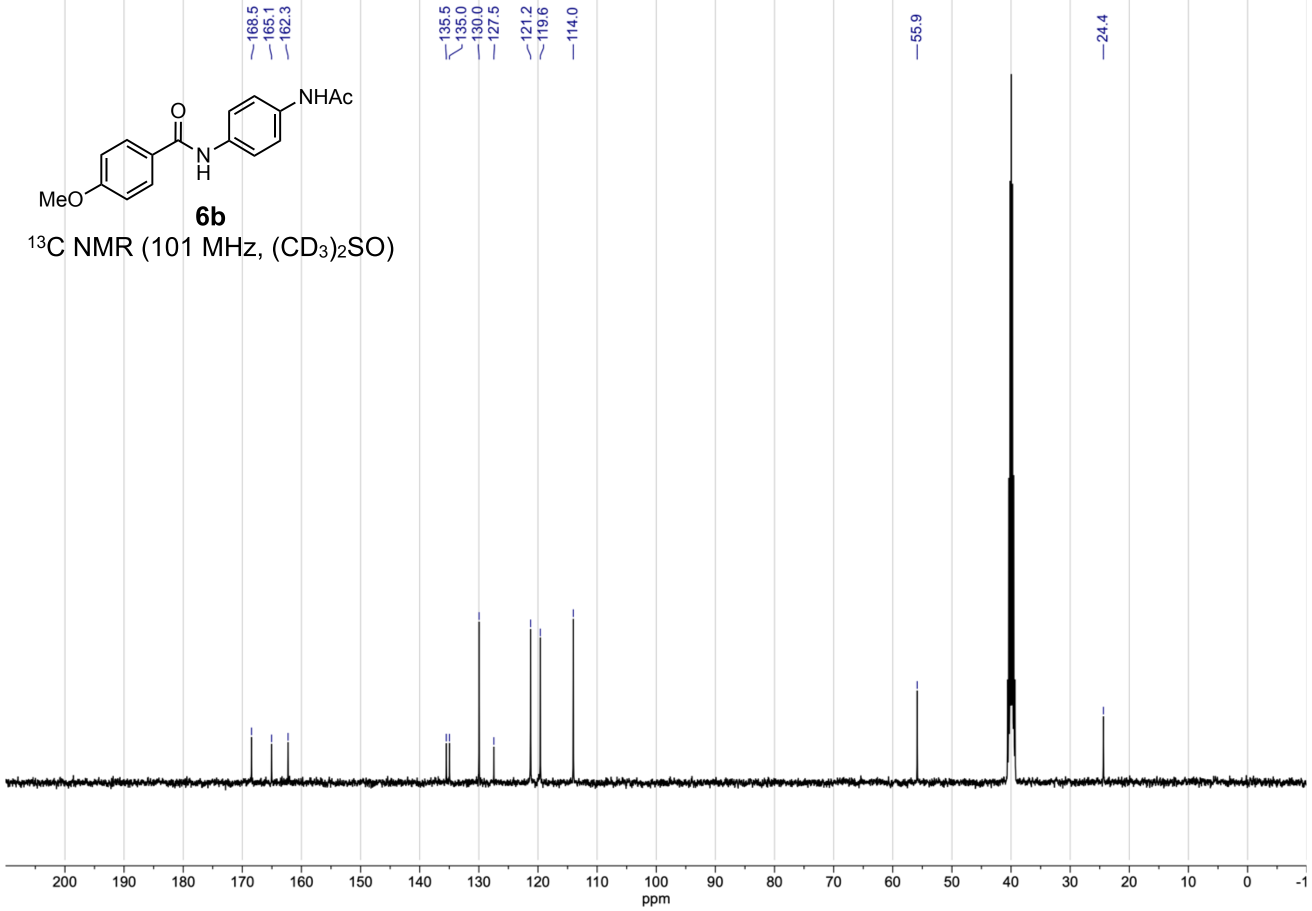
168.5
165.1
162.3
135.5
135.0
130.0
127.5
121.2
119.6
114.0
55.9
24.4
NHAc
O
N
H
MeO
6b
¹³C NMR (101 MHz, (CD₃)₂SO)
ppm

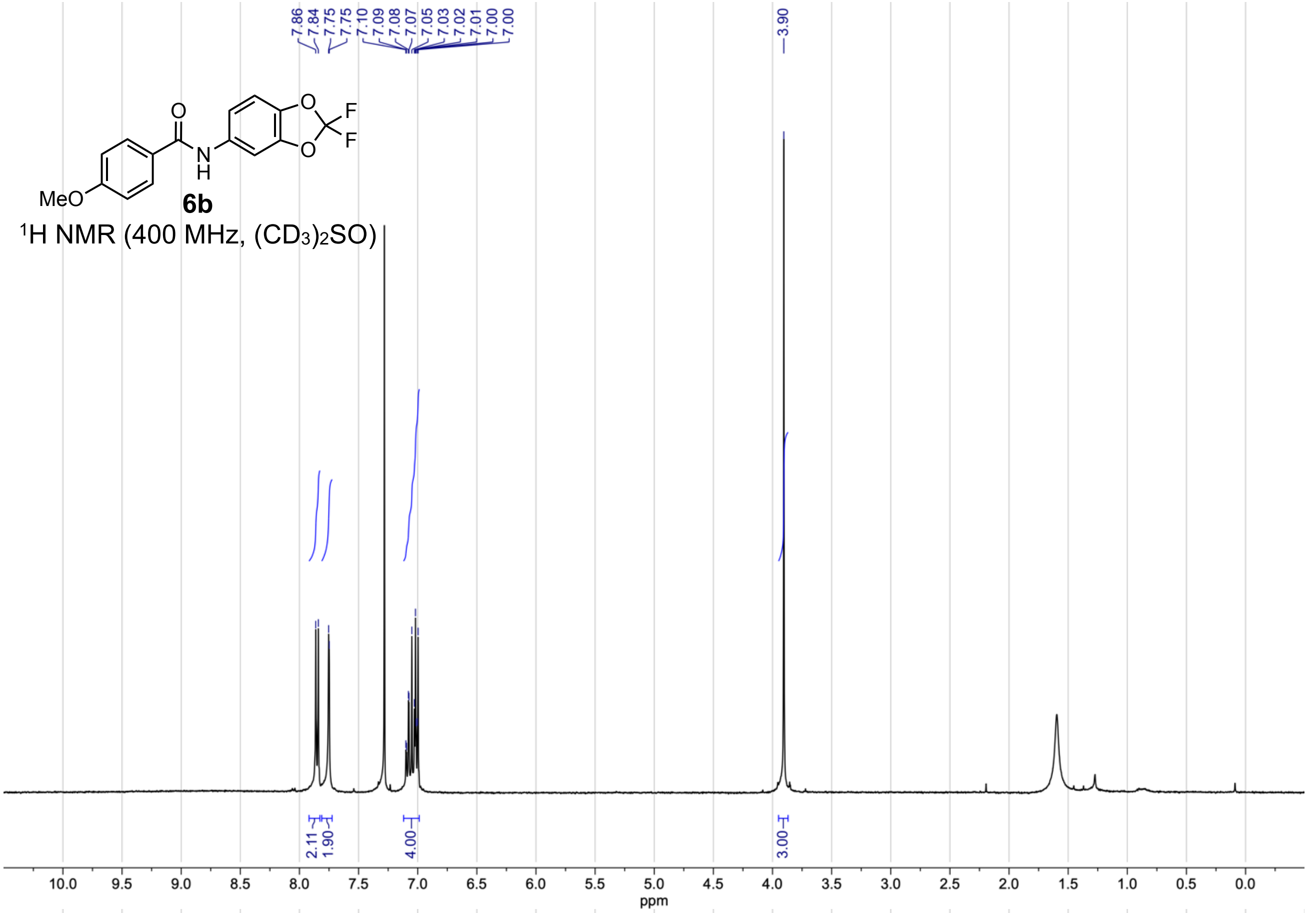
7.86
7.84
7.75
7.75
7.10
7.09
7.08
7.07
7.05
7.03
7.02
7.01
7.00
7.00
3.90
MeO
O
N
H
O
O
F
F
6b
^{1}H NMR (400 MHz, $(CD_3)_2SO$)
2.11
1.90
4.00
3.00
10.0 9.5 9.0 8.5 8.0 7.5 7.0 6.5 6.0 5.5 5.0 4.5 4.0 3.5 3.0 2.5 2.0 1.5 1.0 0.5 0.0
ppm

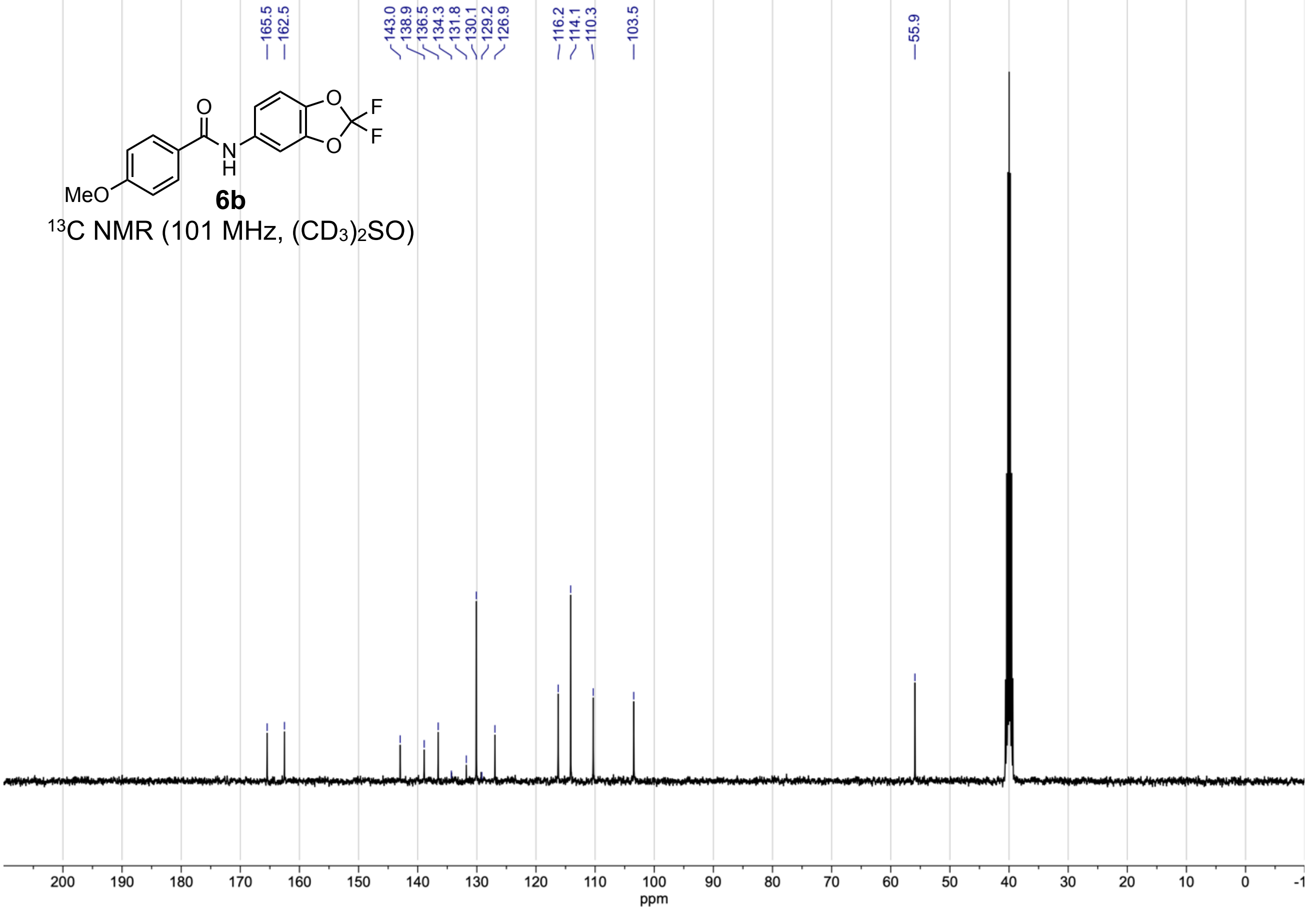
165.5
162.5
143.0
138.9
136.5
134.3
131.8
130.1
129.2
126.9
116.2
114.1
110.3
103.5
55.9
MeO
O
N
H
O
O
F
F
6b
^{13}C NMR (101 MHz, $(CD_3)_2SO$)
200 190 180 170 160 150 140 130 120 110 100 90 80 70 60 50 40 30 20 10 0 -1
ppm

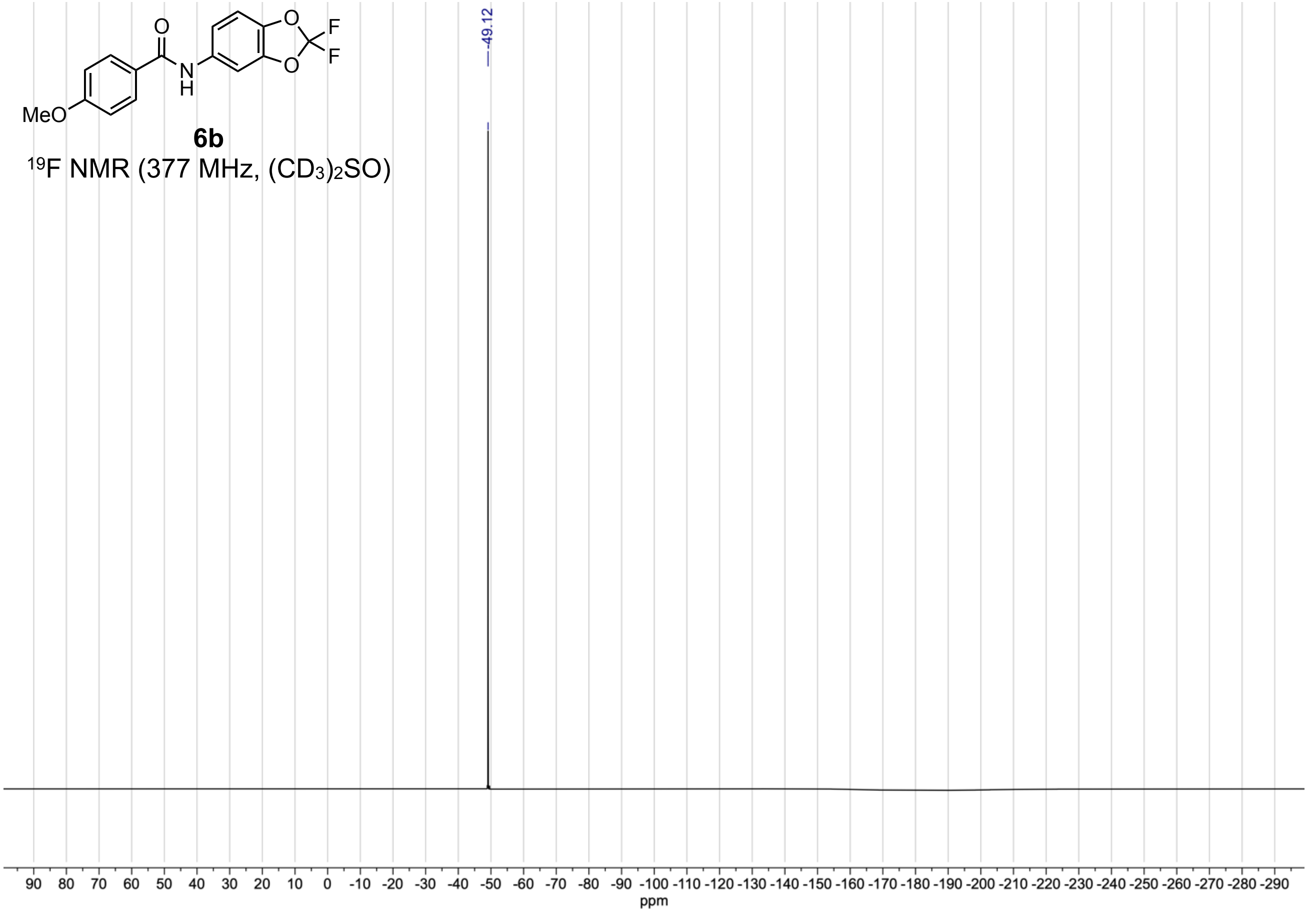

6b

^{19}F NMR (377 MHz, $(CD_3)_2SO$)

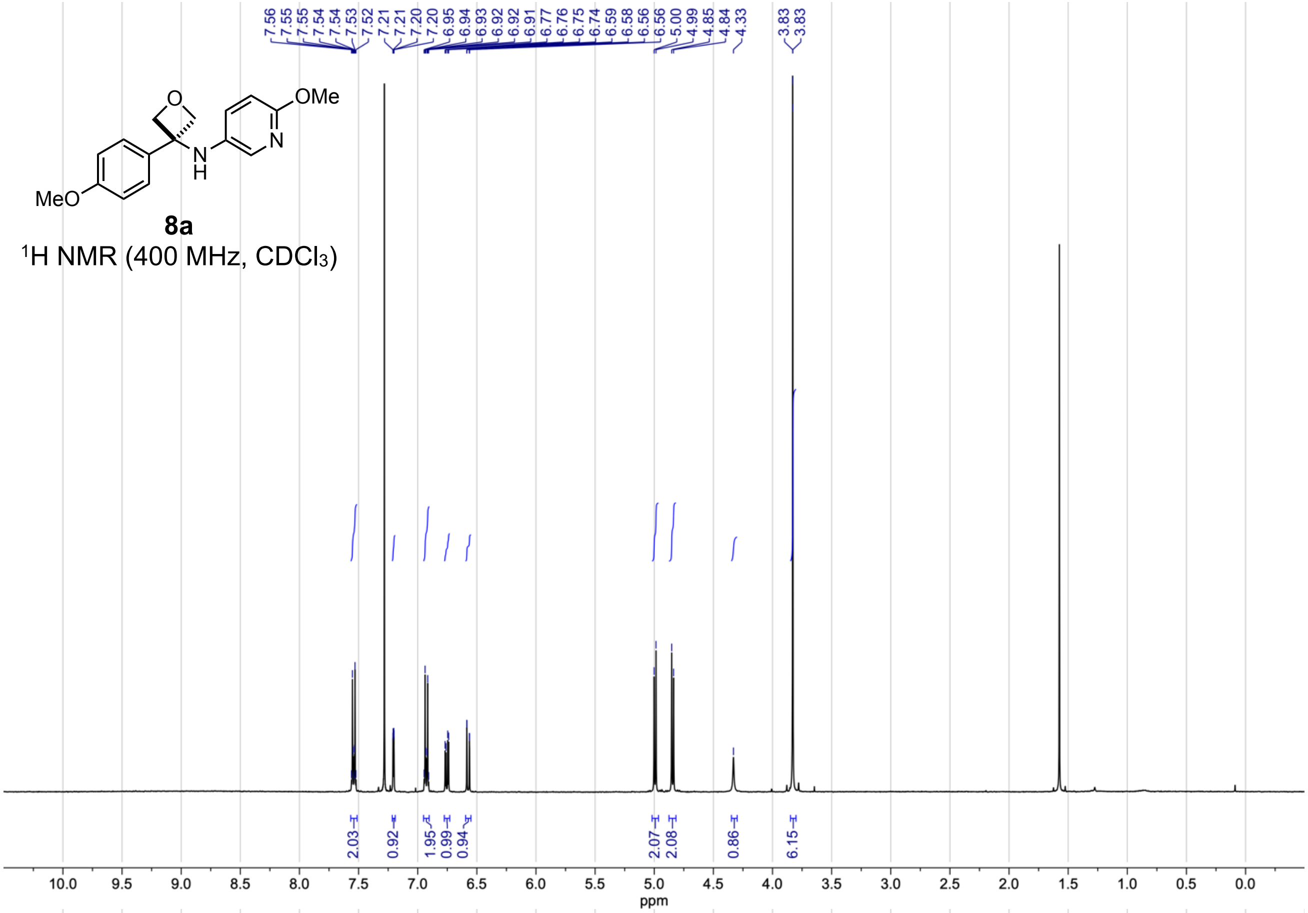

8a

^{1}H NMR (400 MHz, $CDCl_3$)

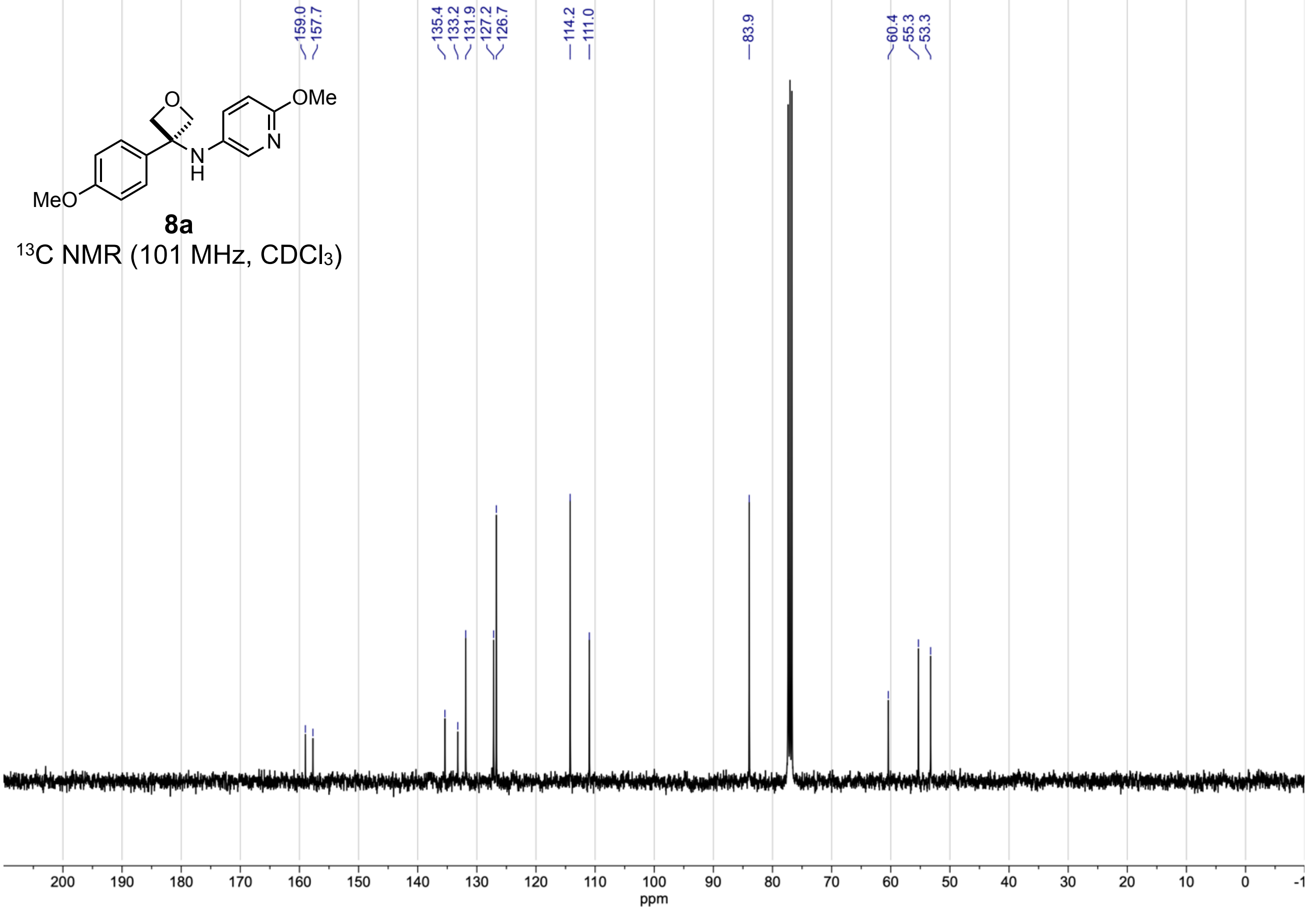

8a

^{13}C NMR (101 MHz, $CDCl_3$)

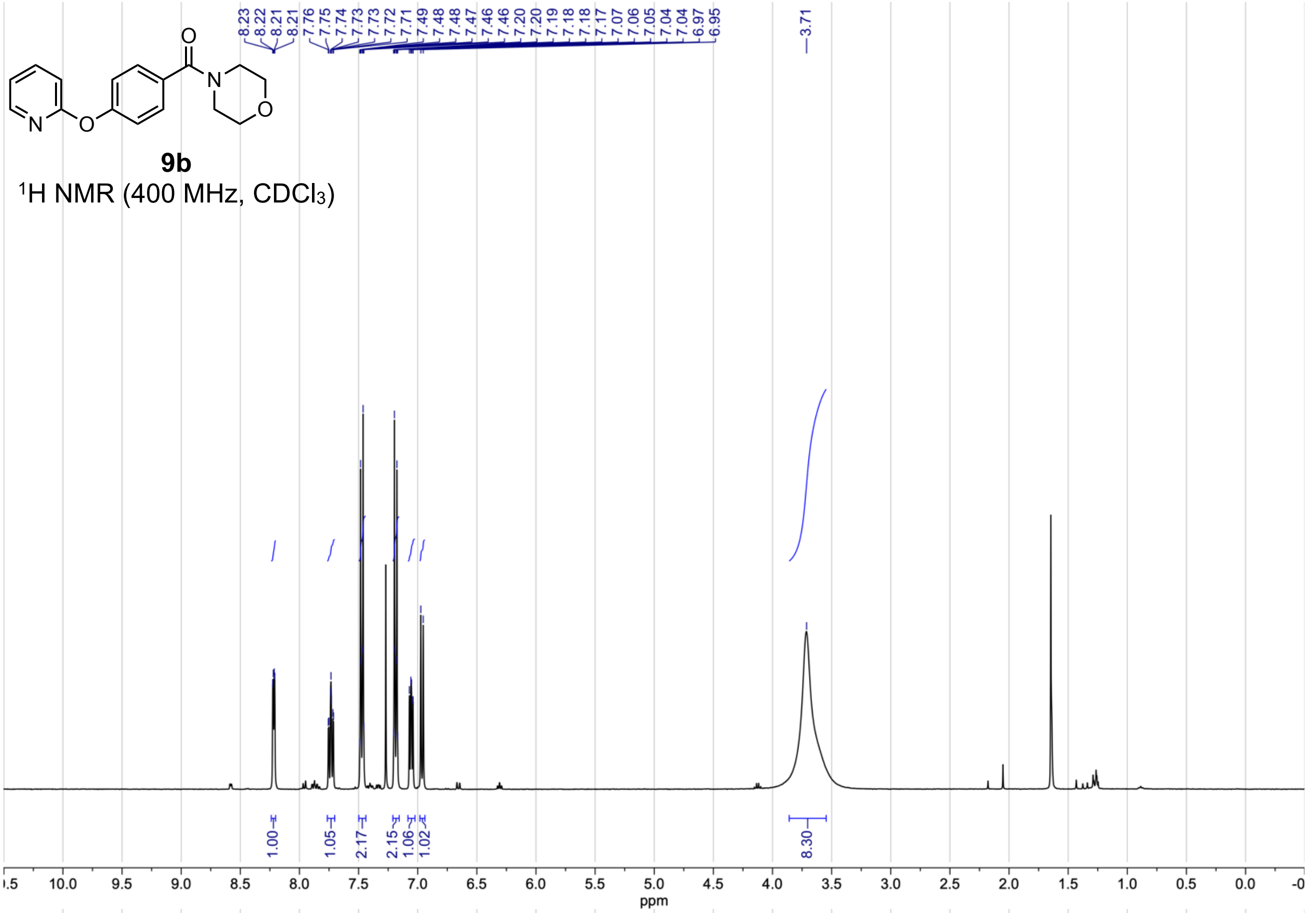
8.23
8.22
8.21
8.21
7.76
7.75
7.74
7.73
7.73
7.72
7.71
7.49
7.48
7.48
7.47
7.46
7.46
7.20
7.20
7.19
7.18
7.18
7.17
7.07
7.06
7.05
7.04
7.04
6.97
6.95
3.71
O
N
N
O
O
9b
1H NMR (400 MHz, CDCl3)
1.00
1.05
2.17
2.15
1.06
1.02
8.30
ppm

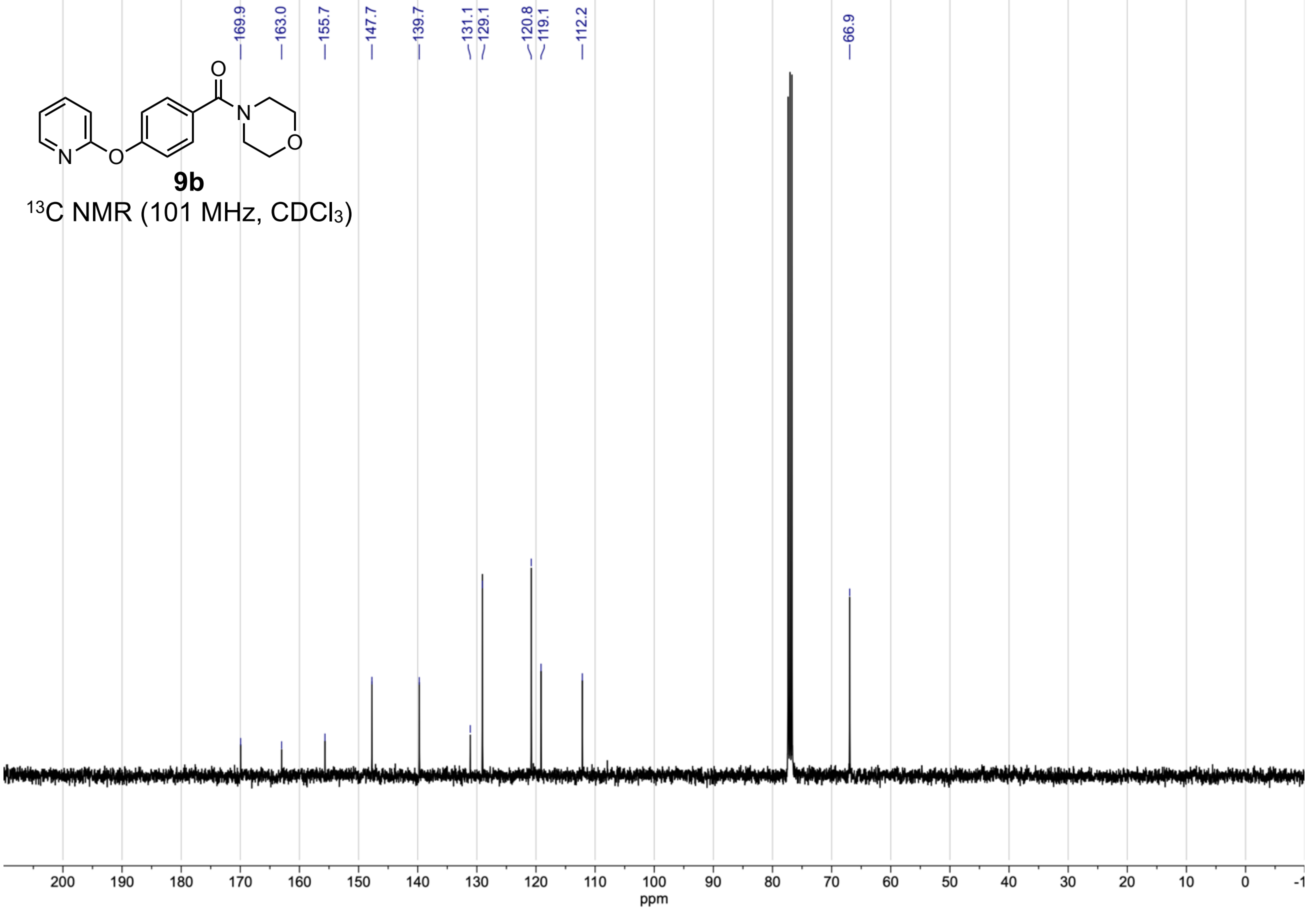
169.9
163.0
155.7
147.7
139.7
131.1
129.1
120.8
119.1
112.2
66.9
O
N
N
O
O
9b
13C NMR (101 MHz, CDCl3)
ppm

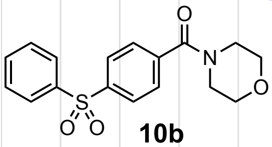

10b

^{1}H NMR (400 MHz, $CDCl_3$)

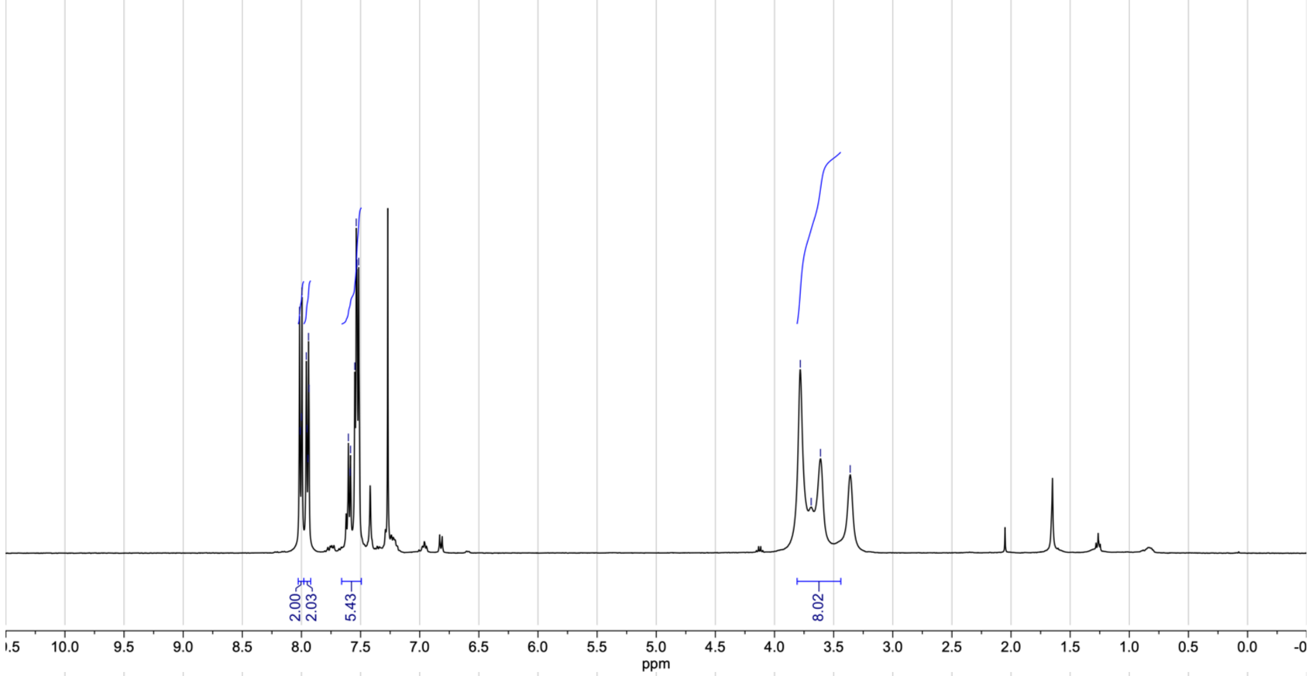

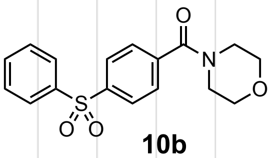

10b

^{13}C NMR (101 MHz, $CDCl_3$)

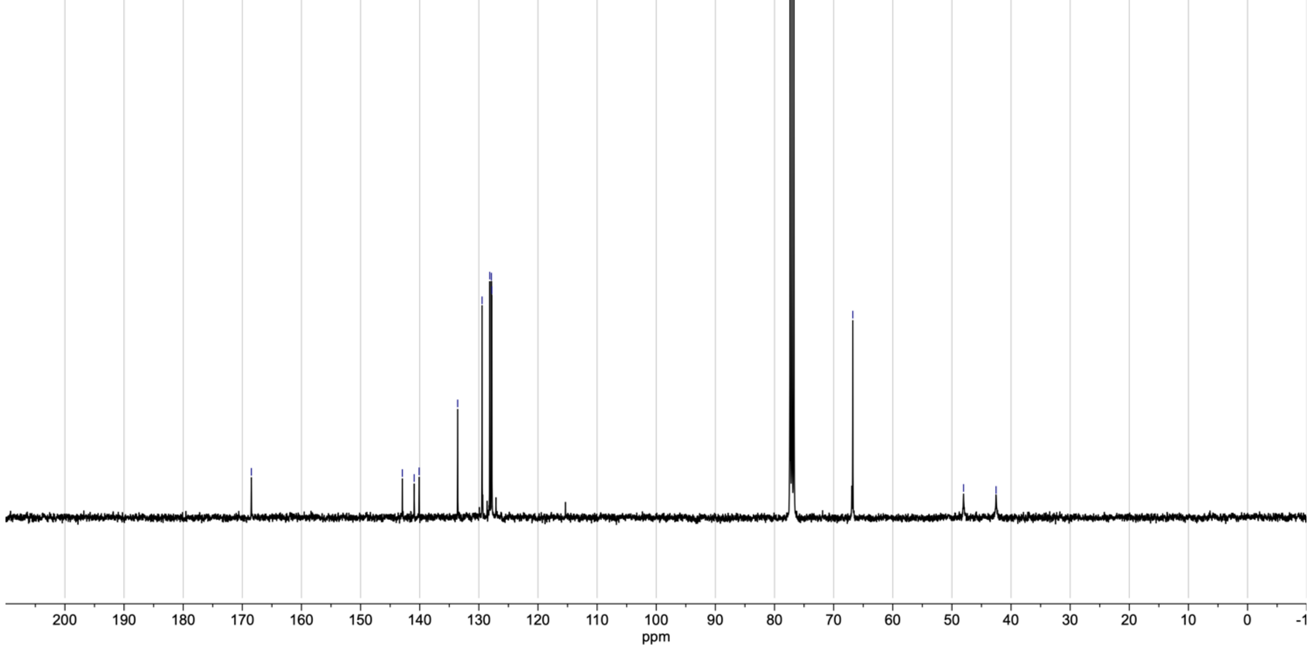

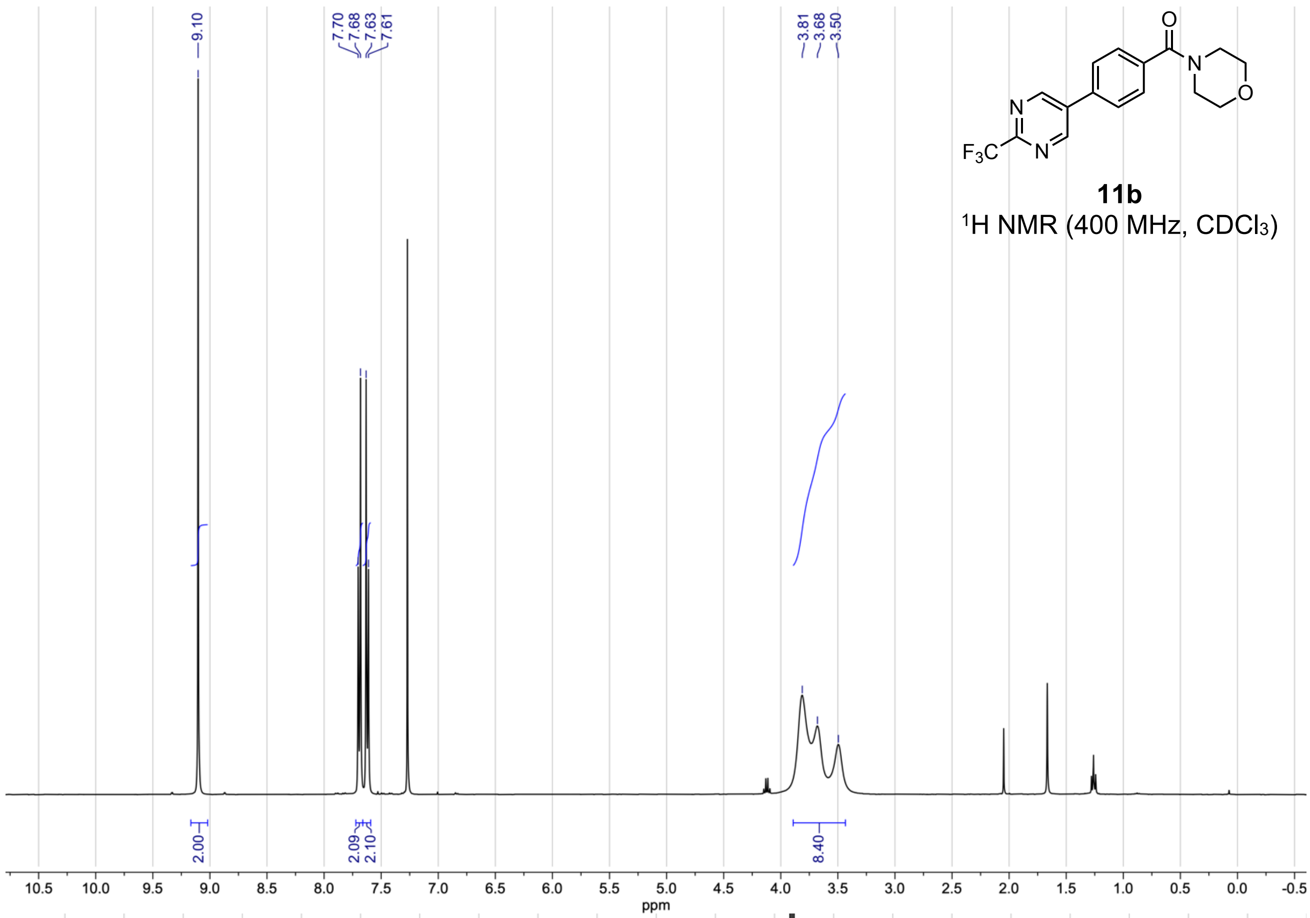
9.10
7.70
7.68
7.63
7.61
3.81
3.68
3.50
2.00
2.09
2.10
8.40
10.5
10.0
9.5
9.0
8.5
8.0
7.5
7.0
6.5
6.0
5.5
5.0
4.5
4.0
3.5
3.0
2.5
2.0
1.5
1.0
0.5
0.0
-0.5
ppm
O
N
O
N
N
F3C
11b
1H NMR (400 MHz, CDCl3)

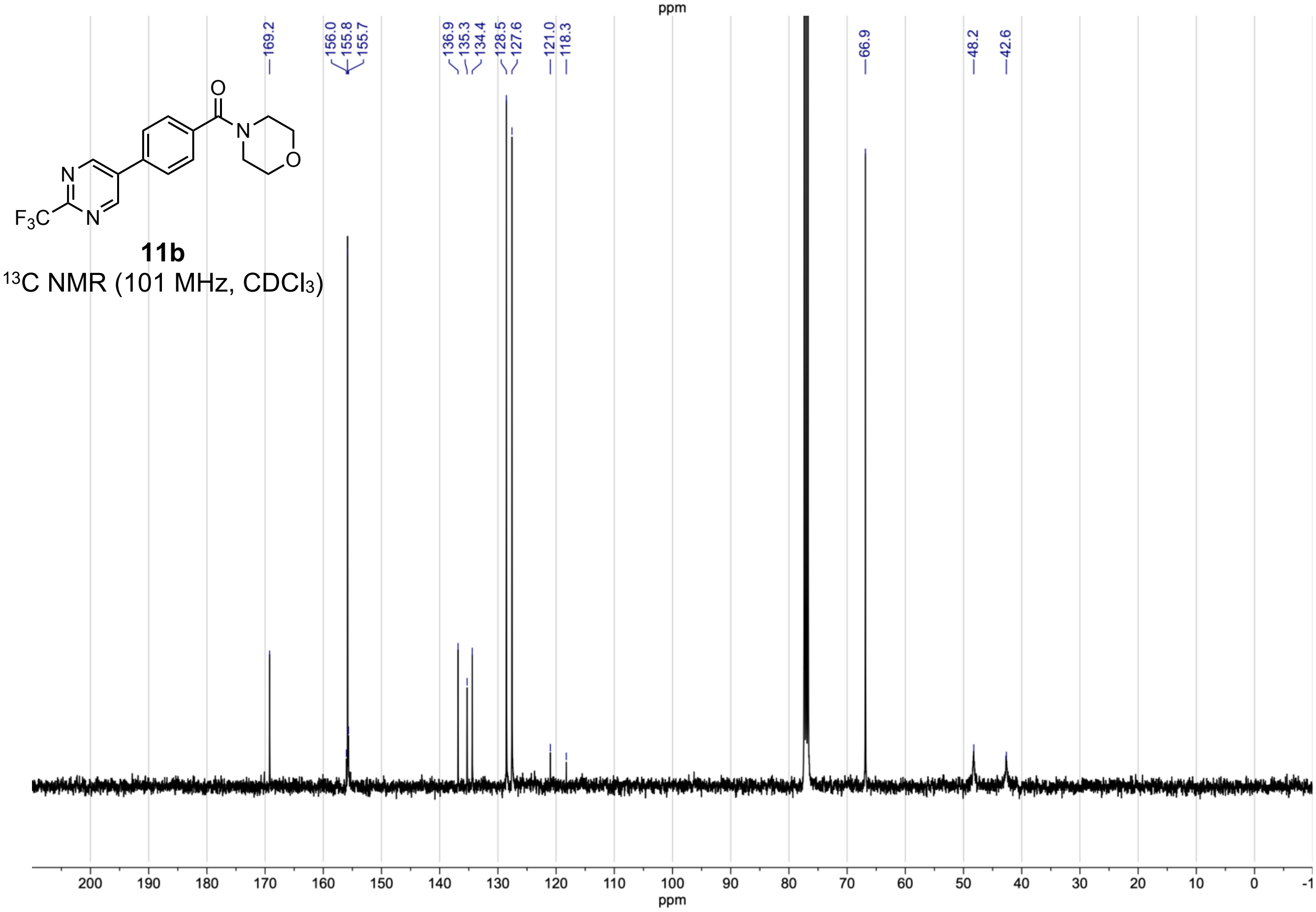
169.2
156.0
155.8
155.7
136.9
135.3
134.4
128.5
127.6
121.0
118.3
66.9
48.2
42.6
O
N
O
N
N
F3C
11b
13C NMR (101 MHz, CDCl3)
200
190
180
170
160
150
140
130
120
110
100
90
80
70
60
50
40
30
20
10
0
-1
ppm

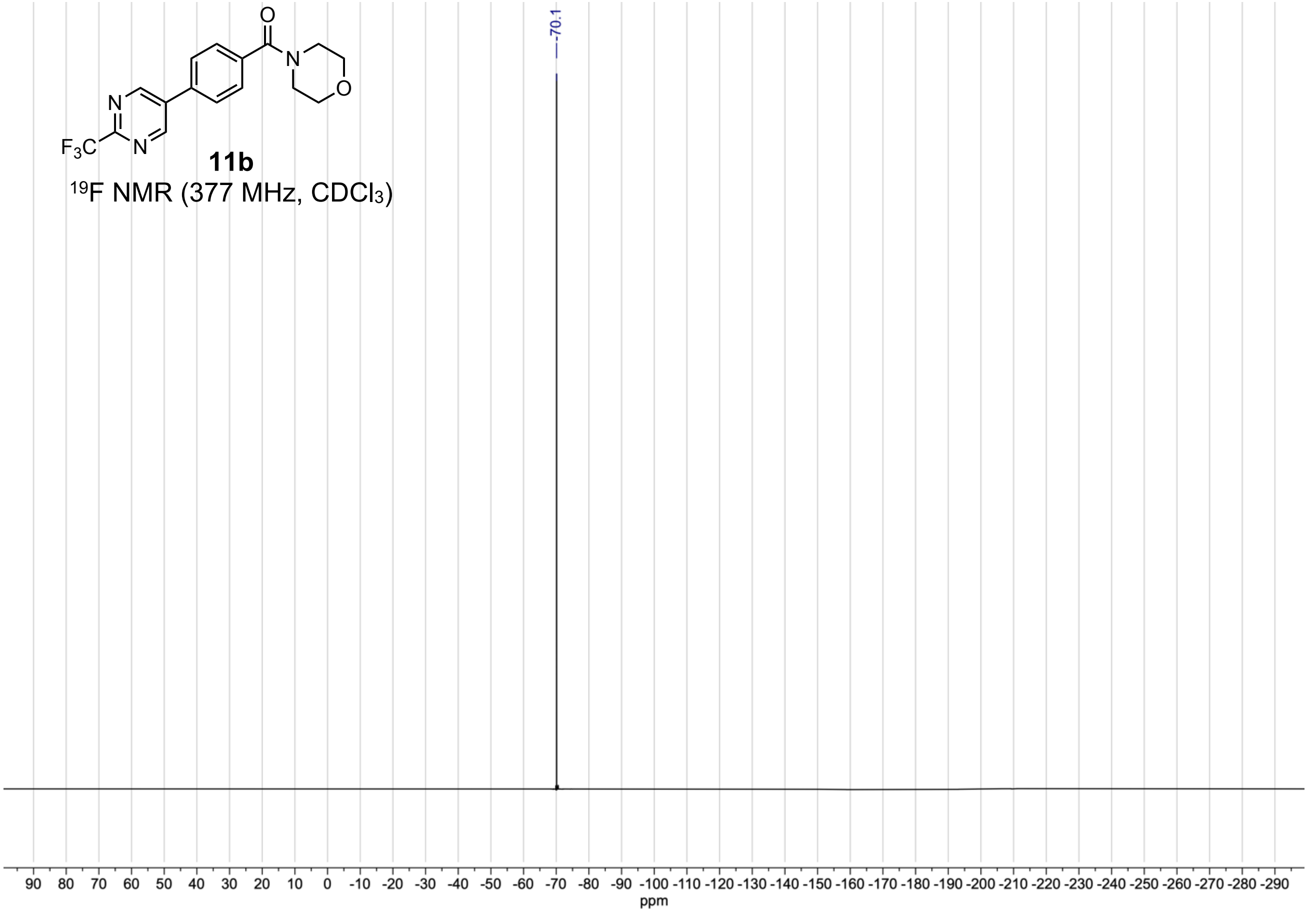

11b

^{19}F NMR (377 MHz, $CDCl_3$)
